# A multiplex protein panel assay for severity prediction and outcome prognosis in patients with COVID-19: An observational multi-cohort study

**DOI:** 10.1016/j.eclinm.2022.101495

**Published:** 2022-06-09

**Authors:** Ziyue Wang, Adam Cryar, Oliver Lemke, Pinkus Tober-Lau, Daniela Ludwig, Elisa Theresa Helbig, Stefan Hippenstiel, Leif-Erik Sander, Daniel Blake, Catherine S. Lane, Rebekah L. Sayers, Christoph Mueller, Johannes Zeiser, StJohn Townsend, Vadim Demichev, Michael Mülleder, Florian Kurth, Ernestas Sirka, Johannes Hartl, Markus Ralser

**Affiliations:** a Department of Biochemistry, Charité – Universitätsmedizin Berlin, Corporate Member of Freie Universität Berlin and Humboldt-Universität zu Berlin, Am Chariteplatz 1, 10117 Berlin, Germany; b Inoviv, Mappin House, 4 Winsley St, London, United Kingdom; c Department of Infectious Diseases and Respiratory Medicine, Charité – Universitätsmedizin Berlin, corporate member of Freie Universität Berlin and Humboldt-Universität zu Berlin, Augustenburger Platz 1, 13353 Berlin, Germany; d SCIEX, Macclesfield, United Kingdom; e Agilent Technologies Sales & Services GmbH & Co. KG, Waldbronn, Germany; f Core Facility – High-Throughput Mass Spectrometry, Charité – Universitätsmedizin Berlin, Corporate Member of Freie Universität Berlin and Humboldt-Universität zu Berlin, Am Chariteplatz 1, 10117 Berlin, Germany; g Department of Tropical Medicine, Bernhard Nocht Institute for Tropical Medicine, and Department of Medicine I, University Medical Centre Hamburg-Eppendorf, Hamburg, Germany; h The Molecular Biology of Metabolism Laboratory, The Francis Crick Institute, London, UK; i Berlin Institute of Health at the Charité - Universitätsmedizin Berlin, Berlin, Germany

**Keywords:** COVID-19, SARS-CoV2, Biomarker, Clinical disease progression, Severity stratification, Disease prognosis, Machine learning, Targeted proteomics, LC-MS/MS

## Abstract

**Background:**

Global healthcare systems continue to be challenged by the COVID-19 pandemic, and there is a need for clinical assays that can help optimise resource allocation, support treatment decisions, and accelerate the development and evaluation of new therapies.

**Methods:**

We developed a multiplexed proteomics assay for determining disease severity and prognosis in COVID-19. The assay quantifies up to 50 peptides, derived from 30 known and newly introduced COVID-19-related protein markers, in a single measurement using routine-lab compatible analytical flow rate liquid chromatography and multiple reaction monitoring (LC-MRM). We conducted two observational studies in patients with COVID-19 hospitalised at Charité – Universitätsmedizin Berlin, Germany before (from March 1 to 26, 2020, n=30) and after (from April 4 to November 19, 2020, n=164) dexamethasone became standard of care. The study is registered in the German and the WHO International Clinical Trials Registry (DRKS00021688).

**Findings:**

The assay produces reproducible (median inter-batch CV of 10.9%) absolute quantification of 47 peptides with high sensitivity (median LLOQ of 143 ng/ml) and accuracy (median 96.8%). In both studies, the assay reproducibly captured hallmarks of COVID-19 infection and severity, as it distinguished healthy individuals, mild, moderate, and severe COVID-19. In the post-dexamethasone cohort, the assay predicted survival with an accuracy of 0.83 (108/130), and death with an accuracy of 0.76 (26/34) in the median 2.5 weeks before the outcome, thereby outperforming compound clinical risk assessments such as SOFA, APACHE II, and ABCS scores.

**Interpretation:**

Disease severity and clinical outcomes of patients with COVID-19 can be stratified and predicted by the routine-applicable panel assay that combines known and novel COVID-19 biomarkers. The prognostic value of this assay should be prospectively assessed in larger patient cohorts for future support of clinical decisions, including evaluation of sample flow in routine setting. The possibility to objectively classify COVID-19 severity can be helpful for monitoring of novel therapies, especially in early clinical trials.

**Funding:**

This research was funded in part by the European Research Council (ERC) under grant agreement ERC-SyG-2020 951475 (to M.R) and by the Wellcome Trust (IA 200829/Z/16/Z to M.R.). The work was further supported by the Ministry of Education and Research (BMBF) as part of the National Research Node ‘Mass Spectrometry in Systems Medicine (MSCoresys)', under grant agreements 031L0220 and 161L0221. J.H. was supported by a Swiss National Science Foundation (SNSF) Postdoc Mobility fellowship (project number 191052). This study was further supported by the BMBF grant NaFoUniMedCOVID-19 – NUM-NAPKON, FKZ: 01KX2021. The study was co-funded by the UK's innovation agency, Innovate UK, under project numbers 75594 and 56328.

**Research in context**

**Evidence before this study**

We searched PubMed for articles published up to May 12, 2022. We used the search terms COVID-19 or SARS-CoV-2, and severity or outcome, and prognosis or prediction, and proteomics or protein panel or peptide panel, and plasma or serum, and mass spectrometry. The search returned 10 research articles, which mostly used explorative proteomics to identify putative protein markers associated with COVID-19 disease severity or outcome. However, no study translated identified biomarkers into a panel assay providing absolute quantification which can be deployed as targeted mass spectrometry platforms available to routine diagnostic laboratories.

**Added value of this study**

Proteomic panel assays hold the promise to outperform established intensive care unit outcome predictors such as APACHE II or SOFA in COVID-19, but so far their application in clinical routine is challenging for technical reasons.  We select a panel of 50 peptides, derived from 30 proteins, whose functions have been associated with COVID-19 using discovery proteomics, and develop and analytically validate a scalable proteomic panel assay that is performed on instrumentation common in clinical laboratories. Applying the assay to two independent cohorts, we demonstrate accurate disease classification, and show that the marker panel is prognostic about outcome.

**Implications of all the available evidence**

The potential value of using the human plasma proteome in severity classification, risk assessment, and outcome prediction in COVID-19 has recently been uncovered in several studies. What was missing so far was translation of this research into a routine applicable assay. We present a protein marker panel which predicts survival in COVID-19 with high accuracy that can be implemented for routine laboratory testing. The described assay has the potential to improve clinical risk assessment for patients with COVID-19 by translating discovery proteomics findings to patient care.

Alt-text: Unlabelled box

## Introduction

COVID-19 challenges healthcare systems worldwide, which is particularly apparent in areas with limited vaccine uptake. The outlook remains uncertain even in countries with high vaccination rates as the immunity conferred by the vaccines appears to diminish over time.^1^, ^2^, ^3^, ^4^, ^5^ Moreover, SARS-COV-2 variants with capacity to evade immunity continue to emerge,^1^^,^^6^, ^7^, ^8^ and may affect global medical care rapidly and unpredictably.

Biomarker tests that classify disease severity and are prognostic could help mitigate the impact of critical treatment choice by allowing to optimise resource allocation.^9^, ^10^, ^11^ Indeed, clinical manifestation of COVID-19 is highly variable. For instance, ‘happy hypoxia’ describes situations where patients with COVID-19 present in a relatively well compensated clinical status, while molecular indicators indicate they are, in fact, severely ill.^12^ Furthermore, in situations when healthcare systems reach maximum capacity, prognostic tests could support difficult clinical decisions, for instance to identify individuals that require maximum available support, irrespective of age and comorbidities.^13^ Prognostic and severity-classifying tests could further help increase the likelihood of success and accelerate clinical trials by improving treatment efficacy assessments of COVID-19 therapies or stratifying patient populations for inclusion into the trials. They might also help detect clinically yet inapparent side effects and contribute to patient safety. Moreover, during a pandemic, there is a need to conduct trials in a timely manner, and often in cohorts of limited size. When underpowered, clinical trials can lead to false positive and false negative assessments of a drug's efficacy.^14^^,^^15^ Disease-severity and prognostic tests could hence help extrapolate more and patient-specific information. Unfortunately, the reliability of several risk-assessment scores conventionally used in ICU settings such as the Acute Physiology And Chronic Health Evaluation (APACHE II), Charlson Comorbidity Index (CCI), and Sequential Organ Failure Assessment (SOFA) scores appears to be limited in COVID-19.^16^ Combinations of generic clinical readouts, e.g. blood oxygen saturation and interleukin-6 concentration, have been considered for outcome prediction at various disease-severity stages.^17^ However, several predictive models that were reported early in the pandemic are now considered to be vulnerable to bias, can be obsolete due to therapeutic measures such as CRP-based models after anti-IL-6 treatment, and might not be suitable for the clinic.^18^^,^^19^ Therefore, additional COVID-19-specific compound scores have been proposed recently (e.g. ABCS^20^), showing improved performance. A remaining limitation of compound scores is their reliance on various measurements of different nature, which makes them statistically challenging.

Proteomic datasets have repeatedly been successful at classifying and predicting COVID-19 severity and outcome,^9^^,^^10^^,^^16^^,^^21^, ^22^, ^23^ and can quantify many proteins in parallel, from one sample and one measurement. Indeed, specifically in severe COVID-19 cases, proteomic predictors have outperformed APACHE II, CCI, and SOFA scores.^11^^,^^13^^,^^24^^,^^25^ Proteomics also accelerated the characterisation of the antiviral host response, which improved our understanding of the COVID-19 disease by attributing the complement cascade, coagulation system, and apoprotein function to differences in COVID-19 pathology.^9^^,^^10^^,^^16^^,^^21^, ^22^, ^23^^,^^25^ The application of discovery proteomics as a test for the clinical routine is, however, limited for technical, economic, and regulatory reasons.

The objective of this study was to develop a COVID-19 biomarker panel assay which runs on broadly available analytical instruments that could be deployed for clinical use within existing regulatory frameworks. Triple quadrupole mass spectrometers coupled to high-flow liquid chromatography are used in the clinic in other areas^26^, ^27^, ^28^, ^29^ and are widely available in large hospital laboratories, diagnostic laboratories, regulated (e.g. CLIA) laboratories, and contract research organisations. Biomarker tests developed on this platform can be accredited to existing regulatory standards in GCP, ISO:17025, ISO:15189, and CLIA environments, standardised and transferred across different instruments and laboratories, and thus deployed at scale rapidly. Triple-quadrupole-mass-spectrometry-based tests are cost effective to run at scale as sample preparation can be automated, consumables costs for the MS runs are typically <£10 per test, and the instrument uptime is typically >95%.

In order to establish a proteomic panel assay using analytical flow rate chromatography and multiple reaction monitoring on triple quadrupole instruments, we have mined discovery proteomics data from patients with COVID-19 and selected biomarkers that are informative about COVID-19 disease progression. The biomarkers were chosen for i) being prognostic of remaining duration of hospitalisation, disease aggravation, or being differentially concentrated in plasma depending on the treatment escalation level, used as a measure of disease severity, and ii) participating in biological processes that contribute to COVID-19 pathology, and iii) being technically and analytically suitable for the assay. Employing calibration curves with synthetic reference and stable-isotope-labelled (SIL) internal standards, the assay aims for the absolute quantification of up to 50 surrogate tryptic peptides corresponding to 30 plasma proteins. These function in inflammation (e.g. C-reactive protein), coagulation and vascular dysfunction (e.g. von Willebrand factor), complement cascade (e.g. Complement C1q subcomponent subunit C), and other biological processes altered by COVID-19 (e.g. Cystatin C).

We analytically validated the assay and implemented it in two analytical laboratories employing two different triple quadrupole LC-MS/MS platforms. The assay was then applied to two observational cohorts. We demonstrate that the assay captures host response to SARS-CoV-2 and thereby classifies and predicts COVID-19 disease severity. In one cohort, we tested the prognostic value of the panel. We found that the biomarker panel is predictive about survival weeks before outcome, and outperforms several commonly used risk-assessment scores.

## Methods

### Study design and participants

Patient samples were collected as part of an observational cohort.^13^^,^^24^^,^^25^ The study protocol, patient characteristics, treatment and outcomes were described previously.^30^, ^31^, ^32^ Briefly, all in-patients with PCR-confirmed SARS-CoV-2 infection treated at Charité – Universitätsmedizin Berlin, a tertiary care centre, were eligible for inclusion, regardless of age, gender, or disease severity, after written informed consent was obtained. We also included severely ill patients on invasive ventilation based on a deferred consent procedure in order to avoid bias towards mildly ill patients. The study size is based on availability of patients and feasibility, and all study patients were included in the analyses. An overview of the study design, cohorts, sampling, methodology and data analysis is provided in Supplementary Figure 1. Study date cutoffs were 1st to 26th of March 2020 (Cohort 2), and 4th April to 19th November 2020 (Cohort 3). The study is registered in the German and the WHO international registry for clinical studies (DRKS00021688). The study was approved by the ethics committee of Charite´ - Universitatsmedizin Berlin (EA2/066/20). The cohorts are summarised in Table 1 and Supplementary Table 1.

**Table 1:** Description of study cohort 3.

N	164
**Sex**	
female, n (%)	39 (23.8)
male, n (%)	125 (76.2)
**age, median [IQR]**	60 [51-69]
**body mass index, median [IQR]**	29.4 [24.7-32.5]
**maximum severity**	
WHO3, n (%)	23 (14.0)
WHO4, n (%)	42 (25.6)
WHO5, n (%)	34 (20.7)
WHO6, n (%)	3 (1.8)
WHO7, n (%)	28 (17.1)
WHO8 (deceased), n (%)	34 (20.7)
**treatment**	
Dexamethasone, n (%)	112 (68.3)
Remdesivir	15 (9.1)
IMV, n (%)	61 (37.2)
ECMO, n (%)	33 (20.1)
RRT, n (%)	24 (14.6)
**days hospitalized**	16 [10-34]
**days until discharge (w/o deceased)**	12 [8-29]
**days until death**	32 [19-47]
**sampling**	
days since symptom onset, median [IQR]	13 [8-17]
days to outcome, median [IQR]	10 [5-24]

### Reagents and peptide standards

Reference peptide standards were custom synthesised where native peptides were obtained at ≥95% purity and stable isotope-labelled (SIL) internal standard peptides (ISTDs) - at ≥70% purity. Internal standards contained 4-6 amino acid tryptic tags mimicking the sequence in a corresponding human plasma protein and were labelled on C-terminal lysine (K) or arginine (R) with stable isotopes (K(U-^13^C_6_,^15^N_2_) or R(U-^13^C_6_,^15^N_4_)). All peptide stock solutions were prepared at 1 mg/ml in 50:50 v/v ddH_2_O: acetonitrile mix, except for STDYGIFQINSR and VEGTAFVIFGIQDGEQR where 200 µl of DMSO were added to solubilise the peptides at 5 mg/ml which were then aliquoted and diluted to 1 mg/ml with 50:50 v/v ddH_2_O: acetonitrile mix. Internal standard mix was prepared by pooling 20 µl of each SIL peptide, evaporating 200 µl of this mix to dryness and reconstituting in a denaturation buffer to the final concentration of 1.4 µg/ml for each peptide. Cassetted calibration curves were prepared by serial dilution of pooled native reference peptide standards as described below. After serial dilution, these samples were treated identically to respective clinical samples. Additional reagents employed are listed in the Supplementary Methods.

### Sample preparation

Samples were prepared with minor modifications as described previously.^24^ Briefly, samples were stored at -80°C for 11-12 months prior to preparation, and clinical samples and calibration lines were prepared as follows: 5 µl of citrate plasma were added to 55 µl of denaturation buffer, composed of 50 µl 8 M Urea, 100 mM ammonium bicarbonate, 5 µl 50 mM dithiothreitol (DTT) and internal standard mix. The samples were incubated for 1 h at room temperature (RT) before addition of 5 µl of 100 mM iodoacetamide (IAA). After a 30 min incubation at RT the samples were diluted with 340 µl of 100 mM ammonium bicarbonate and digested overnight with 22.5 µl of 0.1 µg/µl trypsin at 37 °C. The digestion was quenched by adding 50 µl of 10% v/v formic acid. The resulting tryptic peptides were purified on a 96-well C18-based solid phase extraction (SPE) plate (BioPureSPE Macro 96-well, 100mg PROTO C18, The Nest Group). The purified samples were resuspended in 120 µl of 0.1% formic acid and 20 µl or 0.2 µl were injected into two LC-MS/MS platforms (Agilent 6495C and SCIEX 7500 respectively).

Samples in Cohort 3 were prepared as described above, with the following modifications. Samples were stored at -80°C for 5-11 months prior to preparation EDTA plasma was used instead of citrate plasma, and internal standards were digested separately and added to pre-digested clinical and calibration samples before their injection into the LC-MS/MS system. Quality control (QC) samples consisted of pooled commercial control and COVID-19 human plasma (as described in a previous publication^24^), and were prepared alongside clinical and calibration curve samples in each cohort.

The COVID-19 sample pools used for the analytical validation were generated by pooling 5 µl of patient plasma from Cohort 3 according to their WHO treatment severity score. Only samples of patients that had not received dexamethasone at time of sampling were used.

### Liquid chromatography - tandem mass spectrometry

Tryptic peptides were quantified on two LC-MRM platforms. All samples were analysed on the Agilent 6495C mass spectrometer, coupled to an Agilent 1290 Infinity II UHPLC system. Samples from Cohort 2 were additionally analysed on a SCIEX 7500 mass spectrometer coupled to an ExionLC AD UHPLC system (SCIEX, UK). Details on chromatography and mass spectrometry settings are described in the Supplementary Methods. MRM parameters are provided in the Supplementary Table 2 (Agilent 6495C) and Supplementary Table 3 (SCIEX 7500).

### Establishment of the MRM based assay

The assay was first set up on the 6495C (Agilent) system. Preliminary transitions for the 50 selected peptides (consisting of several precursor ion charge states and respective product ions) were predicted by Skyline v21.1.0.146.^33^ The native peptide standard solution was then infused into LC-MS/MS system and 1 precursor ion per peptide with the highest relative intensity and 5 most abundant product ions were selected for collision energy optimisation using Skyline. From these 5 product ions, 2-5 experimentally optimised ion transitions per native peptide were ultimately selected for the panel based on the following criteria: i) highest relative signal intensity, ii) optimal chromatographic peak shape and iii) absence of interfering signals. Product ions of <300 m/z were excluded where possible to ensure specificity. Precursor and product ion-matched ISTD transitions were also included. Lastly, all selected transitions were combined into one scheduled MRM method, where the most abundant transition for each peptide was used for quantification, and 1-4 remaining transitions - for qualification. (Supplementary Table 2). For analytical cross-platform and cross-laboratory validation, the assay was set up on the 7500 (SCIEX) system in parallel following this approach (Supplementary Table 3).

### Mass spectrometry data processing and calibration

LC-MRM data was processed using MassHunter Quantitative Analysis, v10.1 (Agilent platform) or SCIEX OS v2.0.1 (2020, Sciex platform). Peptide absolute concentration (expressed in ng/ml) was determined from calibration curves, constructed with native and SIL peptide standards, and manually validated. Linear regression analysis of each calibration curve was performed using custom R code or SCIEX OS (with 1/x weighting). The transitions used for quantification are shown in Supplementary Tables 2 and 3 respectively. Matching of native peptides and internal standards is detailed in the Supplementary Methods.

### Analytical method validation

Method analytical validation was performed based on FDA Bioanalytical Method Validation criteria^34^ where sensitivity, specificity, intra- and inter-batch precision, accuracy and matrix effects have been assessed. Five (5) independent calibration curves were prepared by serial dilution of native peptide standards in assay buffer (1), surrogate matrix (3) and pooled human plasma (1) across the final peptide concentration range of 0 - 146.7 µg/ml. Surrogate matrix (40 mg/ml bovine serum albumin (BSA)) calibration curves were prepared and analysed across 3 separate batches and all calibration curve samples were analysed in quintuplets. Linear 1/x weighted regression was used to test the linearity of the response of all calibration lines. To determine the intra- and inter-batch precision, the CV was calculated from the response ratios (native peptide peak area divided by ISTD area).

Lower limit of quantification (LLOQ) was defined as the lowest concentration point on the linear calibration curve where the inter-batch CV was ≤ 20%. Since analytical validation requirements for clinical assays are purpose and context dependent, and are influenced by the magnitude of change of target analyte levels in control versus disease samples, LLOQ CV cutoff was subsequently expanded to ≤40% for the remaining peptides. Upper limit of quantification (ULOQ) was defined as the highest calibration sample on the linear curve with a CV ≤ 20%.

Accuracy was assessed by treating 1 of the 5 replicates in each calibration curve in the surrogate matrix as pseudo-unknown samples, quantifying with the curve generated from the remaining 4 replicates, and then calculating a median accuracy of all replicates. Matrix effects were measured by comparing the slopes of calibration curve samples prepared in a BSA matrix and pooled human plasma. Here an Extra Sum of Square F test was used for statistical comparison with a p-value < 0.05 indicating potential matrix effects.

### Statistical analysis

Details of statistical tests performed in this study are available in the Supplementary Methods; test results are provided in Supplementary Tables 4-6. (Adjusted) *P* values were considered significant when *P* < 0.05. In brief, significance testing of the trend between absolute peptide concentrations and the ordinal classification as provided by the WHO treatment escalation scale was performed using Kendall's tau (KT) statistics and where indicated, with multiple testing correction. Cross-laboratory/cross-instrument performance was evaluated by Pearson correlation coefficients. Statistical tests on shotgun plasma proteomics data were performed as described.^13^

### Prediction of WHO grade and disease outcome

Clinical scores were extracted from the clinical information system or, where missing, manually calculated. CCI and APACHE II were determined at time of admission, SOFA (ICU patients only) at time of sampling, and ABCS at both admission and sampling. Note that due to imputation of the ABCS score memory leakage between training and test data for the ABCS score models can not be excluded for this particular comparison.

For the WHO grade and the outcome prediction a Support Vector Machine with rbf-kernel was constructed on the first sample measured for every patient (n = 164). The model was trained and validated using a shuffled stratified 10-fold cross-validation to avoid data leakage between training and validation data. For models trained on established risk assessments scores, only samples for which the respective score was determined were included in model construction and testing.

In addition, predictors based on logistic regression and the extra-trees algorithm were evaluated as well. Feature importances were extracted from a model trained on all data (n=164) without splitting the data set. Detailed information on model construction, evaluation and metric calculations are reported in Supplementary methods.

### Recommendations and guidelines

Mischak et al.^35^ have described a set of practical recommendations for biomarker discovery in clinical proteomics. We have provided an assessment of this study with respect to therein stated reporting recommendations in Supplementary Table 7. This study further follows the *Strengthening the Reporting of Observational Studies in Epidemiology* (STROBE) reporting guideline for observational studies.^36^

### Role of the funding source

The funding sources had no involvement in study design, data collection, or the manuscript. All authors reviewed the manuscript and had access to the data generated in the study. FK, ES, JH, and MR were responsible for the decision to submit the paper for publication.

## Results

### Peptide selection

**Figure 1:**
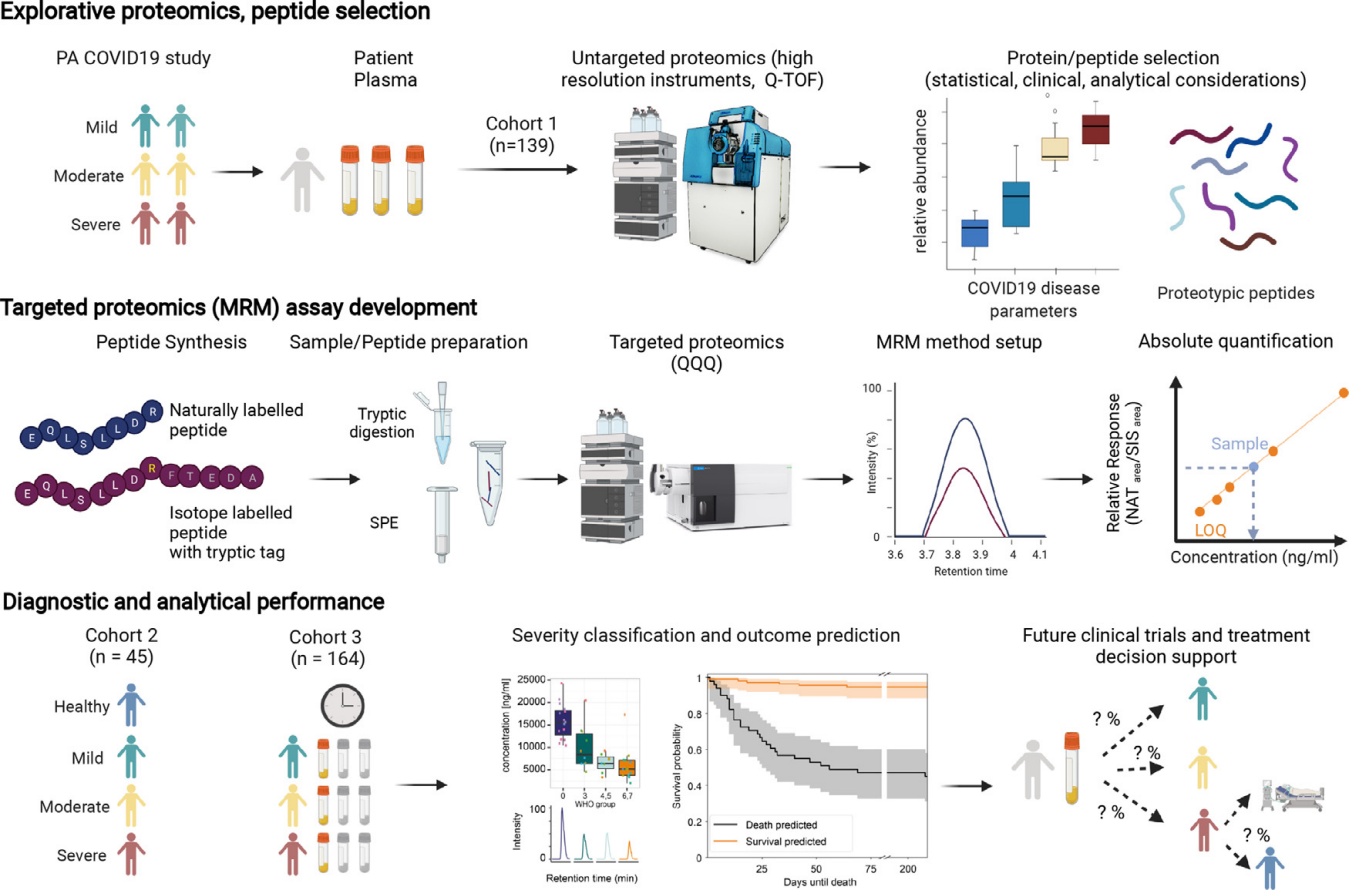
**Schematic overview from peptide selection, to development and application of a COVID-19 biomarker panel.** Top panel: Selection of 50 peptides derived from 30 plasma proteins as measured by discovery proteomics in a research setting^13^^,^^24^ in the PA-COVID19 study cohort. Included proteins/peptides were selected accounting for their performance in the exploratory cohort, clinical, and analytical parameters. Middle panel: To generate an assay suitable for routine clinical laboratories, we established a targeted LC-MRM assay for conventional triple-quadrupole mass spectrometers, running reversed phase chromatography, at a high flow-rate. The assay was optimised using synthetic peptides, and allows for absolute quantification using stable isotope labelled internal standards (‘AQUA peptides’^48^) with a short tryptic tag, to account for the sample preparation (tryptic digest) efficiency. Bottom panel: The assay was applied to two observational cohorts: a well balanced second (‘1st wave’) COVID-19 cohort,^25^ with samples being measured in two laboratories, and a larger longitudinal cohort (‘2nd wave’), with patients treated at the Charité Hospital, a national medical reference centre.

**Figure 2:**
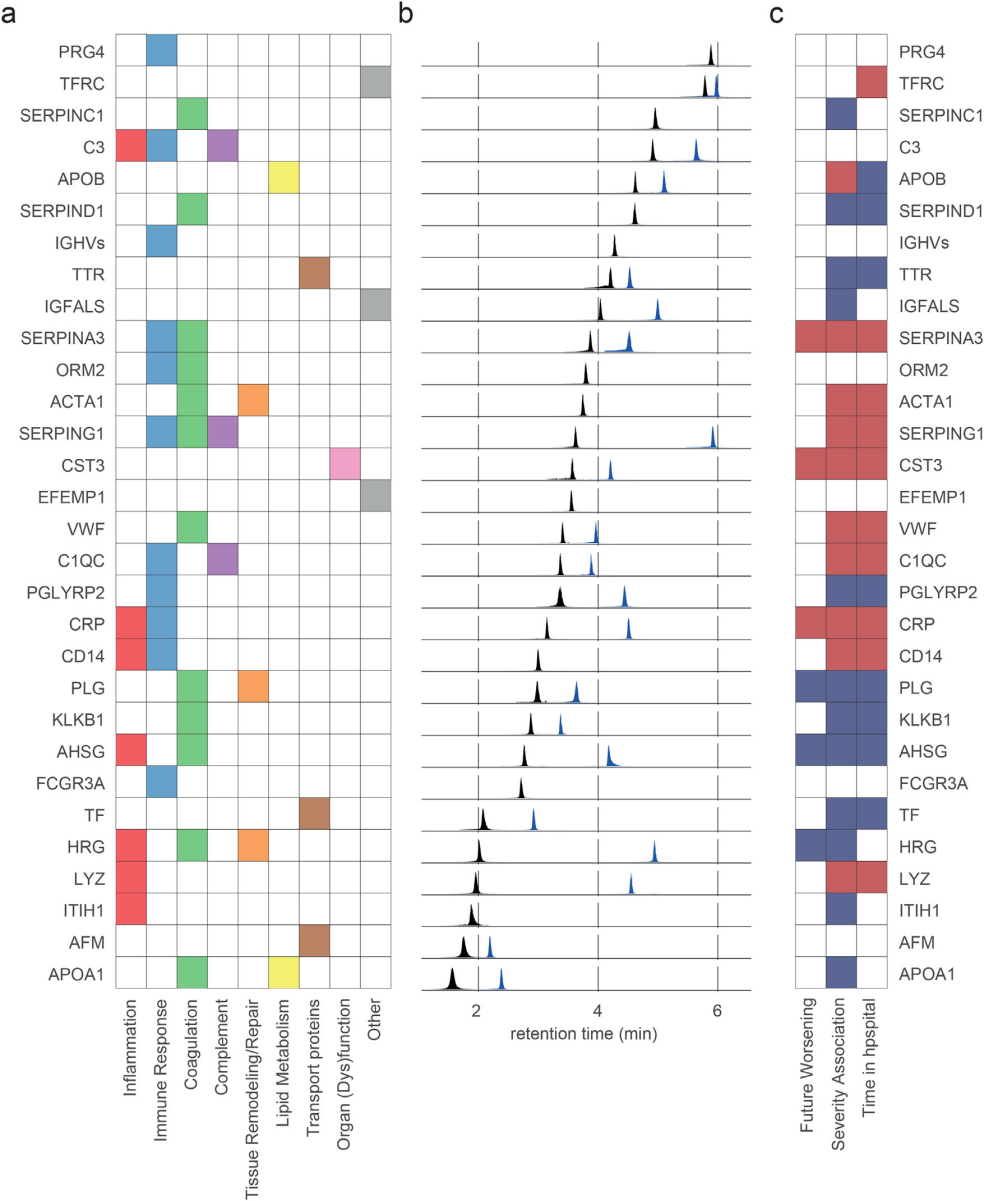
**Selected peptides/proteins analysed by liquid chromatography, multiple reaction monitoring. a)** Analysed proteins and their associated COVID-19-pathology-related processes as curated from literature. **b)** Extracted ion chromatograms (EIC) highlighting the chromatographic spread of the applied MRM transitions selected as quantifiers for the indicated marker peptides. Each EIC was normalised to the maximum intensity of the respective peptide. Note that the majority of proteins are captured by two peptides (arbitrarily coloured as blue, black). **c)** Proteins selected for the panel assay are associated with COVID-19 disease parameters, including severity and progression, and link to COVID-19 related processes. Coloured tiles indicate significant associations, with red/blue highlighting that the respective protein is up- or down-regulated in COVID-19 infected individuals in discovery proteomic data of cohort 1.^13^ For data on individual peptides, see Supplementary Figures 2-4.

50 peptides that corresponded to 30 plasma proteins (Supplementary Table 8) were selected from shotgun plasma proteomics data recorded on a deeply phenotyped cohort of patients with COVID-19 (Figure 1, PA-COVID-19 study cohort, N=139 inpatients, for which 687 plasma proteomes were measured in time series).^13^^,^^30^ Target biomarkers were identified in a ranking exercise that focused on plasma proteins that are (i) prognostic for the remaining time in hospital for inpatients as a treatment-insensitive proxy for COVID-19 severity; ii) differ between treatment escalation levels, expressed on the WHO ordinal scale^37^; or (iii) are prognostic of future worsening, i.e. the progression to a higher WHO severity grade^13^ of inpatients who were admitted with a milder disease which then deteriorated.^13^^,^^16^^,^^21^^,^^24^^,^^38^ Part of the selected proteins are already monitored clinically in this or other indications, including SERPINC1 = Antithrombin-III,^13^^,^^39^ C3 = Complement C3,^13^ APOB = Apolipoprotein B,^13^ SERPING1 = C1-inhibitor,^13^^,^^38^ CST3 = cystatin-C,^13^ VWF = von Willebrand factor,^9^^,^^13^^,^^38^ CRP = C-reactive protein,^9^^,^^13^^,^^24^^,^^38^ PLG = plasminogen,^9^^,^^13^ KLKB1 = plasma kallikrein,^9^^,^^13^ LYZ = lysozyme,^13^ and APOA1 = Apolipoprotein A^13^^,^^24^ (Figure 2a). For new markers, a selection criterion was that they were deposited in MRMAssayDB, as an indicator of being chosen as markers also in other settings.^40^ Further, we evaluated technical parameters such as suitability for synthesis (as evaluated by the Thermo Peptide Analysing Tool), a mass range applicable for a MRM assay, and distribution over the chromatographic gradient (Figure 2b).

From the final selected panel, 18/30 proteins are associated with remaining time in hospital, 22/30 with disease severity, and 6/30 are prognostic of future worsening (Figure 2c). Six additional peptides included were prognostic for remaining time in hospital (PRG4, C3, EFEMP1, ORM2, FCGR3A, AFM, IGHVs). For data and statistics, see Supplementary Figures 2–4.

Lastly, we tested whether the selected surrogate peptides were unique in a Uniprot BLAST and manual human proteome FASTA text file search. This was true for 45/50 peptides. The remaining 5 peptides (CQSWSSMTPHR, EITALAPSTMK, WEMPFDPQDTHQSR, DSGSYFCR, ASDTAMYYCAR) were shared across closely related protein isoforms (Supplementary Table 8) but were retained in the panel composition as they fulfilled the selection criteria.

### Establishment and analytical validation of a MRM-based, targeted COVID-19 biomarker assay

For each selected peptide, 2 standards were synthesised: 1) with a natural isotope distribution (‘native’), and 2) with a C-terminal SIL amino acid to act as an ISTD, which contained a short tryptic tag to account for the digestion efficiency (Supplementary Table 8). In order to establish the assay for routine settings, we chose analytical flow rate reversed-phase chromatography. The native peptides were employed to optimise LC-MS/MS data acquisition method and quality of the Q1/Q3 (MRM) transitions (257 overall) on a 6495C (Agilent) system. The eluted peptides were well distributed along a 8.6-minute linear gradient and were quantified using a scheduled MRM method (Figure 2b).

We then tested intra- and inter-batch precision, linearity, LOQ, accuracy, and potential matrix effects. We calculated the coefficient of variation (CV) for the triplicate of independently prepared calibration curves. These were constructed from serial dilutions of native peptide standards in BSA (40 mg/ml), measured in technical pentuplicates (i.e. total of N=15) on the LC-MS/MS system. We used BSA as a surrogate matrix to test the analytical performance in the absence of the endogenous plasma peptides^41^ and achieved a median intra-batch CV of 2.6% and median inter-batch CV of 10.9% across low (LLOQ), medium ((LLOQ+ULOQ)/2), and high (ULOQ) concentration points (Supplementary Table 9).

Additionally, we determined the limits of quantification (LOQ). 37 peptides exceeded the inter-batch CV criteria at LLOQ of ≤ 20%, and 10 additional peptides could be quantified with an expanded LLOQ CV cutoff of ≤40%. Calibration curves for 47 peptides revealed a median LLOQ of 143.26 ng/ml, and typically allowed quantification over 3–4 orders of magnitude on a linear dynamic range (R² > 0.99, Supplementary Table 9).

As analytical technologies are sensitive to matrix effects,^42^ we evaluated parallelism in the surrogate BSA matrix compared to human plasma. We compared the slopes obtained from calibration samples measured in a commercial human plasma sample with those measured in the surrogate matrix. 39/47 quantified peptide biomarkers showed no statistically significant matrix effect (P > 0.05). For the 8 peptides that differed significantly a matrix factor (slope plasma/slope BSA x 100%) was calculated and reported (Supplementary Table 9).

To test if peptide quantities from actual patient samples would be covered within the linear range of the calibration curves, we performed absolute quantification in plasma samples obtained from patients with COVID-19 (pooled COVID-19-patient samples of different WHO treatment escalation grades; see Methods). Peptide concentrations were covered within the determined linear range of the assay of pooled samples from WHO severity grade 3-7 (Supplementary Table 10).

### The assay reports disease severity in an early pandemic cohort

Next, we assessed how the absolute concentration of the quantified biomarkers changed as a function of the COVID-19 treatment escalation level, a proxy for disease severity.^37^ We applied the panel assay on plasma samples obtained from a deeply characterised, early-pandemic COVID-19 cohort, hospitalised between March 1 and 26, 2020 (‘Cohort 2’, n=45, Supplementary Table 1).^25^ This cohort was suited for this validation step, as it was balanced, with patients with mild to severe COVID-19, and included healthy controls.^25^^,^^30^ Furthermore, the cohort was sampled as citrate plasma, in difference to the exploratory cohorts in our previous studies to identify the biomarkers which were sampled as EDTA plasma. This test was hence also indicative if the biomarkers would allow stable conclusions across alternative sample matrices.

**Figure 3:**
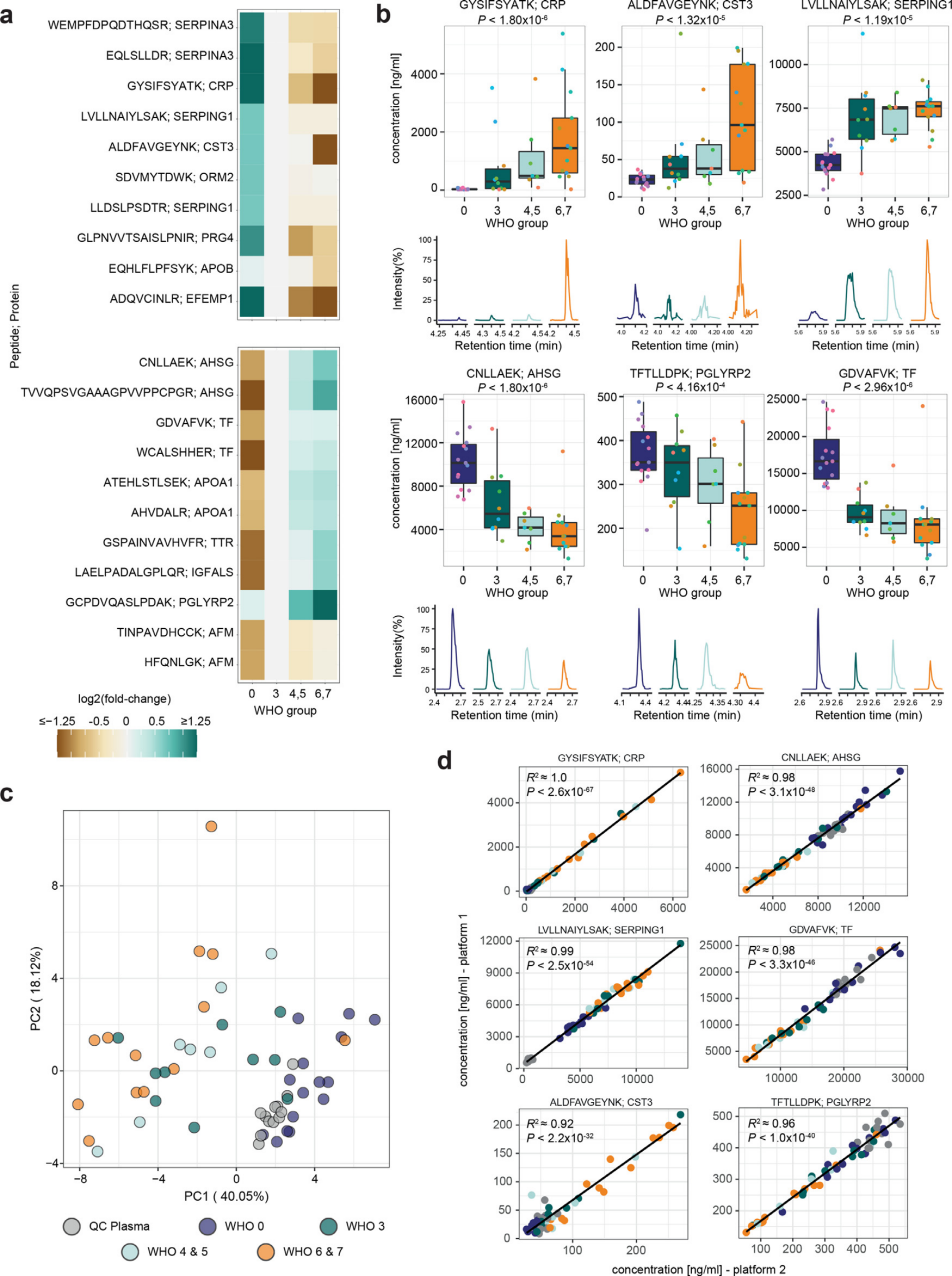
**The protein biomarker assay reproducibly reports disease severity in a COVID-19 cohort. a)** The established assay was applied to citrate plasma samples, collected for a balanced COVID-19 cohort studied during the first wave of the pandemic^24^^,^^25^^,^^30^ (‘Cohort 2’) consisting of healthy volunteers (n=15, WHO 0), COVID-19 affected individuals requiring hospitalisation but no oxygen therapy (n=10 (WHO3), COVID-19 affected individuals requiring hospitalisation and non-invasive oxygen therapy (n=4, WHO4; n=3 WHO5), and severely affected hospitalised individuals requiring mechanical ventilation (n=3 (WHO6), n=10 (WHO7) as well as QC plasma samples (n=12). Peptides with a significant concentration change (up- (top panel) and down-regulated (bottom panel)) distinguish healthy from infected individuals, as well as mild from severe forms of the disease. Heatmap displays the log_2_ fold-change of the indicated peptide to its median concentration in patients with a severity score of WHO3. Only significant peptides (adjusted P < 0.05) with an arbitrary fold-change difference <1/1.5 or >1.5 are shown, and log_2_ fold-changes <-1.25 or >1.25 are indicated by the same respective colour. For additional information, see Supplementary Figure 5. **b)** Visualisation of the response to COVID-19 based on selected peptides indicating different COVID-19 severity trends (changing with severity expressed according to the WHO ordinal scale (left, middle panel), and differentiating healthy from COVID-19 infected individuals (right panel)). Boxplots display the absolute concentration of selected peptides in patients in different severity groups as explained in (a). The extracted ion chromatograms (EIC) display the response of representative samples of individuals classified according to the treatment escalation WHO=0, WHO=3, WHO=5 and WHO=7. **c)** Unsupervised clustering by principal component analysis (PCA) based on the absolute concentration of 39 quantified peptides clusters patients with COVID-19 by severity. The peptide ADQVCINLR contained missing values and was omitted. **d)** Analytical reproducibility of the assay in two laboratories, running two different LC-MS/MS platforms, with independently optimised MRM transitions. Shown are linear correlations (pearson correlation) between absolute peptide concentrations. Selected peptides and colour code like in (c). For boxplots, the median is marked by a solid line, hinges mark the 25th and 75th percentiles, and whiskers show all values that, at maximum, fall within 1.5 times the interquartile range.

Samples were prepared using a semi-automated platform designed for precision.^24^ 40/50 peptides were reliably quantified in the patient citrate plasma (Supplementary Figure 5, Supplementary Data 1). 32 peptides changed with disease severity, i.e. from uninfected (WHO 0) to mildly (WHO 3), moderately (WHO 4, 5) and severely (WHO 6, 7) affected patients (Figure 3a, Supplementary Figure 5, P < 0.05). Most of the chosen markers change in abundance between healthy and COVID-19-infected individuals, and further follow their respective trend with increasing treatment escalation level, such as peptides derived from the acute-phase proteins CRP and AHSG, or the innate-immune-response protein PGLYRP2 (Figure 3a, Figure 3b). Some of the peptides give a signal during specific disease state transitions, i.e. they differ between an infected and uninfected individual (such as peptides from the complement-related protein SERPING1 or the iron-binding protein TF (Figure 3a, Figure 3b)), or change the most during the most severe treatment escalations of COVID-19 (such as the kidney and inflammation marker CST3 (Figure 3a, Figure 3b)). As a profile, the protein marker quantities did classify the patients according to the treatment escalation score (Figure 3c).

### Analytical cross-platform and cross-laboratory validation

The application of different instruments and sample matrices can lead to differences in the quantification of peptides. To evaluate the assay transferability, samples from Cohort 2 were measured on both the 6495C (Agilent) and the 7500 (SCIEX) LC-MS/MS platforms, in different laboratories. For 33/40 selected peptides, we obtained a clear cross-laboratory/cross-instrument correlation between the concentration measured in respective COVID-19-patient samples (Figure 3d, Supplementary Figure 6). One peptide (ESDTSYVSLK) suffered from one outlier, but otherwise had a good correlation on both platforms (Supplementary Figure 6). The remaining (six) peptides were close to the detection limit in the citrate plasma sample matrix, which caused limited correlation (R^2^ < 0.6) between both platforms. Further, on a subset of peptides we observed correlation but different absolute values, pointing to differences in calibration.

### Severity stratification and outcome prognosis in a longitudinal COVID-19 cohort

We next studied a larger, longitudinal cohort of the second wave of the pandemic, hospitalised between April 4 and November 19, 2020, (“Cohort 3”)). Of 164 inpatients, 23 (14.0%) remained stable without the need for supplemental oxygen throughout their hospital stay, 80 (48.8%) required supplemental low- or high-flow oxygen, and 61 (37.2%) required invasive mechanical ventilation and, in all but 3 cases, additional organ support. Thirty-four (20.7%) patients died, including 5 with do not intubate/do not resuscitate orders in place (Table 1). This cohort was selected because the large number of samples (n=548 samples from 164 patients, Supplementary Data 2, Supplementary Table 11,) i) adds information about the technical stability of the assay and increases the statistical power to evaluate the results, ii) allowed us to assess a potential prognostic value of the assay and iii) to evaluate its applicability after dexamethasone became standard of care for patients requiring supplemental oxygen.

**Figure 4:**
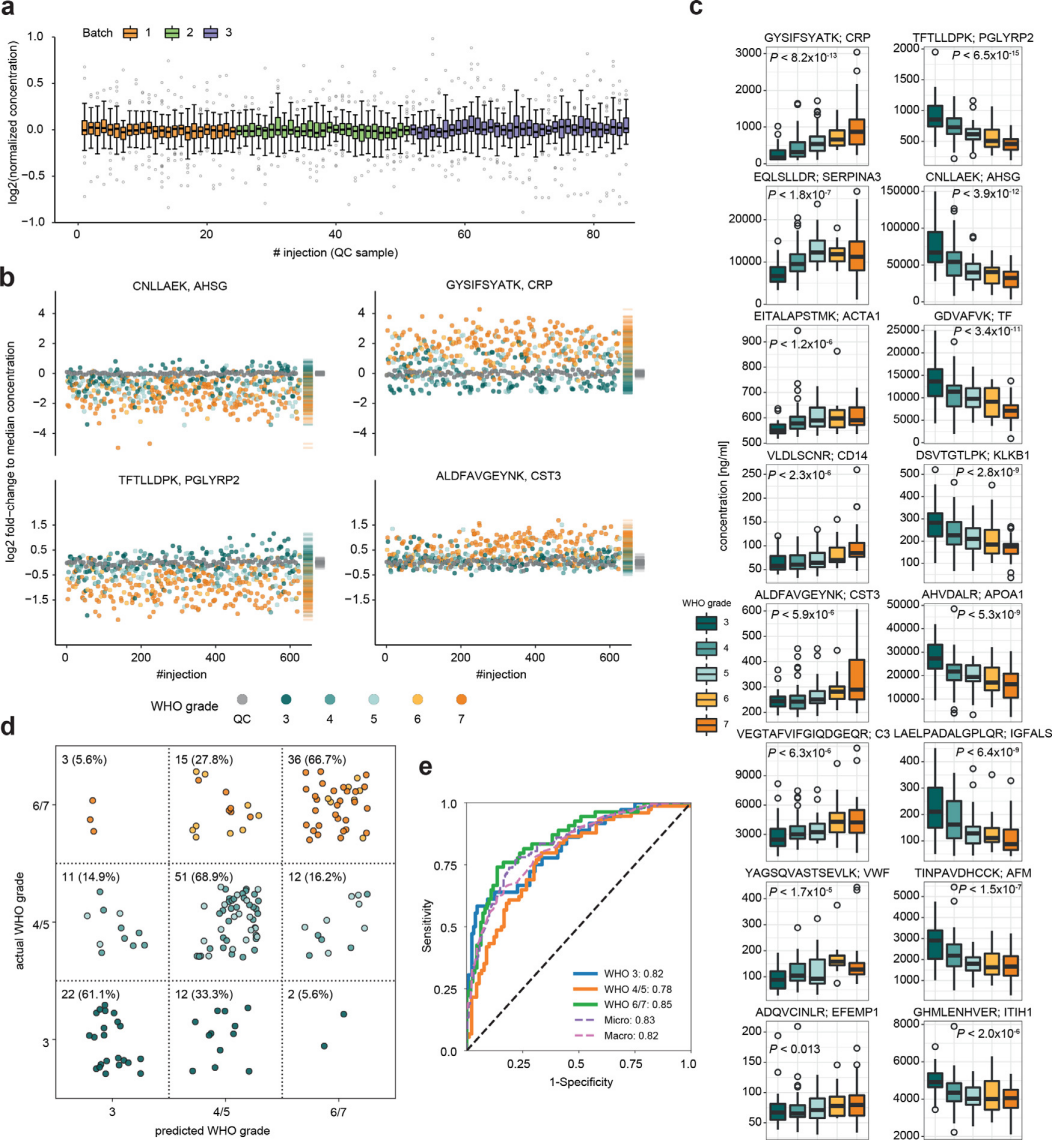
**Diagnostic and analytical performance of the assay on a COVID-19 inpatient cohort treated during the 2nd wave of the pandemic. a)** Quantitative performance (signal stability), during the measurement of 548 plasma proteome samples of patient Cohort 3 evaluated based on n=85 QC samples (coloured in grey, pool of COVID-19 samples as described in^24^) injected throughout the acquisition. Shown are the log_2_ fold-change of the absolute concentration for each of the 48 quantified peptides normalised to the median of the QC samples for the respective peptide. **b)** Peptide log_2_ absolute concentration fold change of two selected down- (AHSG, PGLYRP2) and two up-regulated (CRP, CST3) proteins for all samples acquired for the cohort described in (a). QC samples are shown in grey, all other samples are coloured according to the corresponding COVID-19 WHO treatment escalation score; rug plots on the right side of each peptide indicate the respective distributions. **c)** The 8 most significantly upregulated (left panel) and down-regulated (right panel) peptides indicative of COVID-19 disease severity, expressed as the treatment level according to the WHO scale. For illustration purposes, only one peptide per protein is displayed and one outlier sample in peptide EITALAPSMK was removed. The quantities of all quantified peptides are illustrated in Supplementary Figure 7. **d)** Confusion-matrix-like representation of the outcome of a multi-class classification model (SVM-based) trained to differentiate three WHO severity groups: grade 3, grades 4/5, and grades 6/7. Predictions were done on withheld samples that were not used for training the models (accuracy = 0.665, balanced accuracy = 0.656). The percentage denotes how many samples within each WHO severity group are assigned to each square. The positions of the points within each square were chosen randomly. Colour scheme according to (c). **e)** ROC-curves for the prediction of the WHO severity group from the first time point measured for every patient (grade 3, blue; grades 4/5, orange; grades 6/7, green). Dotted lines denote the micro (magenta) and macro average (pink) ROC-curves. Data shown and analysed in c)-d) are from the first time-point obtained for each individual; n=36 (WHO 3), n=47 (WHO 4), n=27 (WHO 5), n=16 (WHO 6), n=38 (WHO7). For boxplots, the median is marked by a solid line, hinges mark the 25th and 75th percentiles, and whiskers show all values that, at maximum, fall within 1.5 times the interquartile range.

Reassuringly, despite the large number of samples acquired, split over three batches and measured over 10 days, and despite the different matrix (EDTA plasma), technical variation was low (Figure 4a). Peptides which were characteristic for disease severity in Cohort 2 (Figure 2b) also differentiated WHO grades in Cohort 3. (Figure 4b). Because they are of the highest practical value and because they are the furthest apart from outcome, we continued with the earliest sample of each patient. Of the 48 peptides quantified in the EDTA matrix, 34 had a significantly different trend between treatment escalation level (WHO3 to WHO7), with 12 peptides significantly increasing and 22 decreasing in concentration (Figure 4c, Supplementary Figure 7). As in Cohort 2, some markers indicated specific disease transitions, while others gradually changed with severity. For instance, CRP, CST3, or CD14 were increased in very severe forms of the disease (WHO7) (Figure 4c).

Next, we tested if the necessary treatment level could be predicted from the first available sample. We constructed a support vector machine (SVM) trained to differentiate between three different treatment groups on the basis of the severity markers: WHO3 (mild COVID-19, hospitalised, but no supplemental oxygen necessary), WHO4/5 (moderate COVID-19, hospitalised, supplemental low- or high-flow oxygen necessary), and WHO6/7 (severe COVID-19, hospitalised, intensive care and invasive mechanical ventilation necessary). The data was split in a training and a validation set in a cross-validated manner. The model predicted the WHO grades in the validation set from the peptide biomarker data. For most patients, the predicted WHO grade was in agreement with the actual treatment escalation (Figure 4d).

**Figure 5:**
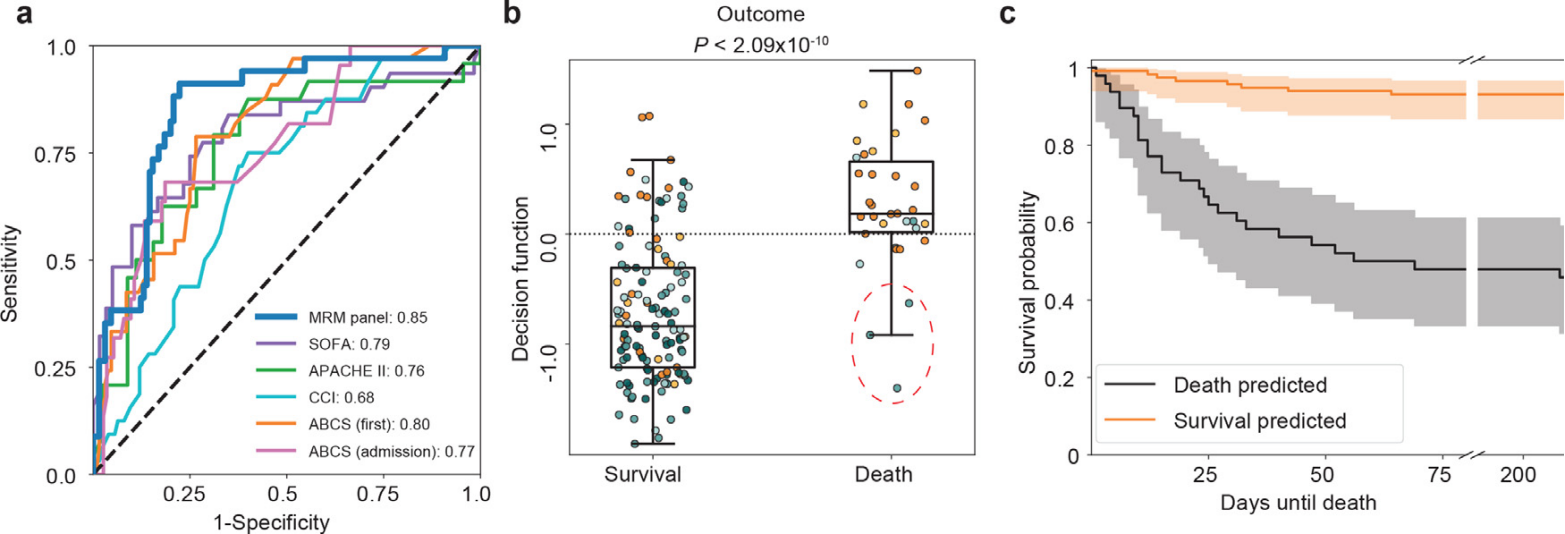
**COVID-19 outcome prognosis. a**) Receiver operating characteristic (ROC) curve for the prediction of survival and non-survival, from a single plasma sample (first sample measured for every patient, n=164) using an SVM-classifier. The blue curve denotes the model trained and benchmarked on measured proteomic data. The other curves denote models based on single severity scores (Sequential Organ Failure Assessment score (SOFA, purple, n=91), Acute Physiology And Chronic Health Evaluation (APACHE II, green, n=69), Charlson Comorbidity Index (CCI, cyan, n=157), and Age, Biomarkers, Clinical history, Sex score (ABCS, pink (admission, n=135) and orange (first sample, n=161)). **b)** Boxplot of the decision function of the SVM for every patient sorted according to the outcome and coloured with respect to the WHO grade at the day the sample was taken. Colour scheme according to Figure 4c. The red dashed circle indicates three patients with a high chance of predicted survival who died. These patients all were WHO4 patients with ‘do not intubate (DNI)’ orders in place due to other medical conditions. Therapy was therefore not escalated to invasive ventilation. The MRM assay classified those patients as milder COVID-19 cases, compared to other non-survivors. The cut point for the binary metrics is highlighted. Sensitivity = 0.765, (26/34); Specificity = 0.831 (108/130). Indicated *P* value reports differences between decision function distributions (survival vs. death) as calculated with a Mann-Whitney U rank test. **c)** Kaplan-Meier estimate of the survival function for survival predicted cases (orange) or non-survival predicted cases (black) with confidence interval (alpha=0.05). Patient survival data for each timepoint is provided in Supplementary Tables 13-15. All predictions were done on withheld samples that were not used for training the models. For boxplots, the median is marked by a solid line, hinges mark the 25th and 75th percentiles, and whiskers show all values that, at maximum, fall within 1.5 times the interquartile range.

An important clinical need for a COVID-19 assay is to be prognostic of outcomes.^43^ An SVM was trained on data obtained from the earliest sample, in a cross-validated manner, to differentiate patients who later survived COVID-19 from patients with a fatal outcome (n=164, of which 130 survived (controls) and 34 died (cases)) (Figure 5). The trained outcome predictor correctly classified 81.7% of the patients (sensitivity = 0.765, (26/34) specificity = 0.831 (108/130), AUROC = 0.855) that were withheld while training the model (Figure 5a, b). Additionally, a decision curve analysis (Supplementary Figure 8) shows a higher net benefit over a long range of threshold probabilities for the trained SVM classifier compared to the reference strategies. To exclude that the predicting capabilities are limited to the method, two other predictors (logistic regression and extra-trees) using the same setup were evaluated (Supplementary Figures 9 and 10). Reassuringly, these predictors (logistic regression: sensitivity = 0.735, specificity = 0.854, AUROC = 0.848; extra-trees: sensitivity = 0.706, specificity = 0.815, AUROC = 0.836) show a comparable performance. To evaluate how well the SVM predictor performs compared to the clinical scores, we determined SOFA, APACHE II, and CCI scores as well as the COVID-19-specific ABCS score. SOFA, APACHE II, and ABCS, which are directly linked to the patient's disease severity, performed best among the four scores tested. Nonetheless, the MRM biomarker assay outperformed all other scores, as indicated by ROC analysis (Figure 5a, Supplementary Table 12.)

## Discussion

The COVID-19 crisis has reminded us that novel infectious diseases can quickly challenge health systems on a global scale. Although COVID-19 has meanwhile become a well-studied disease, there remains unmet clinical need for personalised tests that can support clinical decision making and guide development of novel treatments. The most important decision points for the clinical management of COVID-19 are i) at admission, posing the question whether a patient needs inpatient care, ii) at regular inpatient wards, when decisions have to be made whether more intense monitoring and respiratory support (i.e. intensive care) are necessary during clinical deterioration, which is common during the second week of COVID-19 symptoms, and iii) at intensive care units, where decisions about additional organ replacement treatment such as extracorporeal membrane oxygenation need to be made.

Several investigations have highlighted the classification and prognostic value of plasma proteomes in COVID-19.^9^^,^^10^^,^^16^^,^^21^, ^22^, ^23^ Discovery proteomic technologies are difficult to implement in a clinical routine. However, one can translate a proteomics result through the selection and validation of biomarker panels; and technologies, such as triple quadrupole mass spectrometers coupled to analytical flow rate chromatography are routinely used in clinical and regulated laboratories.^44^^,^^45^ Indeed, their application in clinical proteomics is desirable as LC-MRM i) provides high sensitivity and specificity, ii) allows the inclusion of internal standards for higher precision and control over potential matrix effects,^46^^,^^47^ iii) facilitates absolute quantification, enabling cross-platform transferability,^48^^,^^49^ and iv) covers a large dynamic range.^50^ This in turn enables the comparison of biomarkers with large abundance differences within one run, thus facilitating multiplexing of many biomarkers and downstream processing and statistical analysis.

To simplify the transition from discovery to applied proteomics, we have recently introduced a proteomics platform that uses analytical flow rate chromatography already at the discovery stage.^13^^,^^24^ Herein, we used discovery proteomic data recorded with this platform, to design a multi-protein biomarker panel for severity stratification in COVID-19. The developed biomarker panel includes 50 peptides derived from 30 plasma proteins. These peptides were selected both for their association with COVID-19, for instance the innate immune response, the coagulation system, or the complement cascade,^13^^,^^24^^,^^25^ and for technical reasons, for instance the distribution over the chromatographic gradient. The assay is robust and can be ported to different platforms and matrices, in some of which not all peptides will be quantifiable, as to be determined by a set of quality controls. In this study, we established the assay on two different routine-laboratory-compatible LC-MRM platforms. We demonstrate sensitivity, accuracy, precision, as well as overall reproducibility on both platforms.

We confirm in two temporally separated COVID-19-patient cohorts that the panel assay captures disease severity of SARS-CoV-2-infected individuals and discriminates the necessary treatment levels. This is particularly reassuring as the standard of care changed to include dexamethasone for treatment of respiratory failure in the outcome validation cohort (Cohort 3), following publication of the results of the recovery trial.^51^ We also tested the prognostic value of the panel and found that it outperformed four clinical risk-assessment metrics, ABCS, CCI, SOFA, and APACHE II, in predicting the survival of COVID-19 inpatients, as revealed by a ROC analysis. Thus, the panel assay could be used to assess the current state of the patient, help monitor novel treatments, or stratify patients based on their responsiveness to novel therapeutic interventions. Furthermore, the assay can be employed to predict the future course of COVID-19, as exemplified by the prediction of disease outcome weeks into the future.

While the value of quantitative proteomic measurements for disease stratification was established in different cohorts, further validation of the outcome prediction will be required in an independent cohort. Similarly, not all eventualities of a complex disease such as COVID-19 are covered with this assay. Additional data to build improved models, and ideally prospective studies, will be of high value, especially to refine predictive models with regard to new SARS-CoV-2 variants and the additional treatment options such as JAK inhibitors. Similarly, it will be important to assess the performance of the assay in other populations, including ambulatory, asymptomatic, and patients affected by other infectious diseases. Another important criterion to be determined is the time point where the signature from this assay is most predictive of outcome. For instance, we recently described that the early spike of the inflammatory response and its gradual decrease is prognostic for patients surviving the disease.^13^^,^^43^

Proteomics can be prognostic of outcome in patients with similar disease severity, e.g. among severely affected patients^43^ that are difficult to distinguish by clinical parameters. This means that survival prognosis using targeted proteomics could be improved beyond what was shown in this study for ‘within-severity-group’ prognosis, if biomarker panels were selected specifically for stratification within the respective COVID-19 severity group. Indeed, particularly within the group of severely affected individuals, some patients were predicted incorrectly, i.e. survived despite being predicted as non-survivors, or vice versa (Figure 5b). We assessed on a patient-by-patient basis whether there are medical reasons that could explain wrong predictions. Plotting outcome with respect to time until death (Kaplan–Meier survival analysis, Figure 5c) denotes no clear tendency for the correct and false predictions. However, we noted that the three samples with the smallest decision function across wrongly predicted patients with fatal outcomes belonged to WHO4 patients that had DNI (‘do not intubate’) orders in place (denoted with a red circle in Figure 5b). It is hence plausible that the assay correctly identified a milder form of COVID-19 in these three individuals; i.e. that without a strong comorbidity or a DNI order in place, these might have had a good chance to survive COVID-19. Similarly, we recently reported two cases where the proteomic signatures of patients correctly distinguished an influenza B from a SARS-CoV-2 infection, and that highlighted a patient that had to undergo chemotherapeutic cancer treatment just days before a SARS-CoV-2 infection.^24^ Thus, protein signatures could in principle distinguish different (respiratory) infections or comorbidities, while additional research will be required to establish this for the presented protein panel.

COVID-19 will remain a central public health issue for the foreseeable future as new variants of concern with capacity to evade vaccine-induced immunity continue to emerge.^52^ The underpinning targeted proteomics platform supports rapid iteration of the panel composition in case additional prognostic biomarkers are discovered. Taken together, this peptide panel and the underlying analytical platform hold potential to support a broader, continuous pandemic response in addition to their utility in hospitalised patient cohorts, which we demonstrate in the present study.

## Contributors

MM, ES, JH, MR conceptualised and supervised the study. ZW, AC, OL, ST, JH, ES formally analysed and curated the data. FK, MR, ES acquired funding. PTL, EH, LES and FK acquired clinical data, ZW, JH acquired LC-MS/MS data (on Agilent 6495), DB, CSL, RS acquired LC-MS/MS data (on SCIEX 7500). DL performed sample preparation. VD selected the peptides. ZW, AC, OL, ES, JH developed methodology. SH, LES, MM, FK, ES, JH and MR administered the project. CM, JZ provided resources. OL developed software. ZW, OL, AC, ES, JH validated the data and methodologies. ZW, OL, MM, JH visualised the results. ZW, OL, PTL, FK, ES, JH, MR wrote the original draft. ZW, OL, PTL, FK, ES, JH, MR revised and edited the manuscript. All authors reviewed the manuscript and had access to the data generated in the study. Authors involved in formal analysis and data curation, validation, and investigation directly worked with and verified the data. F.K., E.S., J.H. and M.R. decided to submit the manuscript.

## Data sharing statement

Participant data of Cohort 3 that collected during the trial is available in (Supplementary Table 11). For other cohorts, respective information is available in previous publications.^13^^,^^24^ Measurement data, as well as custom used code used for modelling / data analysis are available online.^53^

## Declaration of interests

EM Scientific Limited (t/a Inoviv) and Charité – Universitätsmedizin Berlin (Ziyue Wang, Michael Mülleder, Vadim Demichev, Johannes Hartl and Markus Ralser) filed joint patent applications for the protein panel assay described herein - United States Application No: 63/156291, 63/283787 and 17/685756. Leif-Erik Sander received honoraria for lectures from Boehringer Ingelheim, Novartis, Berlin Chemie, GSK, Merck, Novartis, Sanofi. Ernestas Sirka and Adam Cryar are/were employees of EM Scientific Limited (t/a Inoviv). Daniel Blake, Rebekah L Sayers and Catherine S Lane are employees of SCIEX. Christoph Mueller and Johannes Zeiser are employees of Agilent Technologies.

## Appendix Supplementary materials

Image, application 1
