# Supplementary material for: A multiplex protein panel assay for severity prediction and outcome prognosis in patients with COVID-19: An observational multi-cohort study
Source: eClinicalMedicine. 2022 Jun 9;49:101495. doi: 10.1016/j.eclinm.2022.101495 (PMC9181834; doi:10.1016/j.eclinm.2022.101495)
Supplement: Supplementary file 1 [file mmc1.pdf]

Supplementary Information

A multiplex protein panel assay determines disease severity and is prognostic of outcome in patients with COVID-19: An observational multi-cohort study

Contents:

Supplementary Methods	Page 2-4
Supplementary Figures 1-10	Page 5-13
Supplementary Tables 1-15	Page 14 - 40
Supplementary References	Page 41

Supplementary Methods

Reagents

Water was from Merck (LiChrosolv LC-MS grade; Cat# 115333), acetonitrile was from Biosolve (LC-MS grade; Cat# 012078), trypsin (Sequence grade; Cat# V511X) was from Promega, 1,4-Dithiothreitol (DTT; Cat# 6908.2) from Carl-Roth, iodoacetamide (IAA; Bioultra; Cat# I1149) and urea (puriss. P.a., reag. Ph. Eur.; Cat# 33247) were from Sigma-Aldrich, ammonium bicarbonate (Eluent additive for LC-MS; Cat# 40867) and Dimethyl sulfoxide (DMSO; Cat# 41648) were from Fluka, formic acid (LC-MS Grade; Eluent additive for LC-MS; Cat# 85178) was from Thermo Scientific™, bovine serum albumin (BSA; Albumin Bovine Fraction V, Very Low Endotoxin, Fatty Acid-free; Cat# 47299) was from Serva, commercial human plasma samples (Human Source Plasma, LOT# 20CILP1034) was from zenbio.

Liquid chromatography - tandem mass spectrometry

6495C (Agilent) LC-MS/MS method

All clinical samples were analysed on the Agilent 6495C mass spectrometer, coupled to an Agilent 1290 Infinity II UHPLC system. Prior to MS analysis, samples were chromatographically separated on an Agilent InfinityLab Poroshell 120 EC-C18 1.9 µm, 2.1x50 mm column heated to 45 °C and with a flow rate of 800 µl/min. Linear gradients employed were as follows (time, % of mobile phase B): 0 min, 3%; 1 min, 3%; 7.5 min, 35%; 8 min 98%; 8.5 min, 98%; 8.6 min, 3%; 10 min, 3% where mobile phase A & B are 0.1 % formic acid in water and 0.1 % formic acid in acetonitrile respectively.

The 6495C mass spectrometer was controlled by Agilent's MassHunter Workstation software (LC-MS/MS Data Acquisition for 6400 series Triple Quadrupole, Version 10.1) and was operated in positive electrospray ionisation mode with the following parameters: 3500 V capillary voltage (positive), 0 V nozzle voltage (positive), 12 L/min sheath gas flow at a temperature of 280 °C, 17 L/min gas flow at a temperature of 170 °C, 40 psi nebulizer pressure, 166 V fragmentor voltage, 5 V cell accelerator potential. Samples were analysed in dynamic MRM mode with both quadrupoles operated in unit resolution. All other MRM parameters, including monitored transitions and scheduling are provided in the Supplementary Table 2.

7500 (SCIEX) LC-MS/MS method

Samples from cohort 2 were analysed on a SCIEX 7500 mass spectrometer coupled to an ExionLC AD UHPLC system (SCIEX, UK) in addition to the analysis on the Agilent platform. Prior to MS analysis, samples were chromatographically separated on a Phenomenex Luna Omega Polar 3 µm, 100 x 2.1 mm column heated to 40 °C and with a flow rate of 500 µl/min. Linear gradients employed were as follows (time, % of mobile phase B): 0 min, 3%; 0.1 min, 3%; 7.5 min, 30%;8 min 95%; 8.5 min, 95%; 8.6 min, 3%; 10 min, 3% where mobile phase A & B are 0.1% formic acid in water and 0.1% formic acid in acetonitrile respectively.
The 7500 triple quadrupole mass spectrometer was operated in positive electrospray ionisation mode with the following ion source parameters: 1750 V Ionspray voltage, 40 psi curtain gas, 40 psi Ion source gas 1, 70 psi ion source gas 2 and 500 °C temperature. Samples were analysed in Scheduled MRM mode with both quadrupoles operated in unit resolution. All other MRM parameters, including monitored transitions and scheduling are provided in the Supplementary Table 3.

Data processing and calibration

For determination of absolute concentrations, calibrations were performed as outlined in the main methods section of the manuscript. Of note, SIL internal standards for 5 corresponding native peptides could not be detected on the 6495C system. To quantify these native peptides, we used other, closely eluting SIL internal standards in the assay: AADDTWEPFASGK(U-$^{13}C_6$,$^{15}N_2$ was used for ASDTAMYYCAR, GYSIFSYATK(U-$^{13}C_6$,$^{15}N_2$) for GSPAINVAVHVFR and WEMPFDPQDTHQSR, ANRPFLVFIR(U-$^{13}C_6$,$^{15}N_4$) for LAELPADALGPLQR and VSASPLLYTLIEK(U-$^{13}C_6$,$^{15}N_2$) for VEGTAFVIFGIQDGEQR. In addition, due

to low signal intensity of pre-assigned quantifier transitions (transitions with matched precursor and product ions across native and SIL peptides), we chose other transitions with higher signal intensity for 4 SIL peptides, even if they did not match the fragmentation pattern of their respective native peptides.

Statistical analysis

Significance testing of the trend between absolute peptide concentrations and the ordinal classification as provided by the WHO treatment escalation scale (levels as indicated) was performed using Kendall's tau (KT) statistics as implemented in the “EnvStats v2.4.0” R package “kendallTrendTest” function. For cohort 2 the KT statistics was calculated as the trend of absolute peptide concentrations against the following WHO groups: 0, 3, 4, 5, 6, 7; 40 peptides were used for data analysis, without imputation. For cohort 3 the KT statistics was calculated as the trend of absolute peptide concentrations against the following WHO groups: 3, 4, 5, 6, 7; 48 peptides were used for data analysis. For the peptide VSASPLLYTLIEK negative values were present after external calibration, which were replaced by 0. Where indicated, multiple testing correction was performed by controlling for false discovery rate using the Benjamini-Hochberg procedure [1] as provided by the R package “stats v4.1.0” - “p.adjust” function. A full summary of these statistical test results is provided in Supplementary Table 4,5. (Adjusted) P values were considered significant when $P < 0.05$.

The Extra Sum of Square F test to evaluate matrix effects was performed using the R function “var.test” from the “stats v4.1.0” package.

Cross-laboratory/cross-instrument correlation was evaluated by calculating the pearson correlations with the R “stats v4.1.0” package “cor.test” function. Results of the correlation analysis are provided in Supplementary Table 6.

Principal component analysis was performed using the R function “prcomp” from the “stats 4.1.0” package and visualised using “ggplot2 v3.3.5”.

Unless otherwise stated, missing peptide concentrations were not imputed as applied statistical tests can accommodate missing values. Instead, for unsupervised clustering (Fig. 3c), the peptide ADQVCINLR was omitted from analysis due to missing values.

Statistical tests on shotgun plasma proteomics data were performed as described in the respective paper [2]. Briefly, the association of peptides/omics features with remaining time in hospital was tested using Wilcoxon W test, and disease worsening was tested using the Mann-Whitney U test, employing the “wilcox.test” function of the “stats” R package. For the association with disease severity, a KT trend test was performed (see above).

Prediction of WHO grade and disease outcome

Clinical scores (CCI [3], SOFA [4], APACHE II [5], and ABCS [6]) were extracted from the clinical information system or, where missing, manually calculated. CCI and APACHE II were determined at time of admission, while SOFA (ICU patients only) was calculated for time of sampling. ABCS was calculated for admission and time of sampling. For ABCS, up to two missing laboratory values (either lymphocytes, blood urea nitrogen (BUN) or aspartate aminotransferase (ASAT)) were imputed by using the median value of patients within the same maximum WHO severity group. Note that due to imputation of the ABCS score memory leakage between training and test data for the ABCS score models can not be excluded.

For the prediction of the current WHO grade and for the outcome prediction a Support Vector Machine was used as implemented in *scikit-learn 0.23.2* (*sklearn.svm.SVC*) [7] using default parameters (rbf-kernel) and balanced class weights (*class_weight* = ”balanced”). For one peptide (VSASPLLYTLIEK) negative values were present after external calibration. Those values were replaced by the minimal positive value of the respective peptide measured over all samples. Two peptides were removed (ASDTAMYYCAR and LVGGPMDASVEEEGVRR) as they were not reliably quantified leaving 48 peptides for the analysis. For every patient the first sample measured was selected (n = 164). All patients with unknown WHO grade/outcome were neglected. All data were log2-transformed and scaled to 0 mean and 1 variance fitted on the training data (*sklearn.preprocessing.StandardScaler*). The model was trained and validated using a shuffled stratified 10-fold cross-validation (*sklearn.model_selection.StratifiedKFold*) to assure that every split has a comparable case-to-control ratio and that every sample was used in 9 runs for training and in the remaining run for validating the trained model not including this sample. For the multiclass treatment a one-vs.-rest approach was

chosen. For reproducibility the seed was fixed to 1. For models trained on established risk assessments scores, only samples for which the respective score was determined were included in model construction and testing.

Decision function, ROC-Curve, accuracy, sensitivity and specificity were calculated using *scikit-learn 0.23.2* using the default cut-offs. Calibrated Precision and F1 score were calculated according to [8] using pi_0 = 0.5. Differences in the decision function of the SVM for every patient grouped according to outcome was compared using the Mann-Whitney U rank test in SciPy [9] (*scipy.stats.mannwhitneyu*). For the Kaplan-Meier estimate *lifelines 0.26.0* [10] was used. The data were divided in death and survival predicted cases. For the dying patients, the days until death were included in the model. The samples for people who left the hospital alive were censored. The confidence intervals were calculated using Greenwood's Exponential formula as implemented in *lifelines 0.26.0* (*alpha* = 0.05). For the decision curve analysis, probabilities for the SVM were estimated within *scikit-learn* using Platt scaling [11]. Decision curves were calculated using the *R*-package *dcurves 0.2.0* [12–14].

In addition, predictors based on logistic regression and the extra-trees algorithm implemented in *scikit-learn 0.23.2* (*sklearn.linear_model.LogisticRegression* and *sklearn.ensemble.ExtraTreesClassifier*) were evaluated. The same approach as described above was applied. For the logistic regression an l1-penalty term and the SAGA solver were chosen. To obtain convergence the maximum number of iterations was increased (*max_iter* = 10000). For the extra-trees algorithm the data weren't log2-transformed and scaled as this isn't needed for a tree-based classifier. In addition, the maximal depth of the trees was set to 3 (*max_depth* = 3) to compensate for overfitting issues due to limited data set size. Feature importances were extracted from a model trained on all data (n=164) without splitting the data set.

Supplementary Figures

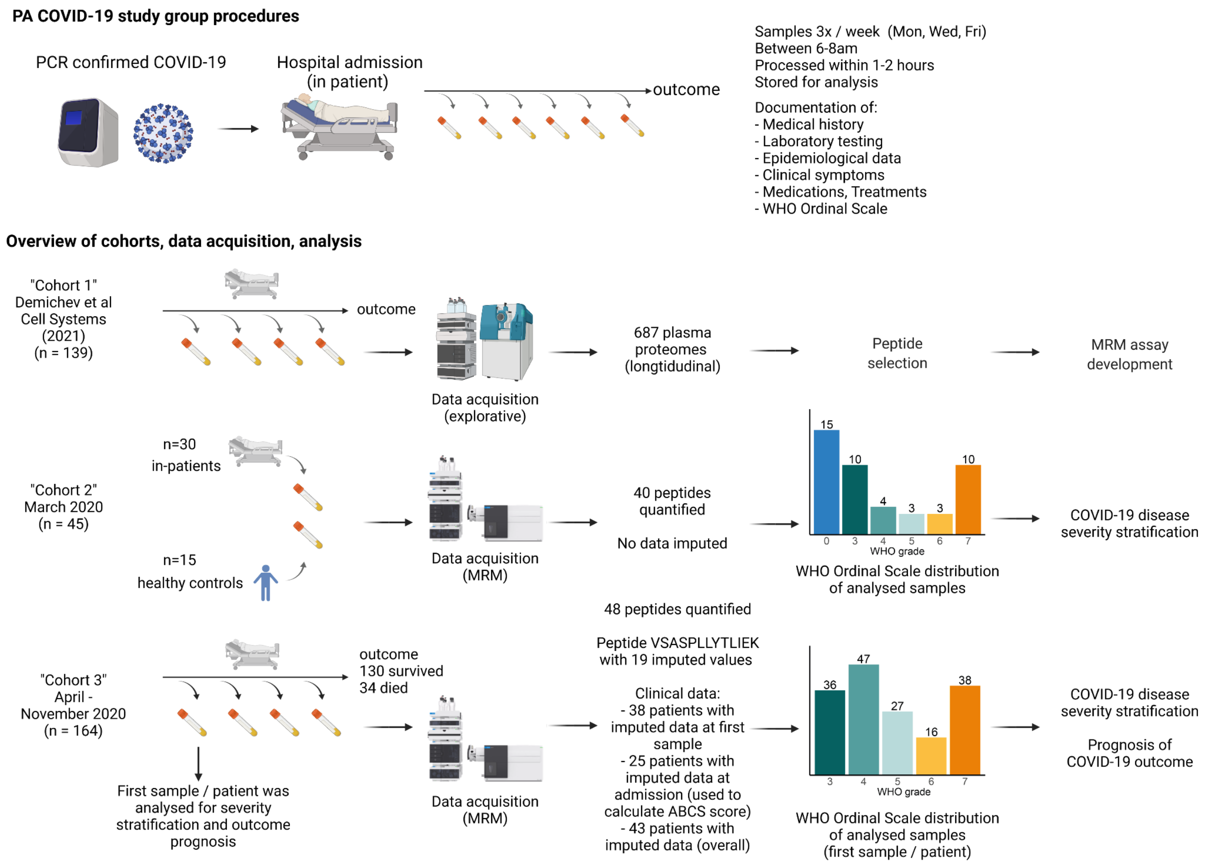

Supplementary Figure 1. Overview of the study design, cohorts, sampling, methodology and data analysis. Top panel: Patient samples were collected as part of an observational cohort study at Charité – Universitätsmedizin Berlin. The study protocol as well as patient characteristics, treatment and outcomes have been described in detail before [15–17]. All in-patients with PCR-confirmed SARS-CoV-2 infection treated at Charité were eligible for inclusion. Bottom panel: Proteins/peptide selection for the MRM panel was guided by data from an explorative proteomics study [2]. The herein developed MRM assay was applied to Cohort 2 to evaluate its performance with respect to capturing disease severity in a '1st wave' COVID-19 cohort [18] which included healthy samples. Furthermore, it was applied to a larger cohort collected during the '2nd wave' of the pandemics - Cohort 3 - to evaluate the ability of the assay to predict COVID-19 severity and be prognostic about disease outcome.

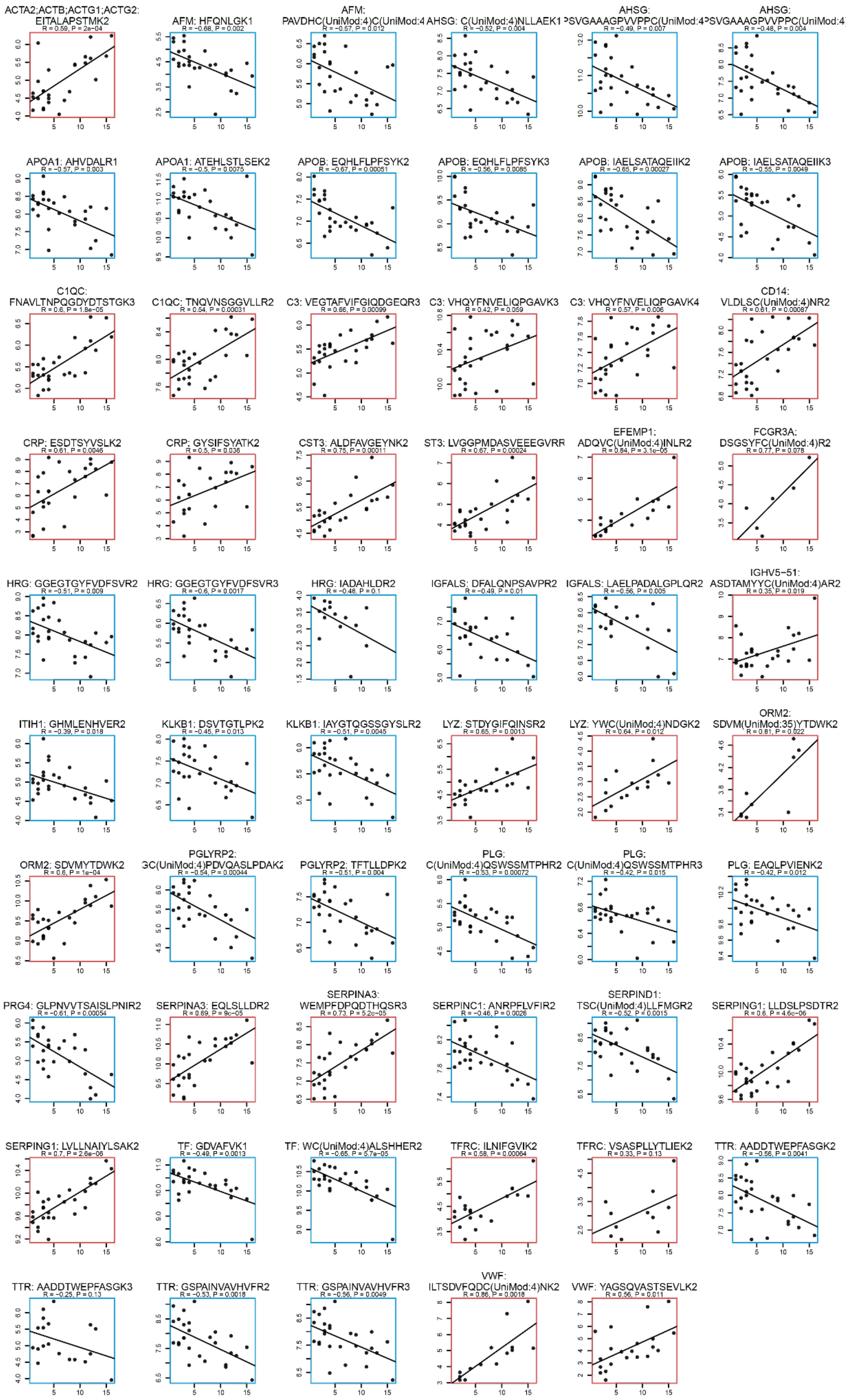

Supplementary Figure 2. Relationship between the quantities of selected precursor ions and the remaining time in hospital for patients that did not require supplemental oxygen. Illustrated are the log2-transformed intensities of precursor ions originating from the MRM panel peptides, recorded at the first time point sampled at WHO grade 3, plotted against the remaining time in hospital. Statistical testing was carried out by including the patient age as covariate, as described previously, and, for visualization, a correction for the impact of age as covariate was performed [2].

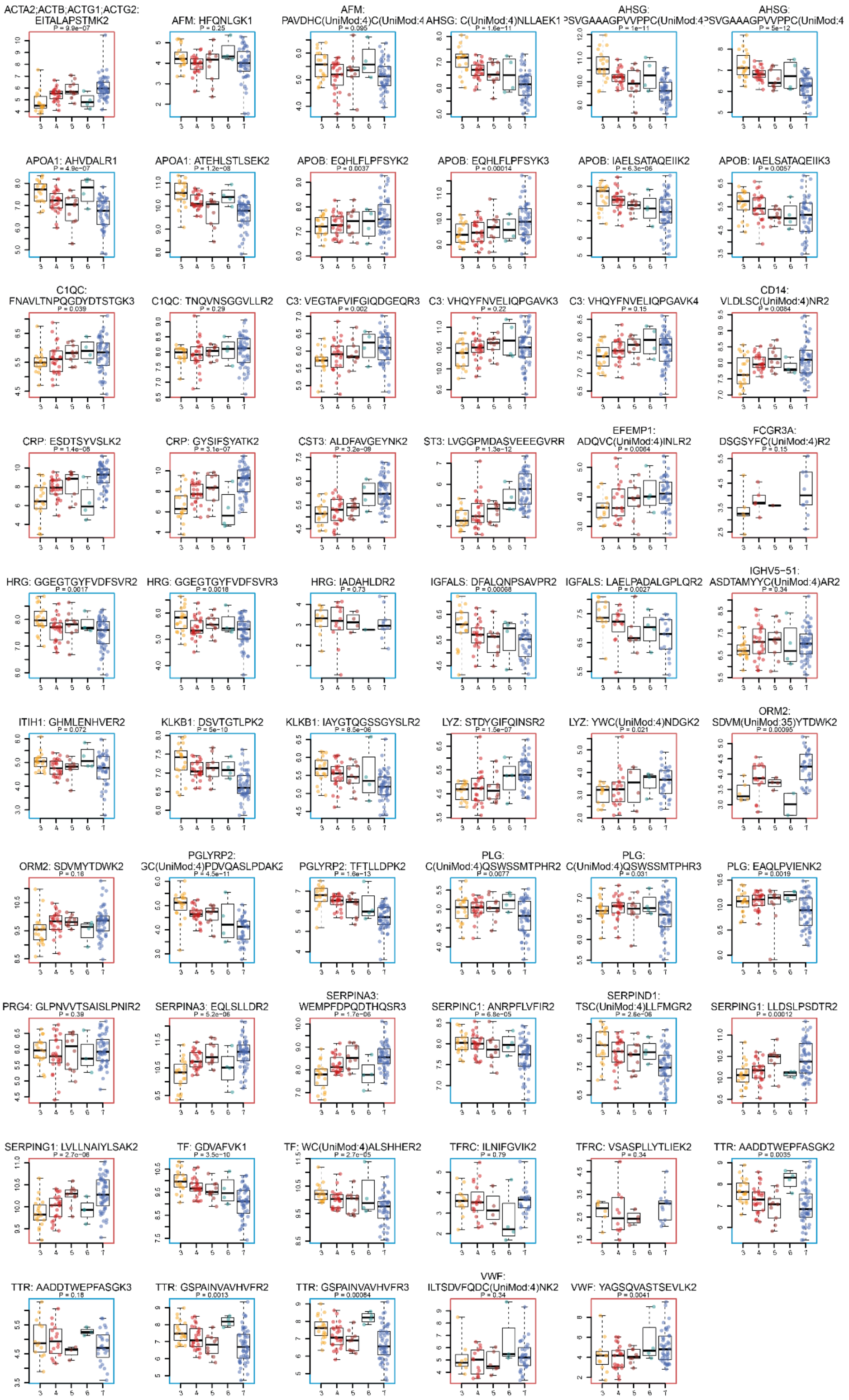

Supplementary Figure 3. Relationship between the quantities of selected precursor ions and the severity grade on the WHO scale. Illustrated are the log2-transformed intensities of precursor ions originating from the MRM panel peptides, recorded at the first time point sampled at the maximum WHO grade (treatment escalation level) for a given patient, plotted against the WHO grade. Statistical testing was carried out by including the patient age as covariate, as described previously, and, for visualization, a correction for the impact of age as covariate was performed [2].

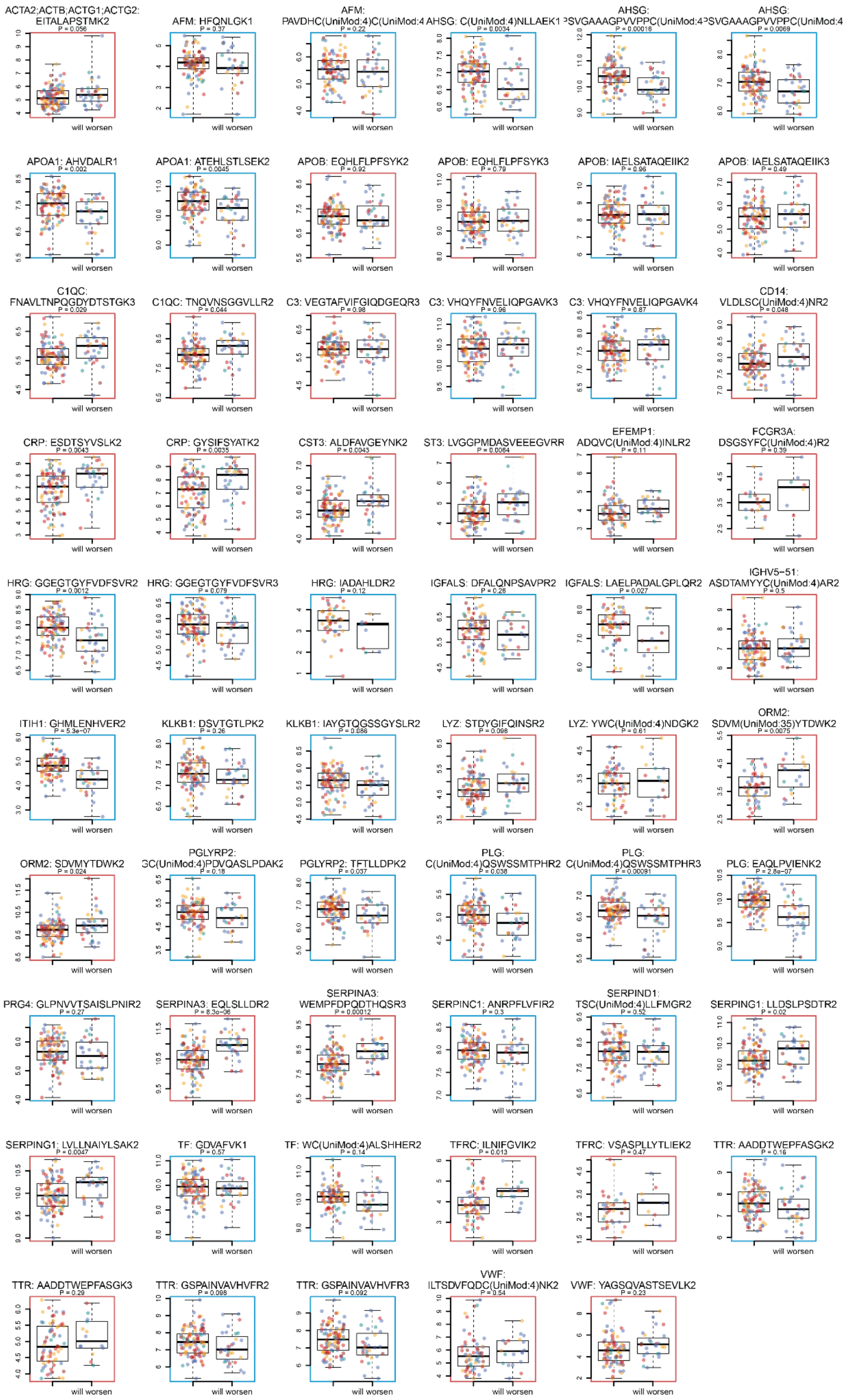

Supplementary Figure 4. Relationship between the quantities of selected precursor ions and the future disease worsening. Illustrated are the log2-transformed intensities of precursor ions originating from the MRM panel peptides, recorded at the first sampling time point. Statistical testing was carried out by including the patient age and current WHO grade as covariates, as described previously, and, for visualization, a correction for the impact of the WHO grade and age as covariates was performed [2].

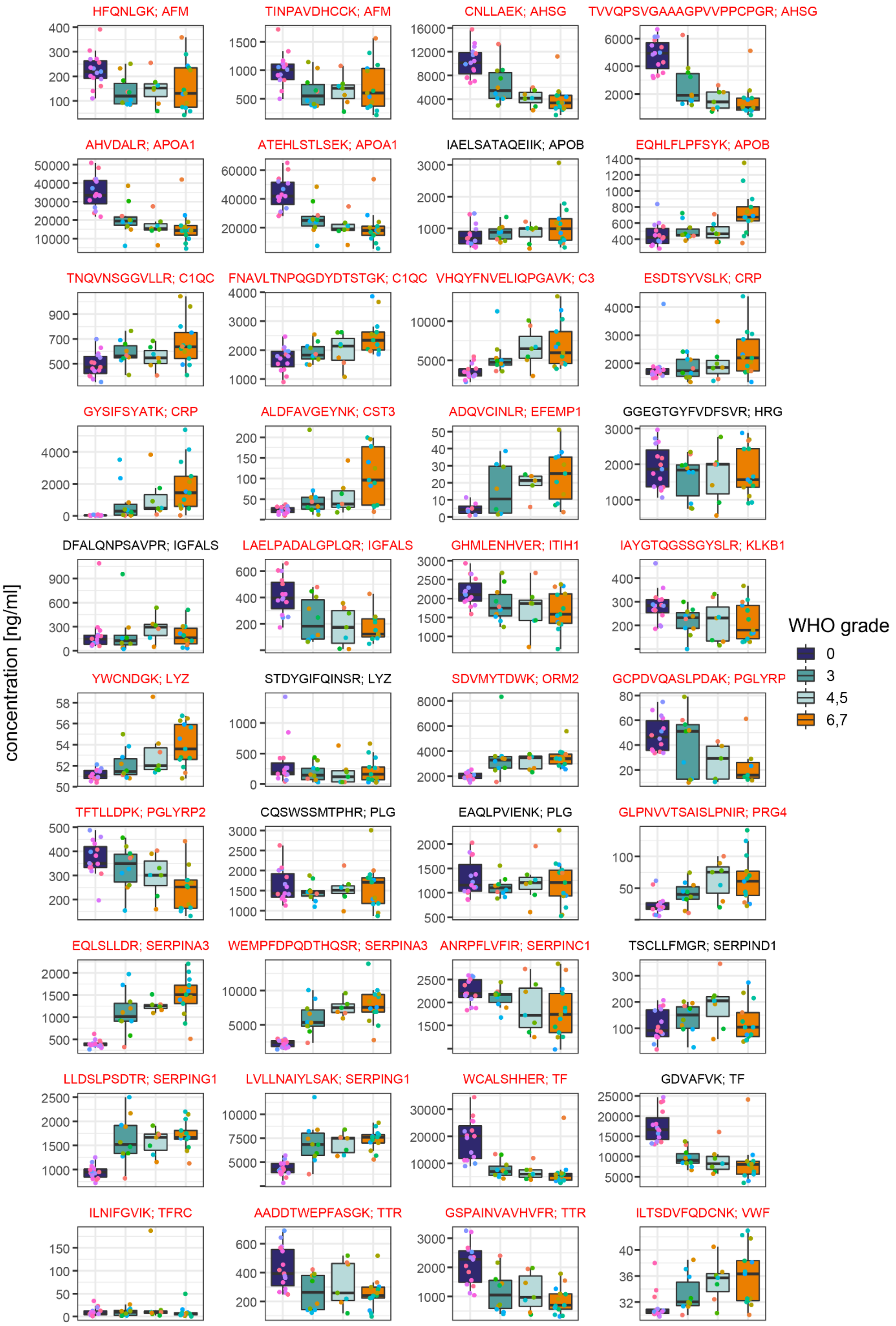

Supplementary Figure 5. Abundance of selected peptides in different COVID-19 severity groups and healthy control samples. Absolute concentration of all peptides quantified in cohort 2 (citrate plasma samples obtained during the ‘first wave’ in March 2020) plotted against the COVID-19 treatment escalation score. Note that the cohort also includes healthy COVID-19 negative control samples (blue; WHO score = 0). Peptides with a significant trend against COVID-19 severity as estimated on the respective WHO severity groups not-infected (0), mild (3), moderate (4 & 5) and severe (6 & 7) disease are highlighted in red ($P < 0.05$ after multiple testing correction). Data from n=15 (WHO 0), n=10 (WHO 3), n=4 (WHO 4), n=3 (WHO 5), n=3 (WHO 6), n=10 (WHO7).

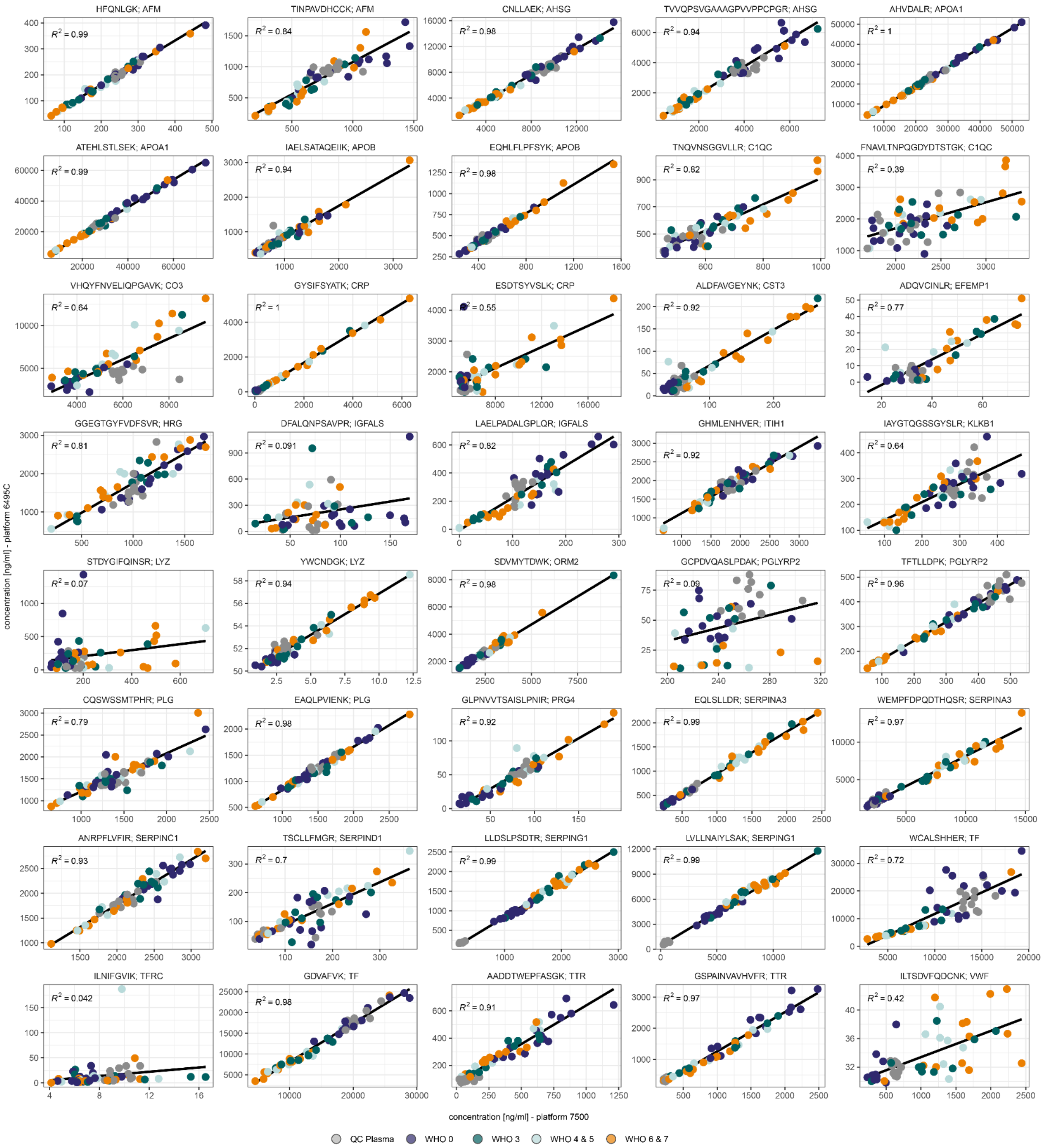

Supplementary Figure 6. Comparison of the obtained absolute quantities of the putative peptide biomarkers in plasma samples measured on two LC-MRM platforms. Samples were prepared, split in two aliquots, and measured on two different LC-MRM platforms, applying two sets of independently optimised MRM transitions, and operated in two laboratories. Shown are the linear correlations of the absolute concentration of all quantified peptides. Obtained R2 values of the linear fit as indicated. Values on the y-axis (platform 1) were measured on a 6495C (Agilent) instrument, values on y-axis (platform 2) were measured on a 7500 (SCIEX) instrument. All data based on n=15 (WHO 0), n=10 (WHO 3), n=4 (WHO 4), n=3 (WHO 5), n=3 (WHO 6), n=10 (WHO7) and n=12 (QC Plasma). Peptides are ordered alphabetically according to gene names. Peptides with poor correlation (R2 < 0.6) between both platforms are FNAVLTNPQGDYDTSTGK (C1QC), ESDTSYVSLK (CRP), DFALQNPSAVPR (IGFALS), STDYGIFQINSR (LYZ), GCPDVQASLPDAK (PGLYRP2), ILTSDVFQDCNK (VWF), ILNIFGVIK (TFRC). In the case of ESDTSYVSLK, this is largely due to one outlier (removal increases the R2 to 0.76). The other peptides are low abundant, increasing variance.

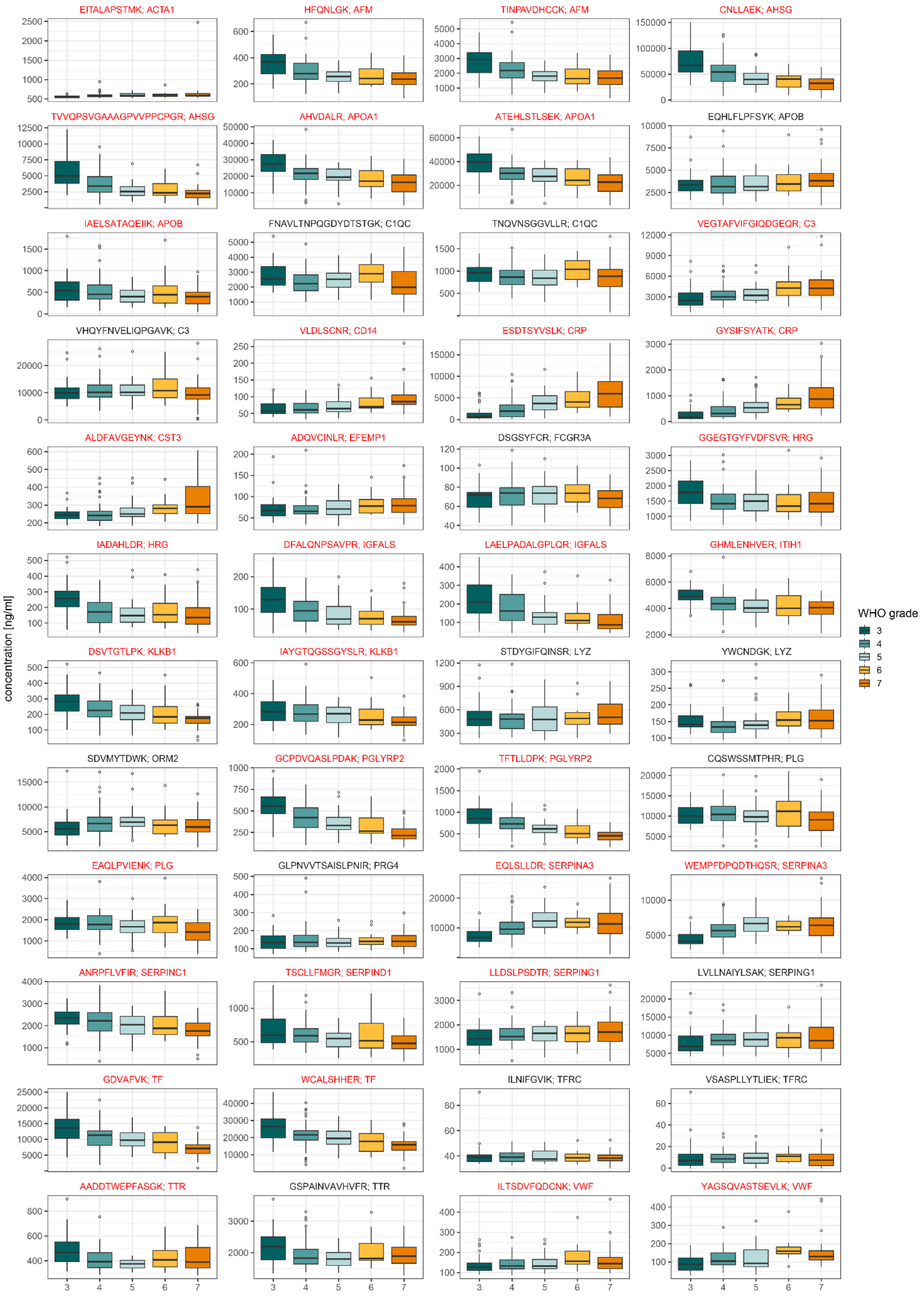

Supplementary Figure 7. Abundance of selected peptides in different COVID-19 severity groups. Absolute concentration of all 48 peptides quantified in cohort 2 (plasma samples obtained during the second wave of the pandemic) plotted against the COVID-19 treatment escalation score. Peptides with a significant trend against COVID-19 severity as estimated on the respective WHO severity score are highlighted in red (Kendall´s Tau statistics, $P < 0.05$ after multiple testing correction). Data from the first time-point obtained for each individual; n=36 (WHO 3), n=47 (WHO 4), n=27 (WHO 5), n=16 (WHO 6), n=38 (WHO7). Peptides are sorted by their corresponding gene names.

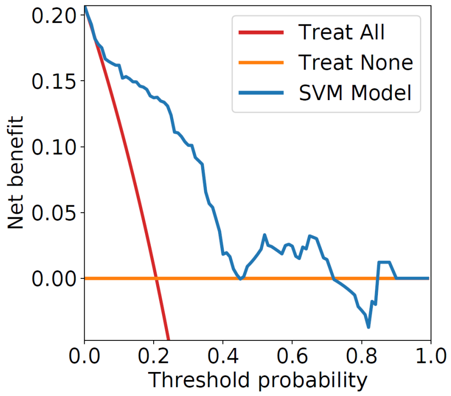

Supplementary Figure 8. Decision curve analysis. Decision curve analysis for the SVM-model (blue) presented in Figure 5. The reference strategies are also depicted (treat all, red; treat none, orange). The SVM-classifier shows the highest net benefit over a long range of threshold probabilities.

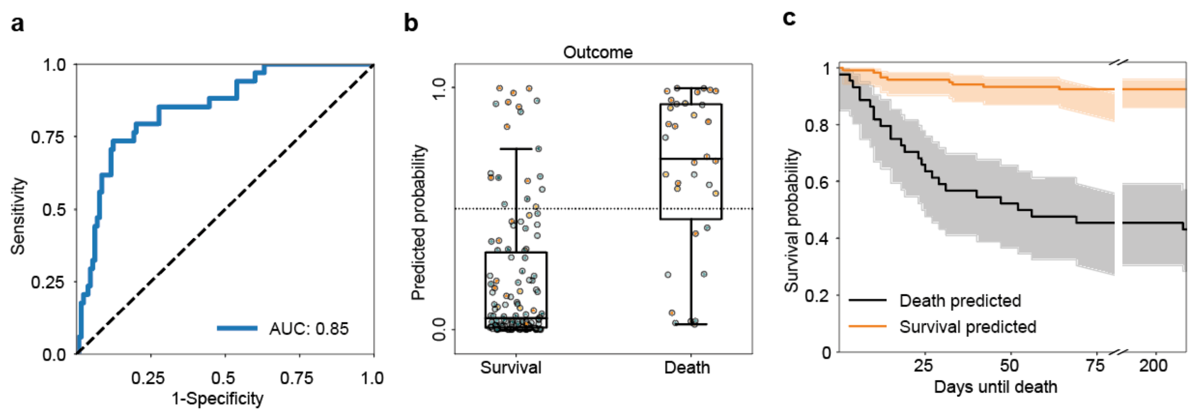

Supplementary Figure 9. COVID-19 progression prediction in a second wave COVID-19 cohort using a logistic regression model. a) ROC-Curve for the prediction of death from the first time point measured for every patient using a logistic regression classifier. **b)** Boxplot of the predicted probability sorted according to the outcome and colored with respect to the WHO grade at the day the sample was taken. **c)** Kaplan-Meier estimate of the survival function for non-survival predicted cases (black) and survival predicted cases (orange) with confidence interval (alpha=0.05). All predictions were done on withheld samples that were not used for training. Patient survival data for each timepoint is provided in Supplementary Table 13-15.

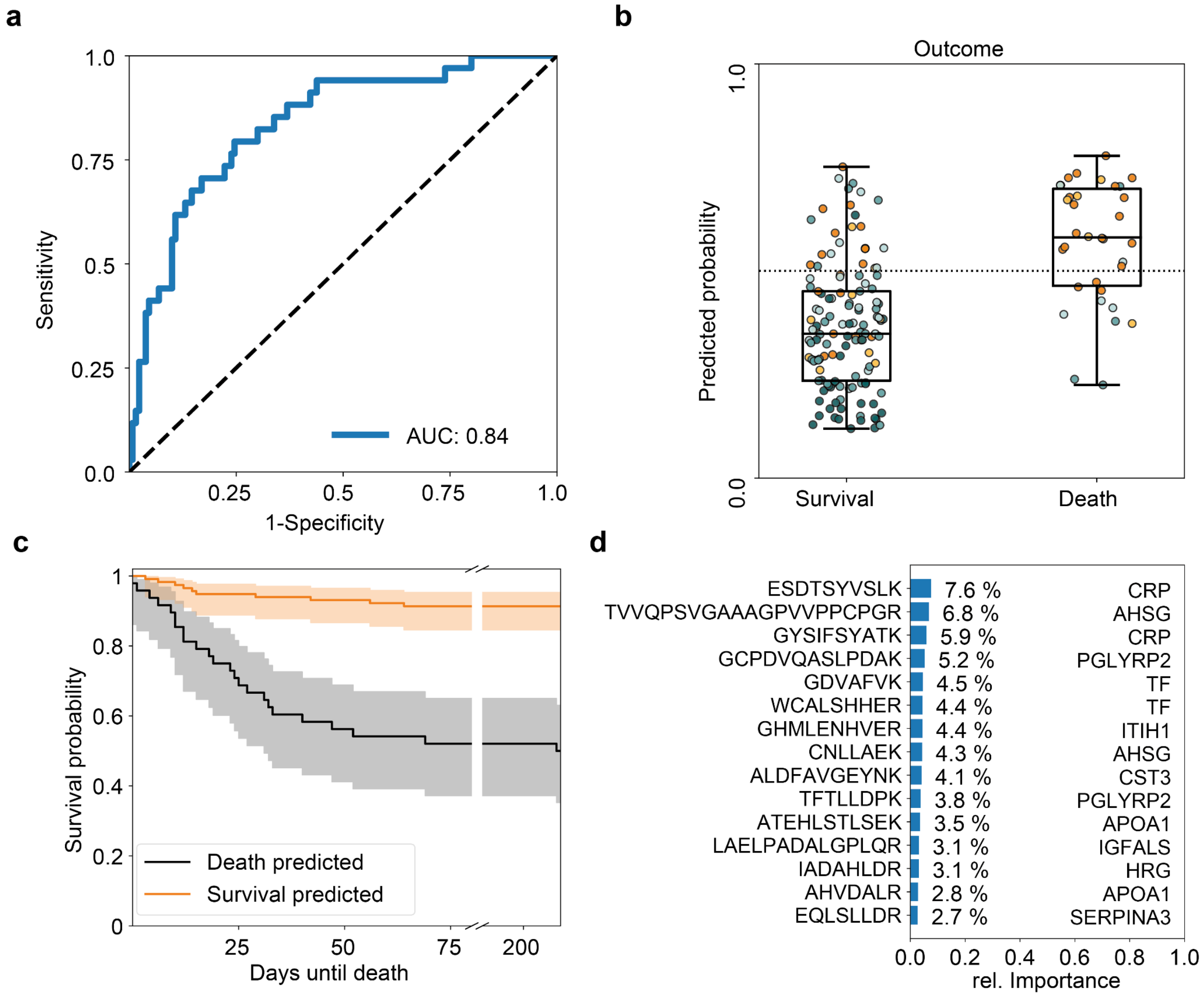

Supplementary Figure 10. COVID-19 progression prediction in a second wave COVID-19 cohort using an extra-trees model. a) ROC-Curve for the prediction of death from the first time point measured for every patient using an extra-trees classifier. **b)** Boxplot of the predicted probability sorted according to the outcome and colored with respect to the WHO grade at the day the sample was taken. Color scheme according to Fig. 4c. **c)** Kaplan-Meier estimate of the survival function for non-survival predicted cases (black) and survival predicted cases (orange) with confidence interval (alpha=0.05). All predictions were done on withheld samples that were not used for training. **d)** Feature importances for a model trained on all samples highlighting the corresponding protein/gene for each peptide. Notably, for several proteins (CRP, AHSG, PGLYRP2, TF, APOA1) more than one peptide was ranked as an important feature (Top 15 features) by the predictor. Patient survival data for each timepoint is provided in Supplementary Table 13-15.

Supplementary Table 1. Description of patient cohorts

	Cohort 1		Cohort 2 (w/o healthy controls)		Cohort 3	
n	139		30		164	
sex						
female, n (%)	44	(31.7)	10	(33.3)	39	(23.8)
male, n (%)	95	(68.3)	20	(66.7)	125	(76.2)
age, median [IQR]	61	[50-71]	62	[50-73]	60	[51-69]
body mass index, median [IQR]	27.8	[24.7-31.9]	25.7	[23.7-28.5]	29.4	[24.7-32.5]
maximum severity						
WHO3, n (%)	23	(16.5)	10	(33.3)	23	(14.0)
WHO4, n (%)	32	(23.0)	4	(13.3)	42	(25.6)
WHO5, n (%)	12	(8.6)	3	(10.0)	34	(20.7)
WHO6, n (%)	6	(4.3)	3	(10.0)	3	(1.8)
WHO7, n (%)	46	(33.1)	10	(33.3)	28	(17.1)
WHO8 (deceased), n (%)	20	(14.4)	-	-	34	(20.7)
treatment						
Dexamethasone, n (%)	-	-	-	-	112	(68.3)
Remdesivir	-	-	-	-	15	(9.1)
IMV, n (%)	69	(49.6)	13	(43.3)	61	(37.2)
ECMO, n (%)	22	(15.8)	6	(20.0)	33	(20.1)
RRT, n (%)	46	(33.1)	8	(73.3)	24	(14.6)
days hospitalized	23	[10-47]	-	-	16	[10-34]
days until discharge (w/o deceased)	20	[9-48]	-	-	12	[8-29]
days until death	28	[16-46]	-	-	32	[19-47]
sampling						
days since symptom onset, median [IQR]	-	-	-	-	13	[8-17]
days to outcome, median [IQR]	-	-	-	-	10	[5-24]

Supplementary Table 2. Multiple reaction monitoring (MRM) transitions (Agilent-6495C)

Native and labelled peptide MRM transitions on 6495C (Agilent) LC-MS/MS platform. Internal standard (labelled) peptide sequences are shown in their post-digestion form (without tryptic tags).
*- SIL internal standard not detected. Another SIL internal standard with a similar retention time was used to quantify native peptides where a corresponding SIL internal standard of that native peptide was not detected: AADDTWEPFASGK(U-13C6,15N2 was used for ASDTAMYYCAR, GYSIFSYATK(U-13C6,15N2) for GSPAINVAVHVFR and WEMPFDPQDTHQSR, ANRPFLVFIR(U-13C6,15N4) for LAELPADALGPLQR and VSASPLLYTLIEK(U-13C6,15N2) for VEGTAFVIFGIQDGEQR. **- due to low signal intensity different ion type transitions were used for corresponding native and SIL internal standard peptides for quantification.

Peptide Standard Sequence	Standard type	Precursor(m/z)	Product(m/z)	Dwell time (ms)	Collision Energy	Fragmentor	Cell Accelerator Voltage	Retention time (min)	Delta Retention time (min)
GLPNVVTSAISLPNIR	Native	825.98	740.93	12.92	26.60	166.00	5.00	5.88	1.00
		825.98	499.30	12.92	26.60	166.00	5.00	5.88	1.00
		825.98	1170.68	12.92	26.60	166.00	5.00	5.88	1.00
GLPNVVTSAISLPNIR(U-13C6,15N4)	Labelled	830.98	745.93	12.92	26.60	166.00	5.00	5.88	1.00
		830.98	509.31	12.92	26.60	166.00	5.00	5.88	1.00
GHMLENHVER	Native	407.86	514.25	14.75	9.90	166.00	5.00	1.67	1.00
		407.86	448.73	14.75	9.90	166.00	5.00	1.67	1.00
		407.86	410.18	14.75	9.90	166.00	5.00	1.67	1.00
GHMLENHVER(U-13C6,15N4)	Labelled	411.20	519.26	14.75	9.90	166.00	5.00	1.67	1.00
		411.20	453.74	14.75	9.90	166.00	5.00	1.67	1.00
EAQLPVIENK	Native	570.82	699.40	2.12	18.70	166.00	5.00	3.56	1.00
		570.82	503.28	2.12	18.70	166.00	5.00	3.56	1.00
		570.82	812.49	2.12	18.70	166.00	5.00	3.56	1.00
EAQLPVIENK(U-13C6,15N2)	Labelled	574.82	707.42	2.12	18.70	166.00	5.00	3.56	1.00
		574.82	820.50	2.12	18.70	166.00	5.00	3.56	1.00
CQSWSSMTPHR	Native	459.53	510.28	3.61	11.70	166.00	5.00	3.04	1.00
		459.53	641.32	3.61	11.70	166.00	5.00	3.04	1.00
		459.53	409.23	3.61	11.70	166.00	5.00	3.04	1.00
CQSWSSMTPHR(U-13C6,15N4)	Labelled	462.87	520.29	3.61	11.70	166.00	5.00	3.04	1.00
		462.87	419.24	3.61	11.70	166.00	5.00	3.04	1.00
EITALAPSTMK	Native	581.31	634.32	1.99	19.00	166.00	5.00	3.69	1.00
		581.31	919.49	1.99	19.00	166.00	5.00	3.69	1.00
		581.31	563.29	1.99	19.00	166.00	5.00	3.69	1.00
EITALAPSTMK(U-13C6,15N2)	Labelled	585.32	642.34	1.99	19.00	166.00	5.00	3.69	1.00
		585.32	927.51	1.99	19.00	166.00	5.00	3.69	1.00
FNAVLTNPQGDYDTSTGK	Native	964.46	1383.60	2.03	30.90	166.00	5.00	3.95	1.00
		964.46	1282.55	2.03	30.90	166.00	5.00	3.95	1.00
		964.46	1168.51	2.03	30.90	166.00	5.00	3.95	1.00
FNAVLTNPQGDYDTSTGK(U-13C6,15N2)	Labelled	968.46	1290.57	2.03	30.90	166.00	5.00	3.95	1.00
		968.46	1176.53	2.03	30.90	166.00	5.00	3.95	1.00
TNQVNSGGVLLR	Native	629.35	914.54	2.55	20.50	166.00	5.00	3.52	1.00
		629.35	815.47	2.55	20.50	166.00	5.00	3.52	1.00
		629.35	701.43	2.55	20.50	166.00	5.00	3.52	1.00
TNQVNSGGVLLR(U-13C6,15N4)	Labelled	634.35	825.48	2.55	20.50	166.00	5.00	3.52	1.00
		634.35	711.44	2.55	20.50	166.00	5.00	3.52	1.00
ALDFAVGEYNK	Native	613.81	780.39	2.07	20.00	166.00	5.00	4.30	1.00
		613.81	709.35	2.07	20.00	166.00	5.00	4.30	1.00
ALDFAVGEYNK(U-13C6,15N2)	Labelled	617.81	788.40	2.07	20.00	166.00	5.00	4.30	1.00
		617.81	717.37	2.07	20.00	166.00	5.00	4.30	1.00
LVGGPMDASVEEEGVRR	Native	600.97	745.40	2.18	16.80	166.00	5.00	3.59	1.00
		600.97	616.35	2.18	16.80	166.00	5.00	3.59	1.00
		600.97	737.85	2.18	16.80	166.00	5.00	3.59	1.00
		600.97	689.32	2.18	16.80	166.00	5.00	3.59	1.00
LVGGPMDASVEEEGVR(U-13C6,15N4)R**	Labelled	607.64	765.41	2.18	16.80	166.00	5.00	3.59	1.00
		607.64	636.37	2.18	16.80	166.00	5.00	3.59	1.00
		607.64	747.86	2.18	16.80	166.00	5.00	3.59	1.00
		607.64	699.33	2.18	16.80	166.00	5.00	3.59	1.00
SDVMYTDWK	Native	572.75	942.44	1.98	18.80	166.00	5.00	3.82	1.00
		572.75	843.37	1.98	18.80	166.00	5.00	3.82	1.00
		572.75	712.33	1.98	18.80	166.00	5.00	3.82	1.00
SDVMYTDWK(U-13C6,15N2)	Labelled	576.76	851.38	1.98	18.80	166.00	5.00	3.82	1.00
		576.76	720.34	1.98	18.80	166.00	5.00	3.82	1.00
WEMPFDPQDTHQSR	Native	591.93	968.45	2.49	16.50	166.00	5.00	4.51	1.00
		591.93	729.83	2.49	16.50	166.00	5.00	4.51	1.00
		591.93	664.30	2.49	16.50	166.00	5.00	4.51	1.00
WEMPFDPQDTHQSR(U-13C6,15N4)*	Labelled	595.26	734.83	2.49	16.50	166.00	5.00	4.51	1.00
		595.26	669.31	2.49	16.50	166.00	5.00	4.51	1.00
EQLSLLDR	Native	487.27	716.43	1.99	16.10	166.00	5.00	3.85	1.00
		487.27	603.35	1.99	16.10	166.00	5.00	3.85	1.00
EQLSLLDR(U-13C6,15N4)	Labelled	492.27	726.44	1.99	16.10	166.00	5.00	3.85	1.00
		492.27	613.35	1.99	16.10	166.00	5.00	3.85	1.00
GDVAFVK	Native	368.21	563.36	3.99	12.40	166.00	5.00	2.93	1.00
		368.21	464.29	3.99	12.40	166.00	5.00	2.93	1.00
		368.21	393.25	3.99	12.40	166.00	5.00	2.93	1.00
		368.21	246.18	3.99	12.40	166.00	5.00	2.93	1.00
		368.21	147.11	3.99	12.40	166.00	5.00	2.93	1.00
GDVAFVK(U-13C6,15N2)	Labelled	372.21	571.37	3.99	12.40	166.00	5.00	2.93	1.00
		372.21	472.30	3.99	12.40	166.00	5.00	2.93	1.00
		372.21	401.26	3.99	12.40	166.00	5.00	2.93	1.00

		372.21	254.20	3.99	12.40	166.00	5.00	2.93	1.00
		372.21	155.13	3.99	12.40	166.00	5.00	2.93	1.00
WCALSHHER	Native	399.19	505.24	8.15	9.60	166.00	5.00	2.09	1.00
		399.19	425.22	8.15	9.60	166.00	5.00	2.09	1.00
WCALSHHER(U-13C6,15N4)	Labelled	402.52	510.24	8.15	9.60	166.00	5.00	2.09	1.00
		402.52	430.22	8.15	9.60	166.00	5.00	2.09	1.00
IAELSATAQEIIK	Native	693.90	960.54	3.43	22.50	166.00	5.00	4.63	1.00
		693.90	873.50	3.43	22.50	166.00	5.00	4.63	1.00
		693.90	802.47	3.43	22.50	166.00	5.00	4.63	1.00
IAELSATAQEIIK(U-13C6,15N2)	Labelled	697.90	968.55	3.43	22.50	166.00	5.00	4.63	1.00
		697.90	881.52	3.43	22.50	166.00	5.00	4.63	1.00
		697.90	810.48	3.43	22.50	166.00	5.00	4.63	1.00
EQHLFLPFSYK	Native	470.25	641.33	7.21	12.10	166.00	5.00	5.03	1.00
		470.25	544.28	7.21	12.10	166.00	5.00	5.03	1.00
		470.25	655.32	7.21	12.10	166.00	5.00	5.03	1.00
EQHLFLPFSYK(U-13C6,15N2)	Labelled	472.92	649.34	7.21	12.10	166.00	5.00	5.03	1.00
		472.92	552.29	7.21	12.10	166.00	5.00	5.03	1.00
ADQVCINLR	Native	544.78	774.43	2.12	17.90	166.00	5.00	3.50	1.00
		544.78	675.36	2.12	17.90	166.00	5.00	3.50	1.00
		544.78	402.25	2.12	17.90	166.00	5.00	3.50	1.00
ADQVCINLR(U-13C6,15N4)	Labelled	549.78	784.44	2.12	17.90	166.00	5.00	3.50	1.00
		549.78	685.37	2.12	17.90	166.00	5.00	3.50	1.00
ILTSDVFQDCNK	Native	720.35	1112.47	2.09	23.30	166.00	5.00	4.01	1.00
		720.35	1025.44	2.09	23.30	166.00	5.00	4.01	1.00
		720.35	664.27	2.09	23.30	166.00	5.00	4.01	1.00
ILTSDVFQDCNK(U-13C6,15N2)	Labelled	724.35	1120.48	2.09	23.30	166.00	5.00	4.01	1.00
		724.35	1033.45	2.09	23.30	166.00	5.00	4.01	1.00
YAGSQVASTSEVLK	Native	720.37	933.53	2.38	23.30	166.00	5.00	3.39	1.00
		720.37	834.46	2.38	23.30	166.00	5.00	3.39	1.00
		720.37	763.42	2.38	23.30	166.00	5.00	3.39	1.00
YAGSQVASTSEVLK(U-13C6,15N2)	Labelled	724.38	941.54	2.38	23.30	166.00	5.00	3.39	1.00
		724.38	842.47	2.38	23.30	166.00	5.00	3.39	1.00
		724.38	771.43	2.38	23.30	166.00	5.00	3.39	1.00
ESDTSYVSLK	Native	564.77	696.39	3.38	18.50	166.00	5.00	3.01	1.00
		564.77	609.36	3.38	18.50	166.00	5.00	3.01	1.00
		564.77	446.30	3.38	18.50	166.00	5.00	3.01	1.00
ESDTSYVSLK(U-13C6,15N2)**	Labelled	568.78	617.37	3.38	18.50	166.00	5.00	3.01	1.00
		568.78	454.31	3.38	18.50	166.00	5.00	3.01	1.00
GYSIFSYATK	Native	568.78	916.48	2.49	18.60	166.00	5.00	4.51	1.00
		568.78	829.45	2.49	18.60	166.00	5.00	4.51	1.00
		568.78	716.36	2.49	18.60	166.00	5.00	4.51	1.00
GYSIFSYATK(U-13C6,15N2)**	Labelled	572.79	924.49	2.49	18.60	166.00	5.00	4.51	1.00
		572.79	724.38	2.49	18.60	166.00	5.00	4.51	1.00
STDYGIFQINSR	Native	700.84	934.51	2.72	22.70	166.00	5.00	4.54	1.00
		700.84	764.40	2.72	22.70	166.00	5.00	4.54	1.00
		700.84	617.34	2.72	22.70	166.00	5.00	4.54	1.00
STDYGIFQINSR(U-13C6,15N4)	Labelled	705.85	944.52	2.72	22.70	166.00	5.00	4.54	1.00
		705.85	774.41	2.72	22.70	166.00	5.00	4.54	1.00
		705.85	627.34	2.72	22.70	166.00	5.00	4.54	1.00
YWCNDGK	Native	471.69	779.31	11.56	15.60	166.00	5.00	1.95	1.00
		471.69	593.23	11.56	15.60	166.00	5.00	1.95	1.00
YWCNDGK(U-13C6,15N2)	Labelled	475.70	787.33	11.56	15.60	166.00	5.00	1.95	1.00
		475.70	601.25	11.56	15.60	166.00	5.00	1.95	1.00
AHVDALR	Native	261.15	288.20	21.50	4.60	166.00	5.00	1.66	2.00
		261.15	423.20	21.50	4.60	166.00	5.00	1.66	2.00
AHVDALR(U-13C6,15N4)	Labelled	264.48	298.21	21.50	4.60	166.00	5.00	1.66	2.00
		264.48	423.20	21.50	4.60	166.00	5.00	1.66	2.00
ATEHLSTLSEK	Native	405.88	522.27	6.70	9.80	166.00	5.00	2.23	1.00
		405.88	457.75	6.70	9.80	166.00	5.00	2.23	1.00
ATEHLSTLSEK(U-13C6,15N2)	Labelled	408.55	526.28	6.70	9.80	166.00	5.00	2.23	1.00
		408.55	461.76	6.70	9.80	166.00	5.00	2.23	1.00
TVVQPSVGAAAGPVVPPCPGR	Native	672.69	683.33	2.02	19.40	166.00	5.00	4.18	1.00
		672.69	489.76	2.02	19.40	166.00	5.00	4.18	1.00
		672.69	1038.56	2.02	19.40	166.00	5.00	4.18	1.00
TVVQPSVGAAAGPVVPPCPGR(U-13C6,15N4)	Labelled	676.03	693.34	2.02	19.40	166.00	5.00	4.18	1.00
		676.03	494.77	2.02	19.40	166.00	5.00	4.18	1.00
CNLLAEK	Native	424.22	573.36	5.01	14.20	166.00	5.00	2.85	1.00
		424.22	460.28	5.01	14.20	166.00	5.00	2.85	1.00
		424.22	347.19	5.01	14.20	166.00	5.00	2.85	1.00
CNLLAEK(U-13C6,15N2)	Labelled	428.23	581.37	5.01	14.20	166.00	5.00	2.85	1.00
		428.23	355.21	5.01	14.20	166.00	5.00	2.85	1.00
GGEGTGYFVDFSVR	Native	497.57	623.31	5.59	13.10	166.00	5.00	5.20	1.00
		497.57	508.29	5.59	13.10	166.00	5.00	5.20	1.00
		497.57	624.31	5.59	13.10	166.00	5.00	5.20	1.00
GGEGTGYFVDFSVR(U-13C6,15N4)**	Labelled	500.90	518.30	5.59	13.10	166.00	5.00	5.20	1.00
		500.90	629.31	5.59	13.10	166.00	5.00	5.20	1.00
IADAHLDR	Native	304.16	403.23	9.24	6.10	166.00	5.00	2.12	1.00

		304.16	399.20	9.24	6.10	166.00	5.00	2.12	1.00
		304.16	363.68	9.24	6.10	166.00	5.00	2.12	1.00
IADAHLDR(U-13C6,15N4)	Labelled	307.50	404.20	9.24	6.10	166.00	5.00	2.12	1.00
		307.50	368.68	9.24	6.10	166.00	5.00	2.12	1.00
GCPDVQASLPDAK	Native	679.32	1140.59	2.40	22.10	166.00	5.00	3.28	1.00
		679.32	430.23	2.40	22.10	166.00	5.00	3.28	1.00
		679.32	570.80	2.40	22.10	166.00	5.00	3.28	1.00
GCPDVQASLPDAK(U-13C6,15N2)	Labelled	683.33	1148.60	2.40	22.10	166.00	5.00	3.28	1.00
		683.33	574.81	2.40	22.10	166.00	5.00	3.28	1.00
TFTLLDPK	Native	467.77	686.41	2.32	15.50	166.00	5.00	4.43	1.00
		467.77	585.36	2.32	15.50	166.00	5.00	4.43	1.00
		467.77	472.28	2.32	15.50	166.00	5.00	4.43	1.00
TFTLLDPK(U-13C6,15N2)	Labelled	471.77	694.42	2.32	15.50	166.00	5.00	4.43	1.00
		471.77	593.37	2.32	15.50	166.00	5.00	4.43	1.00
GSPAINVAVHVFR	Native	456.26	558.31	2.80	11.60	166.00	5.00	4.52	1.00
		456.26	563.33	2.80	11.60	166.00	5.00	4.52	1.00
		456.26	527.81	2.80	11.60	166.00	5.00	4.52	1.00
GSPAINVAVHVFR(U-13C6,15N4)*	Labelled	459.59	568.33	2.80	11.60	166.00	5.00	4.52	1.00
		459.59	532.82	2.80	11.60	166.00	5.00	4.52	1.00
AADDTWEPFASGK	Native	697.81	921.45	2.02	22.60	166.00	5.00	4.08	1.00
		697.81	735.37	2.02	22.60	166.00	5.00	4.08	1.00
		697.81	606.32	2.02	22.60	166.00	5.00	4.08	1.00
AADDTWEPFASGK(U-13C6,15N2)	Labelled	701.82	929.46	2.02	22.60	166.00	5.00	4.08	1.00
		701.82	614.34	2.02	22.60	166.00	5.00	4.08	1.00
DSVTGTLPK	Native	459.25	715.43	4.31	15.20	166.00	5.00	2.89	1.00
		459.25	616.37	4.31	15.20	166.00	5.00	2.89	1.00
		459.25	515.32	4.31	15.20	166.00	5.00	2.89	1.00
DSVTGTLPK(U-13C6,15N2)	Labelled	463.26	624.38	4.31	15.20	166.00	5.00	2.89	1.00
		463.26	523.33	4.31	15.20	166.00	5.00	2.89	1.00
IAYGTQGSSGYSLR	Native	730.36	954.46	2.60	23.60	166.00	5.00	3.54	1.00
		730.36	826.41	2.60	23.60	166.00	5.00	3.54	1.00
		730.36	682.35	2.60	23.60	166.00	5.00	3.54	1.00
IAYGTQGSSGYSLR(U-13C6,15N4)	Labelled	735.37	964.47	2.60	23.60	166.00	5.00	3.54	1.00
		735.37	692.36	2.60	23.60	166.00	5.00	3.54	1.00
ANRPFLVFIR	Native	411.58	534.34	5.80	10.00	166.00	5.00	5.01	1.00
		411.58	435.27	5.80	10.00	166.00	5.00	5.01	1.00
ANRPFLVFIR(U-13C6,15N4)	Labelled	414.92	544.35	5.80	10.00	166.00	5.00	5.01	1.00
		414.92	445.28	5.80	10.00	166.00	5.00	5.01	1.00
TSCLLFMGR	Native	542.77	896.45	2.90	17.80	166.00	5.00	4.64	1.00
		542.77	736.42	2.90	17.80	166.00	5.00	4.64	1.00
		542.77	623.33	2.90	17.80	166.00	5.00	4.64	1.00
TSCLLFMGR(U-13C6,15N4)	Labelled	547.77	906.46	2.90	17.80	166.00	5.00	4.64	1.00
		547.77	633.34	2.90	17.80	166.00	5.00	4.64	1.00
HFQNLGK	Native	281.82	317.22	13.01	5.30	166.00	5.00	1.60	2.00
		281.82	413.19	13.01	5.30	166.00	5.00	1.60	2.00
		281.82	527.24	13.01	5.30	166.00	5.00	1.60	2.00
HFQNLGK(U-13C6,15N2)	Labelled	284.49	413.19	13.01	5.30	166.00	5.00	1.60	2.00
		284.49	527.24	13.01	5.30	166.00	5.00	1.60	2.00
TINPAVDHCCK	Native	438.87	550.73	7.32	11.00	166.00	5.00	2.13	1.00
		438.87	493.71	7.32	11.00	166.00	5.00	2.13	1.00
TINPAVDHCCK(U-13C6,15N2)	Labelled	441.54	554.74	7.32	11.00	166.00	5.00	2.13	1.00
		441.54	497.72	7.32	11.00	166.00	5.00	2.13	1.00
LLDSLPSDTR	Native	558.80	775.39	2.06	18.30	166.00	5.00	3.69	1.00
		558.80	575.28	2.06	18.30	166.00	5.00	3.69	1.00
		558.80	542.32	2.06	18.30	166.00	5.00	3.69	1.00
LLDSLPSDTR(U-13C6,15N4)	Labelled	563.80	785.40	2.06	18.30	166.00	5.00	3.69	1.00
		563.80	585.29	2.06	18.30	166.00	5.00	3.69	1.00
LVLLNAIYLSAK	Native	659.41	879.49	17.14	21.40	166.00	5.00	5.91	1.00
		659.41	694.41	17.14	21.40	166.00	5.00	5.91	1.00
		659.41	581.33	17.14	21.40	166.00	5.00	5.91	1.00
LVLLNAIYLSAK(U-13C6,15N2)	Labelled	663.42	887.51	17.14	21.40	166.00	5.00	5.91	1.00
		663.42	589.34	17.14	21.40	166.00	5.00	5.91	1.00
ILNIFGVIK	Native	508.83	790.48	29.95	16.80	166.00	5.00	5.99	1.00
		508.83	676.44	29.95	16.80	166.00	5.00	5.99	1.00
		508.83	563.36	29.95	16.80	166.00	5.00	5.99	1.00
ILNIFGVIK(U-13C6,15N2)	Labelled	512.84	798.50	29.95	16.80	166.00	5.00	5.99	1.00
		512.84	571.37	29.95	16.80	166.00	5.00	5.99	1.00
VSASPLLYTLIEK	Native	717.42	1089.66	10.80	23.20	166.00	5.00	5.84	1.00
		717.42	879.52	10.80	23.20	166.00	5.00	5.84	1.00
		717.42	545.33	10.80	23.20	166.00	5.00	5.84	1.00
VSASPLLYTLIEK(U-13C6,15N2)	Labelled	721.42	887.53	10.80	23.20	166.00	5.00	5.84	1.00
		721.42	549.34	10.80	23.20	166.00	5.00	5.84	1.00
DSGSYFCR	Native	496.20	789.33	5.56	16.40	166.00	5.00	2.82	1.00
		496.20	732.31	5.56	16.40	166.00	5.00	2.82	1.00
		496.20	645.28	5.56	16.40	166.00	5.00	2.82	1.00
DSGSYFCR(U-13C6,15N4)	Labelled	501.20	799.34	5.56	16.40	166.00	5.00	2.82	1.00
		501.20	655.29	5.56	16.40	166.00	5.00	2.82	1.00

VLDLSCNR	Native	488.75	877.42	3.68	16.20	166.00	5.00	3.02	1.00
		488.75	764.34	3.68	16.20	166.00	5.00	3.02	1.00
		488.75	649.31	3.68	16.20	166.00	5.00	3.02	1.00
VLDLSCNR(U-13C6,15N4)	Labelled	493.75	774.34	3.68	16.20	166.00	5.00	3.02	1.00
		493.75	659.32	3.68	16.20	166.00	5.00	3.02	1.00
DFALQNPSAVPR	Native	657.84	981.55	2.07	21.40	166.00	5.00	4.15	1.00
		657.84	740.40	2.07	21.40	166.00	5.00	4.15	1.00
		657.84	626.36	2.07	21.40	166.00	5.00	4.15	1.00
DFALQNPSAVPR(U-13C6,15N4)	Labelled	662.85	750.41	2.07	21.40	166.00	5.00	4.15	1.00
		662.85	636.37	2.07	21.40	166.00	5.00	4.15	1.00
LAELPADALGPLQR	Native	732.41	1037.57	6.28	23.70	166.00	5.00	4.97	1.00
		732.41	570.34	6.28	23.70	166.00	5.00	4.97	1.00
		732.41	519.29	6.28	23.70	166.00	5.00	4.97	1.00
LAELPADALGPLQR(U-13C6,15N4)*	Labelled	737.42	1047.58	6.28	23.70	166.00	5.00	4.97	1.00
		737.42	524.29	6.28	23.70	166.00	5.00	4.97	1.00
ASDTAMYYCAR	Native	654.77	934.39	2.14	21.30	166.00	5.00	4.19	1.00
		654.77	863.35	2.14	21.30	166.00	5.00	4.19	1.00
		654.77	732.31	2.14	21.30	166.00	5.00	4.19	1.00
		654.77	569.25	2.14	21.30	166.00	5.00	4.19	1.00
ASDTAMYYCAR(U-13C6,15N4)*	Labelled	659.78	944.40	2.14	21.30	166.00	5.00	4.19	1.00
		659.78	873.36	2.14	21.30	166.00	5.00	4.19	1.00
		659.78	742.32	2.14	21.30	166.00	5.00	4.19	1.00
		659.78	579.26	2.14	21.30	166.00	5.00	4.19	1.00
VEGTAFVIFGIQDGEQR	Native	622.65	732.33	9.89	17.60	166.00	5.00	5.61	1.00
		622.65	604.27	9.89	17.60	166.00	5.00	5.61	1.00
		622.65	489.24	9.89	17.60	166.00	5.00	5.61	1.00
VEGTAFVIFGIQDGEQR(U-13C6,15N4)*	Labelled	625.99	742.34	9.89	17.60	166.00	5.00	5.61	1.00
		625.99	614.28	9.89	17.60	166.00	5.00	5.61	1.00
		625.99	499.25	9.89	17.60	166.00	5.00	5.61	1.00
VHQYFNVELIQPGAVK	Native	614.67	825.52	5.42	17.30	166.00	5.00	4.91	1.00
		614.67	712.44	5.42	17.30	166.00	5.00	4.91	1.00
		614.67	471.29	5.42	17.30	166.00	5.00	4.91	1.00
VHQYFNVELIQPGAVK(U-13C6,15N2)	Labelled	617.34	720.45	5.42	17.30	166.00	5.00	4.91	1.00
		617.34	479.31	5.42	17.30	166.00	5.00	4.91	1.00

Supplementary Table 3. Multiple reaction monitoring (MRM) transitions (Sciex-7500)

Native and labelled peptide MRM transitions on 7500 (SCIEX) LC-MS/MS platform. Internal standard (labelled) peptide sequences are shown in their post-digestion form (without tryptic tags).

EP - entrance potential; CE - collision energy; CXP - collision cell exit potential; DP - declustering potential; RT - retention time.

Note: whilst it is not necessary to specify DP on 7500 platform due to a novel ion source design, we have experimentally optimised DP on 5500 (SCIEX) platform as users of 6500, 5500 and similar SCIEX platforms may find it useful when implementing this method. *- SIL internal standard not detected. Another SIL internal standard with a similar retention time was used to quantify native peptides where a corresponding SIL internal standard of that native peptide was not detected: YAGSQVASTSEVLK(U-13C6,15N2) was used for ESDTSYVSLK, TSCLLFMGR(U-13C6,15N4) for VHQYFNVELIQPGAVK and ANRPFLVFIR(U-13C6,15N4) for LAELPADALGPLQR.

Peptide Standard Sequence	Standard type	Precusor (Q1) m/z	Product (Q3) m/z	Dwell time (ms)	EP	CE	CXP	DP	RT (min)	RT tolerance (s)
GLPNVVTSAISLPNIR	Native	825.98	1170.68	32.3	10	43.30	25	176	7.90	15
		825.98	1071.62	32.3	10	43.30	25	176	7.90	15
		825.98	499.30	32.3	10	31.30	25	176	7.90	15
GLPNVVTSAISLPNIR(U-13C6,15N4)	Labelled	830.98	1180.69	3.0	10	43.30	25	176	7.90	15
		830.98	1081.62	3.0	10	43.30	25	176	7.90	15
GHMLENHVER	Native	407.86	783.37	43.3	10	20.20	25	132	2.63	15
		407.86	403.23	43.3	10	24.20	25	132	2.63	15
GHMLENHVER(U-13C6,15N4)	Labelled	411.20	793.38	3.0	10	20.20	25	132	2.63	15
		411.20	413.23	3.0	10	24.20	25	132	2.63	15
EAQLPVIENK	Native	570.82	699.40	19.3	10	26.50	25	142	5.03	15
		570.82	503.28	19.3	10	34.50	25	142	5.03	15
		570.82	390.20	19.3	10	34.50	25	142	5.03	15
EAQLPVIENK(U-13C6,15N2)	Labelled	574.84	707.41	3.0	10	26.50	25	142	5.03	15
		574.84	511.30	3.0	10	34.50	25	142	5.03	15
CQSWSSMTPHR	Native	459.53	510.28	20.4	10	22.60	25	124	4.17	15
		459.53	409.23	20.4	10	22.60	25	124	4.17	15
CQSWSSMTPHR(U-13C6,15N4)	Labelled	462.86	520.28	3.0	10	22.60	25	124	4.17	15
		462.86	419.23	3.0	10	22.60	25	124	4.17	15
EITALAPSTMK	Native	581.31	747.41	21.8	10	27.10	25	140	5.20	15
		581.31	634.32	21.8	10	23.10	25	140	5.20	15
		581.31	563.29	21.8	10	23.10	25	140	5.20	15
EITALAPSTMK(U-13C6,15N2)	Labelled	585.31	755.42	3.0	10	27.10	25	140	5.20	15
		585.31	642.33	3.0	10	23.10	25	140	5.20	15
FNAVLTNPQGDYDTSTGK	Native	643.31	1168.51	21.7	10	46.20	25	142	5.38	15
		643.31	584.76	21.7	10	54.20	25	142	5.38	15
FNAVLTNPQGDYDTSTGK(U-13C6,15N2)	Labelled	645.97	1176.52	3.0	10	46.20	25	142	5.38	15
		645.97	588.76	3.0	10	54.20	25	142	5.38	15
TNQVNSGGVLLR	Native	629.35	914.54	19.0	10	33.50	25	152	4.75	15
		629.35	815.47	19.0	10	33.50	25	152	4.75	15
TNQVNSGGVLLR(U-13C6,15N4)	Labelled	634.35	924.55	3.0	10	33.50	25	152	4.75	15
		634.35	825.48	3.0	10	33.50	25	152	4.75	15
ALDFAVGEYNK	Native	613.81	709.35	19.5	10	28.70	25	140	5.95	15
		613.81	610.28	19.5	10	24.70	25	140	5.95	15
ALDFAVGEYNK(U-13C6,15N2)	Labelled	617.81	717.36	3.0	10	28.70	25	140	5.95	15
		617.81	618.29	3.0	10	24.70	25	140	5.95	15
LVGGPMDASVEEEGVRR	Native	600.97	794.87	18.8	10	25.20	25	142	4.92	15
		600.97	566.29	18.8	10	37.20	25	142	4.92	15
LVGGPMDASVEEEGVR(U-13C6,15N4)R	Labelled	604.30	799.88	3.0	10	25.20	25	142	4.92	15
		604.30	571.30	3.0	10	37.20	25	142	4.92	15
SDVMYTDWK	Native	572.75	843.37	21.4	10	26.60	25	140	5.48	15
		572.75	712.33	21.4	10	26.60	25	140	5.48	15
SDVMYTDWK(U-13C6,15N2)	Labelled	576.75	851.38	3.0	10	26.60	25	140	5.48	15
		576.75	720.34	3.0	10	26.60	25	140	5.48	15
WEMPFDPQDTHQSR	Native	591.93	968.45	14.0	10	32.80	25	138	6.28	15
		591.93	729.83	14.0	10	24.80	25	138	6.28	15
WEMPFDPQDTHQSR(U-13C6,15N4)*	Labelled	595.26	978.46	3.0	10	32.80	25	138	6.28	15
		595.26	734.82	3.0	10	24.80	25	138	6.28	15
EQLSLLDR	Native	487.27	716.43	21.2	10	22.40	25	134	5.50	15
		487.27	603.35	21.2	10	22.40	25	134	5.50	15
EQLSLLDR(U-13C6,15N4)	Labelled	492.27	726.43	3.0	10	22.40	25	134	5.50	15
		492.27	613.35	3.0	10	22.40	25	134	5.50	15
GDVAFVK	Native	368.21	563.36	19.9	10	16.40	25	112	4.20	15
		368.21	464.29	19.9	10	16.40	25	112	4.20	15
GDVAFVK(U-13C6,15N2)	Labelled	372.21	571.36	3.0	10	16.40	25	112	4.20	15
		372.21	472.30	3.0	10	16.40	25	112	4.20	15
WCALSHHER	Native	399.19	665.31	27.0	10	23.80	25	118	2.98	15
		399.19	441.22	27.0	10	27.80	25	118	2.98	15
WCALSHHER(U-13C6,15N4)	Labelled	402.52	675.31	3.0	10	23.80	25	118	2.98	15
		402.52	451.22	3.0	10	27.80	25	118	2.98	15
IAELSATAQEIIK	Native	693.90	960.54	14.3	10	32.70	25	164	6.25	15
		693.90	873.50	14.3	10	32.70	25	164	6.25	15
		693.90	802.47	14.3	10	32.70	25	164	6.25	15
IAELSATAQEIIK(U-13C6,15N2)	Labelled	697.90	968.55	3.0	10	32.70	25	164	6.25	15
		697.90	810.48	3.0	10	32.70	25	164	6.25	15
EQHLFLPFSYK	Native	470.25	641.33	43.1	10	19.10	25	120	7.01	15

		470.25	544.28	43.1	10	23.10	25	120	7.01	15
		470.25	397.21	43.1	10	27.10	25	120	7.01	15
EQHLFLPFSYK(U-13C6,15N2)	Labelled	472.91	649.34	3.0	10	19.10	25	120	7.01	15
		472.91	552.29	3.0	10	23.10	25	120	7.01	15
ADQVCINLR	Native	544.78	774.43	19.0	10	29.20	25	134	4.96	15
		544.78	675.36	19.0	10	29.20	25	134	4.96	15
ADQVCINLR(U-13C6,15N4)	Labelled	549.78	784.43	3.0	10	29.20	25	134	4.96	15
		549.78	685.36	3.0	10	29.20	25	134	4.96	15
ILTSDVFQDCNK	Native	720.35	811.34	21.0	10	34.00	25	162	5.58	15
		720.35	664.27	21.0	10	34.00	25	162	5.58	15
ILTSDVFQDCNK(U-13C6,15N2)	Labelled	724.35	819.35	3.0	10	34.00	25	162	5.58	15
		724.35	667.81	3.0	10	42.00	25	162	5.58	15
ESDTSYVSLK	Native	564.77	696.39	20.3	10	26.20	25	144	4.46	15
		564.77	609.36	20.3	10	26.20	25	144	4.46	15
ESDTSYVSLK(U-13C6,15N2)*	Labelled	568.78	704.40	3.0	10	26.20	25	144	4.46	15
		568.78	617.37	3.0	10	26.20	25	144	4.46	15
GYSIFSYATK	Native	568.78	716.36	15.9	10	26.40	25	136	6.39	15
		568.78	569.29	15.9	10	18.40	25	136	6.39	15
		568.78	482.26	15.9	10	38.40	25	136	6.39	15
GYSIFSYATK(U-13C6,15N2)	Labelled	572.79	724.37	3.0	10	26.40	25	136	6.39	15
		572.79	577.30	3.0	10	18.40	25	136	6.39	15
STDYGIFQINSR	Native	700.84	934.51	16.1	10	37.00	25	86	6.41	15
		700.84	764.40	16.1	10	33.00	25	86	6.41	15
		700.84	617.34	16.1	10	33.00	25	86	6.41	15
STDYGIFQINSR(U-13C6,15N4)	Labelled	705.84	944.51	3.0	10	37.00	25	86	6.41	15
		705.84	774.41	3.0	10	33.00	25	86	6.41	15
YAGSQVASTSEVLK	Native	720.37	933.53	19.5	10	38.00	25	172	4.66	15
		720.37	834.46	19.5	10	38.00	25	172	4.66	15
		720.37	763.42	19.5	10	38.00	25	172	4.66	15
YAGSQVASTSEVLK(U-13C6,15N2)	Labelled	724.37	941.53	3.0	10	38.00	25	172	4.66	15
		724.37	842.47	3.0	10	38.00	25	172	4.66	15
YWCNDGK	Native	471.69	779.31	37.7	10	21.60	25	132	3.22	15
		471.69	593.23	37.7	10	21.60	25	132	3.22	15
YWCNDGK(U-13C6,15N2)	Labelled	475.70	787.32	3.0	10	21.60	25	132	3.22	15
		475.70	601.24	3.0	10	21.60	25	132	3.22	15
AHVDALR	Native	391.22	573.34	53.4	10	21.60	25	132	2.59	15
		391.22	474.27	53.4	10	25.60	25	132	2.59	15
AHVDALR(U-13C6,15N4)	Labelled	396.22	583.34	3.0	10	21.60	25	132	2.59	15
		396.22	484.27	3.0	10	25.60	25	132	2.59	15
ATEHLSTLSEK	Native	405.88	664.35	41.6	10	24.10	25	120	3.26	15
		405.88	363.19	41.6	10	24.10	25	120	3.26	15
ATEHLSTLSEK(U-13C6,15N2)	Labelled	408.55	672.36	3.0	10	24.10	25	120	3.26	15
		408.55	371.20	3.0	10	24.10	25	120	3.26	15
TVVQPSVGAAAGPVVPPCPGR	Native	672.69	978.52	20.9	10	24.60	25	146	4.96	15
		672.69	683.33	20.9	10	28.60	25	146	4.96	15
		672.69	586.28	20.9	10	40.60	25	146	4.96	15
TVVQPSVGAAAGPVVPPCPGR(U-13C6,15N4)	Labelled	676.03	988.52	3.0	10	24.60	25	146	4.96	15
		676.03	693.33	3.0	10	28.60	25	146	4.96	15
GGEGTGYFVDFSVR	Native	745.85	1089.54	24.9	10	39.30	25	172	5.73	15
		745.85	869.45	24.9	10	39.30	25	172	5.73	15
		745.85	508.29	24.9	10	39.30	25	172	5.73	15
GGEGTGYFVDFSVR(U-13C6,15N4)	Labelled	750.85	1099.54	3.0	10	39.30	25	172	5.73	15
		750.85	879.45	3.0	10	39.30	25	172	5.73	15
IADAHLDR	Native	455.74	797.39	29.1	10	24.80	25	144	2.85	15
		455.74	726.35	29.1	10	24.80	25	144	2.85	15
IADAHLDR(U-13C6,15N4)	Labelled	460.74	807.40	3.0	10	24.80	25	144	2.85	15
		460.74	736.36	3.0	10	24.80	25	144	2.85	15
CNLLAEK	Native	424.22	573.36	26.8	10	23.20	25	128	4.01	15
		424.22	460.28	26.8	10	23.20	25	128	4.01	15
CNLLAEK(U-13C6,15N2)	Labelled	428.22	581.37	3.0	10	23.20	25	128	4.01	15
		428.22	468.30	3.0	10	23.20	25	128	4.01	15
GCPDVQASLPDAK	Native	453.22	630.35	20.2	10	28.00	25	120	4.58	15
		453.22	430.23	20.2	10	32.00	25	120	4.58	15
GCPDVQASLPDAK(U-13C6,15N2)	Labelled	455.89	638.35	3.0	10	28.00	25	120	4.58	15
		455.89	438.24	3.0	10	32.00	25	120	4.58	15
TFTLLDPK	Native	467.77	686.41	14.0	10	21.40	25	132	6.27	15
		467.77	585.36	14.0	10	21.40	25	132	6.27	15
		467.77	472.28	14.0	10	21.40	25	132	6.27	15
TFTLLDPK(U-13C6,15N2)	Labelled	471.77	694.42	3.0	10	21.40	25	132	6.27	15
		471.77	593.37	3.0	10	21.40	25	132	6.27	15
GSPAINVAVHVFR	Native	456.26	728.42	16.2	10	26.40	25	122	6.16	15
		456.26	558.31	16.2	10	18.40	25	122	6.16	15
		456.26	611.86	16.2	10	18.40	25	122	6.16	15
GSPAINVAVHVFR(U-13C6,15N4)*	Labelled	459.59	738.42	3.0	10	26.40	25	122	6.16	15
		459.59	616.86	3.0	10	18.40	25	122	6.16	15

AADDTWEPFASGK	Native	697.81	921.45	19.3	10	32.90	25	156	5.97	15
		697.81	735.37	19.3	10	32.90	25	156	5.97	15
		697.81	606.32	19.3	10	40.90	25	156	5.97	15
AADDTWEPFASGK(U-13C6,15N2)	Labelled	701.82	929.46	3.0	10	32.90	25	156	5.97	15
		701.82	614.33	3.0	10	40.90	25	156	5.97	15
DSVTGTLPK	Native	459.25	616.37	29.5	10	21.00	25	128	3.99	15
		459.25	515.32	29.5	10	21.00	25	128	3.99	15
DSVTGTLPK(U-13C6,15N2)	Labelled	463.25	624.38	3.0	10	21.00	25	128	3.99	15
		463.25	523.33	2.0	10	21.00	25	128	3.99	15
IAYGTQGSSGYSLR	Native	730.36	1112.53	19.0	10	38.50	25	166	4.78	15
		730.36	826.41	19.0	10	42.50	25	166	4.78	15
IAYGTQGSSGYSLR(U-13C6,15N4)	Labelled	735.36	1122.54	3.0	10	38.50	25	166	4.78	15
		735.36	836.41	3.0	10	42.50	25	166	4.78	15
ANRPFLVFIR	Native	411.58	534.34	22.8	10	20.30	25	190	6.84	15
		411.58	435.27	22.8	10	20.30	25	190	6.84	15
ANRPFLVFIR(U-13C6,15N4)	Labelled	414.91	544.34	3.0	10	20.30	25	190	6.84	15
		414.91	445.27	3.0	10	20.30	25	190	6.84	15
TSCLLFMGR	Native	542.77	623.33	18.1	10	29.10	25	134	6.53	15
		542.77	510.25	18.1	10	25.10	25	134	6.53	15
TSCLLFMGR(U-13C6,15N4)	Labelled	547.77	633.34	3.0	10	29.10	25	134	6.53	15
		547.77	520.25	3.0	10	25.10	25	134	6.53	15
HFQNLGK	Native	422.23	706.39	36.1	10	23.10	25	126	2.71	15
		422.23	559.32	36.1	10	23.10	25	126	2.71	15
HFQNLGK(U-13C6,15N2)	Labelled	426.23	714.40	3.0	10	23.10	25	126	2.71	15
		426.23	567.33	3.0	10	23.10	25	126	2.71	15
TINPAVDHCCK	Native	438.87	719.26	27.8	10	25.60	25	120	3.03	15
		438.87	604.23	27.8	10	25.60	25	120	3.03	15
TINPAVDHCCK(U-13C6,15N2)	Labelled	441.54	727.27	3.0	10	25.60	25	120	3.03	15
		441.54	612.24	3.0	10	25.60	25	120	3.03	15
LLDSLPSDTR	Native	558.80	890.42	20.1	10	25.90	25	140	5.08	15
		558.80	575.28	20.1	10	25.90	25	140	5.08	15
LLDSLPSDTR(U-13C6,15N4)	Labelled	563.80	900.42	3.0	10	25.90	25	140	5.08	15
		563.80	585.28	3.0	10	25.90	25	140	5.08	15
LVLLNAIYLSAK	Native	659.41	879.49	32.1	10	31.00	25	148	8.17	15
		659.41	765.45	32.1	10	31.00	25	148	8.17	15
		659.41	694.41	32.1	10	43.00	25	148	8.17	15
LVLLNAIYLSAK(U-13C6,15N2)	Labelled	663.41	887.50	3.0	10	31.00	25	148	8.17	15
		663.41	773.46	3.0	10	31.00	25	148	8.17	15
ILNIFGVIK	Native	508.83	790.48	43.0	10	23.40	25	132	8.24	15
		508.83	676.44	43.0	10	23.40	25	132	8.24	15
		508.83	563.36	43.0	10	23.40	25	132	8.24	15
ILNIFGVIK(U-13C6,15N2)	Labelled	512.83	798.49	3.0	10	23.40	25	132	8.24	15
		512.83	571.36	3.0	10	23.40	25	132	8.24	15
VSASPLLYTLIEK	Native	717.42	1089.66	27.3	10	29.90	25	156	8.04	15
		717.42	879.52	27.3	10	37.90	25	156	8.04	15
		717.42	766.43	27.3	10	33.90	25	156	8.04	15
VSASPLLYTLIEK(U-13C6,15N2)*	Labelled	721.42	1097.66	3.0	10	29.90	25	156	8.04	15
		721.42	774.44	3.0	10	33.90	25	156	8.04	15
DSGSYFCR	Native	496.20	789.33	23.9	10	22.80	25	84	4.06	15
		496.20	645.28	23.9	10	22.80	25	84	4.06	15
DSGSYFCR(U-13C6,15N4)	Labelled	501.20	799.34	3.0	10	22.80	25	84	4.06	15
		501.20	655.28	3.0	10	22.80	25	84	4.06	15
VLDLSCNR	Native	488.75	764.34	19.0	10	22.40	25	128	4.29	15
		488.75	649.31	19.0	10	26.40	25	128	4.29	15
		488.75	536.22	19.0	10	22.40	25	128	4.29	15
VLDLSCNR(U-13C6,15N4)	Labelled	493.75	774.34	3.0	10	22.40	25	128	4.29	15
		493.75	546.23	3.0	10	22.40	25	128	4.29	15
DFALQNPSAVPR	Native	657.84	868.46	20.7	10	34.90	25	110	5.69	15
		657.84	740.40	20.7	10	30.90	25	110	5.69	15
		657.84	626.36	20.7	10	34.90	25	110	5.69	15
DFALQNPSAVPR(U-13C6,15N4)*	Labelled	662.84	750.41	3.0	10	30.90	25	110	5.69	15
		662.84	636.37	3.0	10	34.90	25	110	5.69	15
LAELPADALGPLQR	Native	732.41	1037.57	23.6	10	34.60	25	88	6.88	15
		732.41	754.46	23.6	10	50.60	25	88	6.88	15
		732.41	570.34	23.6	10	34.60	25	88	6.88	15
LAELPADALGPLQR(U-13C6,15N4)*	Labelled	737.41	1047.52	3.0	10	34.60	25	88	6.88	15
		737.41	764.46	3.0	10	50.60	25	88	6.88	15
ASDTAMYYCAR		654.77	863.35	19.0	10	30.70	25	148	4.35	15
		654.77	732.31	19.0	10	30.70	25	148	4.35	15
ASDTAMYYCAR(U-13C6,15N4)	Labelled	659.78	873.36	3.5	10	30.70	25	148	4.35	15
		659.78	742.32	3.5	10	30.70	25	148	4.35	15
VEGTAFVIFGIQDGEQR	Native	622.65	732.33	48.5	10	26.30	25	142	7.80	15
		622.65	604.27	48.5	10	30.30	25	142	7.80	15
		622.65	525.25	48.5	10	22.30	25	142	7.80	15
VEGTAFVIFGIQDGEQR(U-13C6,15N4)*	Labelled	625.98	742.33	3.0	10	26.30	25	142	7.80	15

		625.98	530.25	3.0	10	22.30	25	142	7.80	15
VHQYFNVELIQPGAVK	Native	614.67	712.44	19.7	10	21.90	25	132	6.63	15
		614.67	471.29	19.7	10	33.90	25	132	6.63	15
VHQYFNVELIQPGAVK(U-13C6,15N2)*	Labelled	617.33	720.44	3.0	10	21.90	25	132	6.63	15
		617.33	479.30	3.0	10	33.90	25	132	6.63	15

Supplementary Table 4. Statistics result of MRM assay (Cohort 2)
Kendall´s Tau trend test between peptide quantitites and COVID19 treatment escalation score (WHO 0, 3, 4, 5, 6, 7)

Peptide	Peptide.Gene	p.value	adjusted p.value
WEMPFDPQDTHQSR	WEMPFDPQDTHQSR; SERPINA3	5,92E-08	1,33E-06
EQLSLLDR	EQLSLLDR; SERPINA3	6,63E-08	1,33E-06
CNLLAEK	CNLLAEK; AHSG	1,80E-07	1,80E-06
GYSIFSYATK	GYSIFSYATK; CRP	1,80E-07	1,80E-06
TVVQPSVGAAAGPVVPPCPGR	TVVQPSVGAAAGPVVPPCPGR; AHSG	2,78E-07	2,06E-06
GDVAFVK	GDVAFVK; TF	3,09E-07	2,06E-06
WCALSHHER	WCALSHHER; TF	7,99E-07	4,56E-06
ATEHLSTLSEK	ATEHLSTLSEK; APOA1	9,82E-07	4,91E-06
AHVDALR	AHVDALR; APOA1	1,21E-06	5,36E-06
LVLLNAIYLSAK	LVLLNAIYLSAK; SERPING1	2,97E-06	1,19E-05
ALDFAVGEYNK	ALDFAVGEYNK; CST3	3,62E-06	1,32E-05
SDVMYTDWK	SDVMYTDWK; ORM2	5,33E-06	1,78E-05
LLDSLPSDTR	LLDSLPSDTR; SERPING1	9,42E-06	2,90E-05
YWCNDGK	YWCNDGK; LYZ	1,25E-05	3,56E-05
VHQYFNVELIQPGAVK	VHQYFNVELIQPGAVK; C3	1,80E-05	4,80E-05
GSPAINVAVHVFR	GSPAINVAVHVFR; TTR	1,21E-04	3,03E-04
GLPNVVTSAISLPNIR	GLPNVVTSAISLPNIR; PRG4	1,55E-04	3,65E-04
FNAVLTNPQGDYDTSTGK	FNAVLTNPQGDYDTSTGK; C1QC	1,98E-04	4,16E-04
TFTLLDPK	TFTLLDPK; PGLYRP2	1,98E-04	4,16E-04
ILTSDVFQDCNK	ILTSDVFQDCNK; VWF	2,14E-04	4,28E-04
LAELPADALGPLQR	LAELPADALGPLQR; IGFALS	2,51E-04	4,78E-04
EQHLFLPFSYK	EQHLFLPFSYK; APOB	5,03E-04	9,14E-04
ADQVCINLR	ADQVCINLR; EFEMP1	1,88E-03	3,27E-03
TNQVNSGGVLLR	TNQVNSGGVLLR; C1QC	4,34E-03	7,24E-03
AADDTWEPFASGK	AADDTWEPFASGK; TTR	7,58E-03	1,21E-02
GCPDVQASLPDAK	GCPDVQASLPDAK; PGLYRP2	8,04E-03	0,012368
ANRPFLVFIR	ANRPFLVFIR; SERPINC1	0,010811	0,016016
GHMLENHVER	GHMLENHVER; ITIH1	0,011454	0,016363
TINPAVDHCCK	TINPAVDHCCK; AFM	0,014381	0,019835
HFQNLGK	HFQNLGK; AFM	0,01699	0,022653
IAYGTQGSSGYSLR	IAYGTQGSSGYSLR; KLKB1	0,017948	0,023158
ESDTSYVSLK	ESDTSYVSLK; CRP	0,02111	0,026387
IAELSATAQEIIK	IAELSATAQEIIK; APOB	0,065656	0,079583
STDYGIFQINSR	STDYGIFQINSR; LYZ	0,292861	0,344543
TSCLLFMGR	TSCLLFMGR; SERPIND1	0,352114	0,402416
ILNIFGVIK	ILNIFGVIK; TFRC	0,504434	0,560482
DFALQNPSAVPR	DFALQNPSAVPR; IGFALS	0,734001	0,785476
EAQLPVIENK	EAQLPVIENK; PLG	0,746202	0,785476
GGEGTGYFVDFSVR	GGEGTGYFVDFSVR; HRG	0,823917	0,845043
CQSWSSMTPHR	CQSWSSMTPHR; PLG	0,871443	0,871443

Supplementary Table 5. Statistics result of MRM assay (Cohort 3)

Kendall´s Tau trend test between peptide quantitites and COVID19 treatment escalation score (WHO 3, 4, 5, 6, 7)

Peptide	Peptide.Gene	p.value	adjusted p.value
TFTLLDPK	TFTLLDPK; PGLYRP2	1.6E-16	6.55E-15
GCPDVQASLPDAK	GCPDVQASLPDAK; PGLYRP2	2.73E-16	6.55E-15
ESDTSYVSLK	ESDTSYVSLK; CRP	3.55E-14	5.67E-13
GYSIFSYATK	GYSIFSYATK; CRP	6.79E-14	8.15E-13
CNLLAEK	CNLLAEK; AHSG	3.93E-13	3.43E-12
TVVQPSVGAAAGPVVPPCPGR	TVVQPSVGAAAGPVVPPCPGR; AHSG	4.28E-13	3.43E-12
GDVAFVK	GDVAFVK; TF	4.94E-12	3.39E-11
DSVTGTLPK	DSVTGTLPK; KLKB1	4.6E-10	2.76E-09
WCALSHHER	WCALSHHER; TF	5.44E-10	2.90E-09
AHVDALR	AHVDALR; APOA1	1.1E-09	5.26E-09
ATEHLSTLSEK	ATEHLSTLSEK; APOA1	1.58E-09	6.43E-09
LAELPADALGPLQR	LAELPADALGPLQR; IGFALS	1.61E-09	6.43E-09
DFALQNPSAVPR	DFALQNPSAVPR; IGFALS	7.26E-09	2.68E-08
TINPAVDHCCK	TINPAVDHCCK; AFM	4.23E-08	1.45E-07
EQLSLLDR	EQLSLLDR; SERPINA3	5.51E-08	1.76E-07
WEMPFDPQDTHQSR	WEMPFDPQDTHQSR; SERPINA3	2.4E-07	7.11E-07
HFQNLGK	HFQNLGK; AFM	2.52E-07	7.11E-07
EITALAPSTMK	[illegible]ALAPSTMK; ACTA1;ACTA2;ACTB;ACTC1;ACTG1;ACT[illegible]	4.39E-07	1.17E-06
GHMLENHVER	GHMLENHVER; ITIH1	8.05E-07	2.03E-06
VLDLSCNR	VLDLSCNR; CD14	9.63E-07	2.31E-06
ALDFAVGEYNK	ALDFAVGEYNK; CST3	2.6E-06	5.94E-06
IADAHLDR	IADAHLDR; HRG	0,000003	6.34E-06
VEGTAFVIFGIQDGEQR	VEGTAFVIFGIQDGEQR; C3	3.04E-06	6.34E-06
YAGSQVASTSEVLK	YAGSQVASTSEVLK; VWF	8.28E-06	1.66E-05
ANRPFLVFIR	ANRPFLVFIR; SERPINC1	2.7E-05	5.18E-05
IAYGTQGSSGYSLR	IAYGTQGSSGYSLR; KLKB1	9.19E-05	0,00017
TSCLLFMGR	TSCLLFMGR; SERPIND1	0,00010	0,00018
EAQLPVIENK	EAQLPVIENK; PLG	0,00268	0,00460
IAELSATAQEIIK	IAELSATAQEIIK; APOB	0,00611	0,00995
GGEGTGYFVDFSVR	GGEGTGYFVDFSVR; HRG	0,00622	0,00995
ADQVCINLR	ADQVCINLR; EFEMP1	0,00830	0,01285
AADDTWEPFASGK	AADDTWEPFASGK; TTR	0,01563	0,02345
ILTSDVFQDCNK	ILTSDVFQDCNK; VWF	0,02029	0,02951
LLDSLPSDTR	LLDSLPSDTR; SERPING1	0,03238	0,04571
EQHLFLPFSYK	EQHLFLPFSYK; APOB	0,04849	0,06649
LVLLNAIYLSAK	LVLLNAIYLSAK; SERPING1	0,07269	0,09692
CQSWSSMTPHR	CQSWSSMTPHR; PLG	0,09000	0,11580
GSPAINVAVHVFR	GSPAINVAVHVFR; TTR	0,09168	0,11580
YWCNDGK	YWCNDGK; LYZ	0,10536	0,12968
FNAVLTNPQGDYDTSTGK	FNAVLTNPQGDYDTSTGK; C1QC	0,14629	0,17554
STDYGIFQINSR	STDYGIFQINSR; LYZ	0,32030	0,37498
SDVMYTDWK	SDVMYTDWK; ORM2	0,53354	0,60975
VHQYFNVELIQPGAVK	VHQYFNVELIQPGAVK; C3	0,54707	0,61068

TNQVNSGGVLLR	TNQVNSGGVLLR; C1QC	0,60489	0,65988
GLPNVVTSAISLPNIR	GLPNVVTSAISLPNIR; PRG4	0,64839	0,69161
ILNIFGVIK	ILNIFGVIK; TFRC	0,69961	0,73003
DSGSYFCR	DSGSYFCR; FCGR3A	0,74780	0,76371
VSASPLLYTLIEK	VSASPLLYTLIEK; TFRC	0,82633	0,82633

Supplementary Table 6. Statistics result of MRM assay (Analytical Reproducibility)
Pearson correlation of absolute peptide quantities obtained for samples from cohort 2 in two laboratories, running two different LC-MS/MS platforms

Peptide; Protein	cor (R)	cor (R²)	p.val
AHVDALR; APOA1_HUMAN	1,00	1,00	1.05E-70
GYSIFSYATK; CRP_HUMAN	1,00	1,00	2.62E-67
ATEHLSTLSEK; APOA1_HUMAN	1,00	0,99	1.65E-58
LLDSLPSDTR; SERPING1	1,00	0,99	1.91E-58
EQLSLLDR; SERPINA3	0,99	0,99	1.5E-55
LVLLNAIYLSAK; SERPING1	0,99	0,99	2.52E-54
HFQNLGK; AFM	0,99	0,99	3.1E-54
EAQLPVIENK; PLG	0,99	0,98	8.76E-49
EQHLFLPFSYK; APOB_HUMAN	0,99	0,98	1.01E-48
CNLLAEK; AHSG	0,99	0,98	3.08E-48
SDVMYTDWK; ORM2	0,99	0,98	8.66E-47
GDVAFVK; TRFE_HUMAN	0,99	0,98	3.3E-46
WEMPFDPQDTHQSR; SERPINA3	0,98	0,97	1.23E-42
GSPAINVAVHVFR; TTR	0,98	0,97	1.77E-42
TFTLLDPK; PGLYRP2	0,98	0,96	1.01E-40
YWCNDGK; LYZ	0,97	0,94	3.39E-35
TVVQPSVGAAAGPVVPPCPGR; AHSG	0,97	0,94	1.32E-35
IAELSATAQEIIK; APOB_HUMAN	0,97	0,94	3.24E-35
ANRPFLVFIR; SERPINC1	0,96	0,93	8.17E-33
GLPNVVTSAISLPNIR; PRG4_HUMAN	0,96	0,92	1.79E-32
GHMLENHVER; ITIH1_HUMAN	0,96	0,92	1.81E-32
ALDFAVGEYNK; CST3	0,96	0,92	2.24E-32
AADDTWEPFASGK; TTR	0,96	0,91	9.45E-31
TINPAVDHCCK; AFM	0,92	0,84	8.44E-24
LAELPADALGPLQR; IGFALS	0,91	0,82	1.82E-22
TNQVNSGGVLLR; C1QC_HUMAN	0,91	0,82	3.15E-22
GGEGTGYFVDFSVR; HRG	0,90	0,81	8.94E-22
CQSWSSMTPHR; PLG	0,89	0,79	1.08E-19
ADQVCINLR; EFEMP1	0,88	0,77	1.43E-13
WCALSHHER; TF	0,85	0,72	9.47E-17
TSCLLFMGR; SERPIND1	0,84	0,70	5.62E-16
VHQYFNVELIQPGAVK; CO3_HUMAN	0,80	0,64	6.96E-14
IAYGTQGSSGYSLR; KLKB1_HUMAN	0,80	0,64	7.81E-14
ESDTSYVSLK; CRP_HUMAN	0,74	0,55	4.28E-11
ILTSDVFQDCNK; VWF	0,65	0,42	5.7E-08
FNAVLTNPQGDYDTSTGK; C1QC_HUMAN	0,63	0,39	1.86E-07
DFALQNPSAVPR; IGFALS	0,30	0,09	0,037
GCPDVQASLPDAK; PGLYRP2	0,30	0,09	0,041
STDYGIFQINSR; LYZ	0,26	0,07	0,048
ILNIFGVIK; TFRC	0,21	0,04	0,124

Supplementary Table 7. Recommendations by Mischak et al. 2010 (PMID 20739680) with respect to this study.

Overview	Recommendation by Mishak et al	Our description
Describe and justify the clinical question, outcomes, and selection of subjects	Describe the clinical question and justify why it is of interest; describe what outcomes are assessed and comment on their clinical validity, potential for misclassification, and verification bias, if pertinent; clarify what are the eligibility criteria for the selected study populations and justify specific choices	Addressed. Clinical questions, relevance and outcomes are indicated in introduction, methods and results. Potential limitations, validity and need of further prospective validation is discussed.
Describe the assessed subjects	Provide demographic information with gender, age, ethnic origin, and concomitant medications at a minimum, and all relevant disease-related and clinical parameters.	Addressed. We have provided both a summary of the cohorts, as well as a table stating patient data.
Describe sampling	Provide an accurate description of the sampling conditions and procedures (including the collection process and any manipulation of the sample before storage, the time between sampling and storage, storage conditions, and the addition of any protease inhibitors and/or preservatives). Justify the sampling choices according to the literature or supporting experimental data.	Addressed. The sampling was performed as detailed in the published protocol of the underlying clinical study (Kurth et al. 2020). The time between sampling and acquisition, as well as in-between storage of each respective cohort is stated in the Methods section. EDTA or Citrate was added where indicated. No protease inhibitors were added.
Describe experimental methodology	The procedure, as well as the observed standard deviation of technical specifications related to the procedure, should be given. To attribute the same identity to a certain feature in several independent analyses, accepted deviations of mass and other parameters (retention time, migration, position on gel, etc.) must be reported. Also, the observed deviation in identifying parameters and (relative) abundance, when the same sample is analyzed repeatedly, must be reported.	Addressed. Technical specifications of the assay and their variance are indicated in Supplementary Tables 7, 8. This includes observed deviations in abundance (i.e. the intra- and inter-batch repeatability). Deviations of mass are not applicable to the detection method; the accepted deviations for retention times are reported in Supplementary Tables 2,3.
Describe the statistical evaluation	Provide details on determination of sample size, statistical analysis plan (for appraising calibration, discrimination, and/or reclassification), any consideration or adjustment for covariates (including treatment, whenever pertinent), methods for adjustment for multiplicity, and parameters used in complex machine-learning approaches, whenever pertinent. Clarify which analyses are predefined and which are post hoc.	Addressed in a way that is adapted for our study and research questions and applied methodology. Multiple testing correction was applied as indicated in the methods section, figure captions (according to Benjamini/Hochberg) (Benjamini and Hochberg 1995). For machine learning, all code including parameters used to run the models are provided. All analyses were post hoc.
Validate results	The results must be confirmed in at least one independent sample set. The sampling and characteristics of the validation population should be reported, and the analysis should be symmetrical in the test and validation data sets; any deviations should be reported.	Addressed. Our study contained different cohorts, sampled at different time periods during the pandemic.
Acknowledge limitations	No study is perfect; limitations should be clearly acknowledged and their potential impact on the results discussed.	Addressed. The key limitations:<br>Outcome prediction was not validated in second cohort<br>Models/assay might need adjustment for new variants and treatment options<br>No data on ambulatory patients with COVID-19, as well as asymptomatic and other infectious diseases<br>In general, more data would be required to facilitate the application of more sophisticated statistical tools (especially with respect to machine learning)<br>Additional data would be required on which time-point of the disease is most predictive of outcome
Take responsibility	The contributions of each author should be clearly stated	Author contributions are included at the end of the manuscript.

Supplementary Table 8. Protein/peptide panel composition

Protein Name	Gene name	Protein Uniprot Accesion Number	Corresponding native peptide sequence	Corresponding heavy isotope-labelled internal standard peptide sequence
Proteoglycan 4	PRG4	Q92954	GLPNVVTSAISLPNIR	SILWRGLPNVVTSAISLPNIR(U-13C6,15N4)
Inter-alpha-trypsin inhibitor heavy chain H1	ITIH1	P19827	GHMLENHVER	GHMLENHVER(U-13C6,15N4)LWAYL
Plasminogen, EC 3.4.21.7	PLG	P00747	EAQLPVIENK	AGLLKEAQLPVIENK(U-13C6,15N2)
Plasminogen, EC 3.4.21.7; Apolipoprotein(a)	PLG; LPA	P00747; P08519	CQSWSSMTPHR	CQSWSSMTPHR(U-13C6,15N4)HQK
Actin, aortic smooth muscle; Actin, cytoplasmic 1; Actin, cytoplasmic 2; Actin, gamma-enteric smooth muscle	ACTA2;ACTB;ACTG1;ACTG2	P62736; P60709; P63261; P63267	EITALAPSTMK	MQKEITALAPSTMK(U-13C6,15N2)
Complement C1q subcomponent subunit C	C1QC	P02747	FNAVLTNPQGDYDTSTGK	NSLIRFNAVLTNPQGDYDTSTGK(U-13C6,15N2)
			TNQVNSGGVLLR	TNQVNSGGVLLR(U-13C6,15N4)LQVGE
Cystatin-C	CST3	P01034	ALDFAVGEYNK	ALDFAVGEYNK(U-13C6,15N2)ASNDM
			LVGGPMDASVEEEGVRR	LVGGPMDASVEEEGVR(U-13C6,15N4)RALDFA
Protein ORM2	ORM2	Q06144	SDVMYTDWK	SDVMYTDWK(U-13C6,15N2)KDK
Alpha-1-antichymotrypsin; Filamin-B	SERPINA3; FLNB	P01011; O75369	WEMPFDPQDTHQSR	WEMPFDPQDTHQSR(U-13C6,15N4)FYLSK
Alpha-1-antichymotrypsin	SERPINA3	P01011	EQLSLLDR	EQLSLLDR(U-13C6,15N4)FTEDA
Serotransferrin; Lactotransferrin; Melanotransferrin	TF; LTF; MELTF	P02787; P02788; P08582	GDVAFVK	GDVAFVK(U-13C6,15N2)HSTIF
Serotransferrin	TF	P02787	WCALSHHER	PVKWCALSHHER(U-13C6,15N4)
Apolipoprotein B-100	APOB	P04114	IAELSATAQEIIK	IAELSATAQEIIK(U-13C6,15N2)SQAIA
			EQHLFLPFSYK	EAICKEQHLFLPFSYK(U-13C6,15N2)
EGF-containing fibulin-like extracellular matrix protein 1	EFEMP1	Q12805	ADQVCINLR	ADQVCINLR(U-13C6,15N4)GSFAC
von Willebrand factor	VWF	P04275	ILTSDVFQDCNK	ILTSDVFQDCNK(U-13C6,15N2)LVDPE
			YAGSQVASTSEVLK	YAGSQVASTSEVLK(U-13C6,15N2)YTLFQ
C-reactive protein	CRP	P02741	ESDTSYVSLK	ESDTSYVSLKAPLTK(U-13C6,15N2)
			GYSIFSYATK	GYSIFSYATK(U-13C6,15N2)RQDNE
Lysozyme C, EC 3.2.1.17	LYZ	P61626	STDYGIFQINSR	STDYGIFQINSR(U-13C6,15N4)YWCND
			YWCNDGK	YWCNDGK(U-13C6,15N2)TPGAV
Apolipoprotein A-I	APOA1	F8W696	AHVDALR	AHVDALR(U-13C6,15N4)THLAP
Apolipoprotein A-I	APOA1	P02647	ATEHLSTLSEK	EYHAKATEHLSTLSEK(U-13C6,15N2)
Alpha-2-HS-glycoprotein	AHSG	P02765	TVVQPSVGAAAGPVVPPCPGR	TVVQPSVGAAAGPVVPPCPGR(U-13C6,15N4)IR
			CNLLAEK	CNLLAEK(U-13C6,15N2)QYGFC
Histidine-rich glycoprotein	HRG	P04196	GGEGTGYFVDFSVR	GGEGTGYFVDFSVR(U-13C6,15N4)NCPR
			IADAHLDR	IADAHLDR(U-13C6,15N4)VENTT
N-acetylmuramoyl-L-alanine amidase, EC 3.5.1.28	PGLYRP2	Q96PD5	GCPDVQASLPDAK	PDATKGCPDVQASLPDAK(U-13C6,15N2)
			TFTLLDPK	TFTLLDPK(U-13C6,15N2)ASLLT
Transthyretin	TTR	P02766	GSPAINVAVHVFR	GSPAINVAVHVFR(U-13C6,15N4)KAADD
			AADDTWEPFASGK	AADDTWEPFASGK(U-13C6,15N2)TSESG
Plasma kallikrein, EC 3.4.21.34	KLKB1	P03952	DSVTGTLPK	GCFLKDSVTGTLPK(U-13C6,15N2)
			IAYGTQGSSGYSLR	IAYGTQGSSGYSLR(U-13C6,15N4)LCNTG
Antithrombin-III	SERPINC1	P01008	ANRPFLVFIR	ANRPFLVFIR(U-13C6,15N4)EVPLN
Heparin cofactor 2	SERPIND1	P05546	TSCLLFMGR	TSCLLFMGR(U-13C6,15N4)VANPS
Afamin	AFM	P43652	HFQNLGK	QQECKHFQNLGK(U-13C6,15N2)
			TINPAVDHCCK	TINPAVDHCCK(U-13C6,15N2)TNFAF
Plasma protease C1 inhibitor	SERPING1	P05155	LLDSLPSDTR	LLDSLPSDTR(U-13C6,15N4)LVLLN
			LVLLNAIYLSAK	PSDTRLVLLNAIYLSAK(U-13C6,15N2)
Transferrin receptor protein 1	TFRC	P02786	ILNIFGVIK	ILNIFGVIK(U-13C6,15N2)GFVEP
			VSASPLLYTLIEK	VSASPLLYTLIEK(U-13C6,15N2)TMQNV
Low affinity immunoglobulin gamma Fc region receptor III-A; Low affinity immunoglobulin gamma Fc region receptor III-B	FCGR3A; FCGR3B	P08637; O75015	DSGSYFCR	DSGSYFCR(U-13C6,15N4)GLFGS
Monocyte differentiation antigen CD14	CD14	P08571	VLDLSCNR	VLDLSCNR(U-13C6,15N4)LNR
Insulin-like growth factor-binding protein complex acid labile subunit	IGFALS	P35858	DFALQNPSAVPR	DFALQNPSAVPR(U-13C6,15N4)FVQAI
			LAELPADALGPLQR	LAELPADALGPLQR(U-13C6,15N4)AFWLD
Immunoglobulin heavy variable 5-51; Immunoglobulin heavy variable 1-45; Immunoglobulin heavy variable 5-10-1	IGHV5-51; IGHV1-45; IGHV5-10-1	A0A0C4DH38; A0A0A0MS14; A0A0J9YXX1	ASDTAMYYCAR	WSSLKASDTAMYYCAR(U-13C6,15N4)
Complement C3	C3	P01024	VEGTAFVIFGIQDGEQR	VEGTAFVIFGIQDGEQR(U-13C6,15N4)ISLPE
			VHQYFNVELIQPGAVK	VHQYFNVELIQPGAVK(U-13C6,15N2)VYAYY

Supplementary Table 9. Summary of analytical validation

Summary of analytical validation on 6495C (Agilent) platform. LLOQ - lower limit of quantitation; ULOQ - upper limit of quantification; CV - coefficient of variation. * for 8 peptides (AADDTWEPFASGK, ADQVCINLR, ASDTAMYYCAR, ATEHLSTLSEK, DFALQNPSAVPR, EQLSLLDR, ESDTSYVSLK, IADAHLDR) that differed significant between plasma and BSA matrix, matrix factor (slope plasma/slope BSA x 100%) were calculated and provided

Peptide	Intercept	Slope	R2	LLOQ (ng/ml)	ULOQ (ng/ml)	CV (%) limit	Validation	Low concentration (LLOQ)			Medium concentration (LLOQ+ULOQ)/2			High concentration (ULOQ)			Matrix effects		
								Inter-batch CV (%)	Intra-batch CV (%)	Accuracy (%)	Inter-batch CV (%)	Intra-batch CV (%)	Accuracy (%)	Inter-batch CV (%)	Intra-batch CV (%)	Accuracy (%)	P-value	Matrix effect	Matrix factor (%)
AADDTWEPFASGK	-9.03E-02	4.00E-04	0.9995	573.05	146,700.00	20	Pass	19.2	10.7	90.7	7.2	5.3	98.4	10.0	3.6	94.5	0.004	X	63.3
ADQVCINLR	6.90E-03	7.00E-04	0.9949	8.95	146,700.00	40	Pass	12.5	10.1	96.9	11.2	1.7	97.9	6.5	2.0	97.8	0.026	X	91.2
AHVDALR	-4.30E-03	4.00E-04	0.9985	8.95	146,700.00	20	Pass	2.7	2.0	99.4	2.1	0.3	99.2	4.5	0.3	99.7	0.694	-	
ALDFAVGEYNK	-7.70E-03	3.00E-04	0.9996	143.26	146,700.00	20	Pass	15.2	12.7	89.5	4.4	3.8	97.8	5.4	2.8	94.4	0.584	-	
ANRPFLVFIR	3.70E-03	5.00E-04	0.9995	8.95	146,700.00	20	Pass	10.7	8.5	87.6	2.9	1.5	97.7	5.0	1.6	99.3	0.414	-	
ASDTAMYYCAR	-1.89E-01	1.00E-04	0.9923	2,292.19	146,700.00	20	Pass	18.3	9.6	90.4	11.8	7.0	94.7	11.7	3.4	93.2	0.006	X	69.2
ATEHLSTLSEK	-1.02E-02	3.00E-04	0.9997	35.82	146,700.00	20	Pass	17.5	4.7	93.9	2.0	1.4	99.1	5.4	0.8	97.5	0.018	X	95.8
CNLLAEK	-2.50E-03	1.00E-04	0.9995	2,292.19	146,700.00	20	Pass	11.3	2.8	99.1	11.3	1.3	97.6	7.6	2.5	96.7	0.635	-	
CQSWSSMTPHR	-1.31E-01	3.00E-04	0.9997	2,292.19	146,700.00	20	Pass	18.4	7.4	95.6	19.2	9.2	88.7	6.5	5.1	98.0	0.690	-	
DFALQNPSAVPR	-6.83E-02	1.70E-03	1.0000	573.05	36,675.00	20	Pass	11.1	5.4	95.9	8.9	4.7	94.9	6.6	2.8	96.3	0.008	X	267.8
DSGSYFCR	-2.55E-02	6.00E-04	0.9985	143.26	2,292.19	20	Pass	15.7	5.6	96.9	9.8	2.3	97.3	10.6	1.3	98.2	0.480	-	
DSVTGTLPK	-6.00E-03	9.00E-04	0.9993	8.95	36,675.00	40	Pass	18.3	6.4	93.8	5.7	1.6	97.8	5.1	1.4	98.7	0.982	-	
EAQLPVIENK	8.00E-03	2.00E-04	0.9997	2.24	146,700.00	20	Pass	19.5	4.2	90.5	2.7	0.8	98.3	1.8	0.9	99.8	0.433	-	
EITALAPSTMK	-6.45E-02	2.00E-04	0.9982	573.05	146,700.00	40	Pass	3.2	3.0	96.7	2.5	1.9	98.5	17.9	1.8	98.6	0.075	-	
EQHLFLPFSYK	-1.84E-02	2.00E-04	1.0000	573.05	36,675.00	20	Pass	6.8	4.6	93.6	4.0	2.0	98.8	4.4	1.7	98.4	0.462	-	
EQLSLLDR	-2.41E-02	6.00E-04	1.0000	573.05	36,675.00	20	Pass	6.8	4.2	94.4	5.2	3.4	96.4	4.5	0.9	98.2	0.017	X	182.4
ESDTSYVSLK	-5.07E-02	9.00E-04	1.0000	143.26	36,675.00	20	Pass	17.2	4.7	94.1	7.7	1.0	98.8	4.2	1.7	99.1	0.015	X	170.7
FNAVLTNPQGDYDTSTGK	1.10E-03	2.00E-04	0.9987	2,292.19	146,700.00	20	Pass	28.0	11.5	91.1	15.3	12.7	94.0	12.1	6.3	95.3	0.380	-	
GCPDVQASLPDAK	-5.67E-02	8.00E-04	0.9962	143.26	146,700.00	40	Pass	25.5	3.7	95.8	19.4	2.0	98.7	32.3	2.1	97.2	0.527	-	
GDVAFVK	-7.90E-03	2.00E-04	0.9996	35.82	146,700.00	20	Pass	20.3	6.9	87.5	1.9	1.2	99.9	6.6	1.0	97.8	0.102		
GGEGTGYFVDFSVR	-1.15E-01	9.00E-04	0.9999	573.05	36,675.00	20	Pass	17.0	7.4	92.0	12.1	7.6	91.1	14.5	6.8	91.1	0.093	-	
GHMLENHVER	-8.71E-02	1.00E-04	0.9999	2,292.19	146,700.00	20	Pass	13.3	1.1	99.3	1.7	0.4	98.8	4.9	0.7	99.1	0.909	-	
GLPNVVTSAISLPNIR	-9.60E-03	4.00E-04	0.9997	35.82	146,700.00	20	Pass	29.8	4.1	96.0	36.5	1.3	96.9	38.5	0.4	99.5	0.460	-	
GSPAINVAVHVFR	-6.21E-02	2.00E-04	0.9939	573.05	146,700.00	40	Pass	24.6	7.6	79.6	16.5	2.0	87.8	19.6	5.1	79.3	0.969	-	
GYSIFSYATK	-3.77E-02	4.00E-04	0.9999	143.26	146,700.00	20	Pass	8.1	3.4	98.4	3.1	1.9	98.7	5.8	1.4	99.6	0.743	-	
HFQNLGK	-1.40E-01	4.00E-04	0.9914	573.05	146,700.00	40	Pass	29.6	1.5	96.4	21.8	1.3	98.0	3.3	0.8	98.8	0.329	-	
IADAHLDR	-1.36E-02	1.60E-03	0.9973	8.95	36,675.00	40	Pass	9.3	3.7	96.7	7.3	0.4	97.9	3.5	1.1	99.0	0.007	X	200.0
IAELSATAQEIIK	1.29E-02	3.00E-04	0.9996	2.24	146,700.00	40	Pass	12.2	5.7	92.8	7.5	3.5	97.0	7.5	2.6	98.1	0.582	-	
IAYGTQGSSGYSLR	-1.59E-01	1.40E-03	0.9996	573.05	146,700.00	20	Pass	11.6	8.8	97.4	7.4	7.1	94.1	12.7	7.1	85.3	0.286	-	
ILNIFGVIK	-1.50E-03	6.00E-04	0.9992	2.24	36,675.00	20	Pass	34.2	8.1	97.9	42.1	0.5	99.8	38.7	0.7	97.9	0.238	-	
ILTSDVFQDCNK	not pass							85.8	5.3	94.5	25.9	5.1	97.0	41.6	9.6	76.9	0.061	-	
LAELPADALGPLQR	-2.93E-02	1.19E-03	0.9275	8.95	146,700.00	20	Pass	10.9	3.3	95.5	8.8	1.4	95.1	12.5	1.7	96.5	0.079	-	
LLDSLPSDTR	-1.62E-03	2.85E-04	0.9998	8.95	146,700.00	20	Pass	14.2	8.5	88.6	3.6	1.7	98.7	4.1	0.9	98.9	0.173	-	
LVGGPMDASVEEEGVRR	not pass							82.4	19.9	75.7	44.1	27.8	87.5	29.0	20.1	75.4	0.438	-	
LVLLNAIYLSAK	-9.88E-03	2.43E-04	0.9975	35.82	146,700.00	20	Pass	80.6	7.0	76.4	70.8	1.7	97.2	71.4	1.6	98.3	0.163	-	
SDVMYTDWK	-2.41E-01	7.31E-04	0.9993	573.05	146,700.00	20	Pass	18.6	6.7	93.9	4.5	3.8	96.5	11.8	2.2	97.4	0.162	-	
STDYGIFQINSR	-5.26E-03	1.26E-04	1.0000	143.26	146,700.00	20	Pass	14.3	10.7	85.7	6.5	1.6	98.7	5.6	2.3	98.4	0.578	-	
TFTLLDPK	-1.33E-02	1.17E-04	0.9999	573.05	146,700.00	20	Pass	4.8	3.4	98.7	3.8	1.5	99.4	5.7	2.4	97.1	0.108	-	
TINPAVDHCCK	-1.23E-01	1.52E-03	0.9995	143.26	146,700.00	40	Pass	22.8	3.1	97.8	36.4	1.6	98.4	20.5	1.8	98.5	0.221	-	
TNQVNSGGVLLR	-1.55E-02	4.57E-04	0.9991	143.26	146,700.00	20	Pass	7.4	2.1	96.8	6.4	3.1	99.1	8.8	1.9	98.4	0.148	-	
TSCLLFMGR	-2.85E-01	5.56E-04	0.9999	2,292.19	146,700.00	20	Pass	15.5	1.3	99.3	10.4	2.1	97.8	7.5	1.6	99.0	0.787	-	
TVVQPSVGAAAGPVVPPCPGR	1.03E-01	1.59E-03	0.9981	573.05	146,700.00	20	Pass	10.2	3.0	93.7	9.5	2.0	99.2	10.1	4.9	96.3	0.439	-	
VEGTAFVIFGIQDGEQR	not pass							20.3	2.5	94.5	17.0	3.8	89.0	25.4	3.3	98.3	0.439	-	
VHQYFNVELIQPGAVK	-4.27E-02	4.35E-04	0.9990	73.06	1,197,000.00	40	Pass	45.2	3.9	87.8	30.4	1.8	98.4	25.8	3.8	95.1	0.132	-	
VLDLSCNR	1.13E-03	7.06E-04	0.9998	2.24	146,700.00	20	Pass	10.7	4.5	94.4	7.2	0.8	99.6	15.8	0.8	99.7	0.506	-	
VSASPLLYTLIEK	-1.26E-02	1.07E-03	0.9989	8.95	146,700.00	20	Pass	11.1	4.1	96.4	5.9	2.2	97.6	9.0	1.1	99.1	0.476	-	
WCALSHHER	-1.22E-01	5.46E-04	0.9998	573.05	146,700.00	20	Pass	15.0	1.8	99.2	10.8	1.0	99.6	12.6	1.0	97.7	0.342	-	
WEMPFDPQDTHQSR	-5.11E-02	1.38E-04	0.9996	2,292.19	146,700.00	20	Pass	11.2	3.0	93.0	7.6	2.6	95.1	6.2	2.0	97.7	0.540	-	
YAGSQVASTSEVLK	-1.77E-02	3.49E-04	0.9995	143.26	146,700.00	20	Pass	16.1	13.5	91.2	5.6	4.2	95.8	6.1	3.2	96.1	0.412	-	
YWCNDGK	-6.61E-01	3.32E-03	0.9971	573.05	36,675.00	20	Pass	8.5	1.2	97.7	19.8	1.0	98.7	10.9	1.5	99.0	0.086	-	
**Median**			**0.99949**	**143.26**	**146,700.00**	**-**	**-**	**15.3**	**4.6**	**94.5**	**7.6**	**1.9**	**97.9**	**8.2**	**1.8**	**98.0**	**-**	**-**	**-**

Supplementary Table 10. Quantification of pooled COVID-19 samples

Quantification of pooled COVID-19 samples. CV - coefficient of variation. Median Conc - median of the calculated concentration from 5 replicates

* Median Conc - Shown numbers refers to contained in measured pooled sample

Peptide	WHO3 (n = 5)		WHO4 (n = 5)		WHO5 (n = 5)		WHO6 (n = 5)		WHO7 (n = 5)	
	CV (%)	Median Conc (ng/ml)*	CV (%)	Median Conc (ng/ml)*	CV (%)	Median Conc (ng/ml)*	CV (%)	Median Conc (ng/ml)*	CV (%)	Median Conc (ng/ml)*
AADDTWEPFASGK	29.4	395.0	42.9	300.8	56.9	273.5	26.6	306.5	36.8	302.6
ADQVCINLR	8.0	35.9	11.2	52.8	8.8	39.4	5.9	20.8	8.7	23.5
AHVDALR	0.9	9420.8	0.3	12517.8	0.3	10212.8	0.3	7023.9	0.2	4178.3
ALDFAVGEYNK	29.3	280.5	19.1	138.3	23.3	178.1	9.9	115.8	14.8	123.6
ANRPFLVFIR	3.8	246.4	1.2	1419.8	1.5	1235.2	1.5	700.8	2.6	691.2
ASDTAMYYCAR	76.0	1536.7	34.0	1518.3	96.0	1504.4	41.3	1512.2	20.7	1496.4
ATEHLSTLSEK	1.0	18103.5	1.8	18717.1	0.6	15641.4	1.7	10797.2	1.2	6031.0
CNLLAEK	11.2	5531.4	5.3	14792.8	10.4	8972.2	3.9	7021.8	3.0	4634.4
CQSWSSMTPHR	8.2	6162.7	12.7	5419.8	11.5	6959.7	18.5	2912.8	12.7	2326.6
DFALQNPSAVPR	35.0	247.9	34.1	157.8	28.6	95.6	63.7	96.9	29.2	50.1
DSGSYFCR	16.0	53.4	12.2	83.3	22.5	60.3	19.6	49.1	22.7	48.7
DSVTGTLPK	5.5	305.1	2.0	158.8	6.4	90.9	4.1	63.5	4.6	37.1
EAQLPVIENK	4.2	444.5	2.7	1234.7	3.2	1086.9	2.0	659.8	1.3	414.5
EITALAPSTMK	22.9	795.9	18.6	347.5	21.8	351.2	17.0	310.3	11.9	331.3
EQHLFLPFSYK	24.8	1067.9	5.6	3280.5	6.0	2322.8	5.5	2055.8	3.3	2085.0
EQLSLLDR	23.0	362.2	3.6	1495.8	12.1	770.7	6.5	716.9	3.1	1806.8
ESDTSYVSLK	14.8	610.2	10.6	990.2	10.9	978.6	7.1	566.5	6.2	1782.9
FNAVLTNPQGDYDTSTGK	66.5	10.1	36.7	235.7	57.6	81.3	50.9	132.7	23.5	578.2
GCPDVQASLPDAK	71.2	363.2	23.4	173.8	70.0	112.6	41.4	101.1	45.1	83.1
GDVAFVK	3.5	145.9	2.8	1206.9	1.6	505.2	2.5	528.6	1.2	1111.2
GGEGTGYFVDFSVR	21.5	531.3	13.6	1439.0	6.7	2011.1	14.1	754.9	19.9	744.4
GHMLENHVER	3.9	3708.9	1.3	6478.8	1.0	4800.6	1.2	3809.8	1.7	4843.1
GLPNVVTSAISLPNIR	15.2	55.6	9.0	99.8	10.4	69.1	6.4	68.8	6.9	71.2
GSPAINVAVHVFR	33.5	427.6	14.7	777.7	26.1	573.4	8.4	748.3	5.0	1313.6
GYSIFSYATK	20.2	113.8	4.3	243.2	19.0	160.4	7.8	182.8	4.0	1176.8
HFQNLGK	16.3	363.4	0.3	453.1	4.1	387.5	2.5	380.1	1.7	383.2
IADAHLDR	7.2	444.7	3.6	156.2	9.8	52.2	5.4	42.6	4.3	27.3
IAELSATAQEIIK	13.8	8.1	12.3	48.5	23.5	13.5	9.6	13.3	12.5	42.6
IAYGTQGSSGYSLR	41.4	180.9	23.8	264.6	7.0	243.3	22.5	194.3	28.9	155.8
ILNIFGVIK	14.3	15.2	7.4	13.1	5.6	8.4	5.0	6.2	8.9	6.4
LAELPADALGPLQR	4.4	37.4	9.2	57.9	9.0	36.3	10.1	33.9	5.1	33.9
LLDSLPSDTR	8.7	132.4	2.6	567.7	2.8	727.4	1.8	376.4	2.4	869.8
LVLLNAIYLSAK	21.0	665.2	3.4	1849.5	1.9	2383.9	2.8	1254.9	3.1	2879.2
SDVMYTDWK	21.2	1525.3	12.5	2262.2	9.2	3591.9	7.5	1547.9	8.3	1512.1
STDYGIFQINSR	10.9	1976.2	32.8	227.9	23.1	163.9	27.4	199.0	10.3	221.7
TFTLLDPK	13.2	262.6	9.4	506.1	7.2	414.3	6.1	263.6	9.2	201.3
TINPAVDHCCK	9.9	200.8	3.3	415.8	3.7	329.7	4.2	283.8	2.7	253.0
TNQVNSGGVLLR	30.8	50.1	4.7	266.4	7.7	145.9	7.5	191.7	6.0	298.8
TSCLLFMGR	20.3	527.1	22.4	531.0	19.6	519.9	29.8	518.2	36.9	520.6
TVVQPSVGAAAGPVVPPCPGR	6.1	2589.0	8.0	1536.9	11.2	1185.5	6.6	758.3	10.2	353.3
VHQYFNVELIQPGAVK	7.4	546.3	5.4	2016.2	3.8	793.9	3.3	1780.3	2.2	3853.7
VLDLSCNR	7.4	142.8	2.7	59.1	5.1	31.2	4.8	16.9	3.6	16.8
VSASPLLYTLIEK	18.8	33.8	11.7	31.6	7.9	19.1	16.0	19.3	2.9	18.5
WCALSHHER	4.4	3177.0	1.1	5273.1	1.8	3818.4	2.1	2509.7	2.0	2326.8
WEMPFDPQDTHQSR	19.4	898.0	11.1	1485.9	9.2	1310.6	7.7	987.5	5.9	1876.0
YAGSQVASTSEVLK	108.0	94.6	34.4	90.9	22.7	119.6	40.4	67.6	9.4	91.4
YWCNDGK	6.3	405.9	10.3	291.7	13.1	236.4	14.2	212.0	15.3	207.6

Supplementary Table 11. Meta Data for Cohort 3.

Meta Data for Cohort 3. CCI - Charlson Comorbidity Index; SOFA - Sequential Organ Failure Assessment; APACHEII - Acute Physiology And Chronic Health Evaluation; ABCS - age, biomarkers, clinical history, sex mortality risk score; BMI - Body mass index; DNR - do-not-resuscitate; DNI - do-not-intubate

PatientID_anon	SampleID_anon	days_since_sy	ample_days_to_outcom	Hospitalization_days	Sample_WHO	Patient_WHO_max	CCI	SOFA	APACHEII	ABCS_first	ABCS_admission	Sex	Age	BMI	Died	DNR/DNI	Mechanical_ventilation	ECMO	Dialysis	ethasone_tre	Remdesivir
P001	S001	47	41	65	7	7	3	6	25	0,5	0,09	Male	63	36,23	0	0	1	0	1	0	0
P002	S002	27	64	87	7	7	4	10	17	0,2	0,09	Female	80	25,65	0	0	1	0	1	0	0
P003	S003	26	1	21	7	8	3	12	30	0,6	0,9	Male	71	25,65	1	0	1	1	1	0	0
P004	S004	20	31	48	7	8	5	13	28	0,8	0,09	Male	74	29,22	1	0	1	1	1	0	0
P005	S005		6	24	7	8	7	14	29	0,2		Female	50	23,88	1	0	1	0	0	0	0
P006	S006	17	85	101	6	7	1	5	17	0,2	0,09	Female	35	36,73	0	0	1	0	0	0	0
P007	S007	23	28	38	5	5	5			0,09	0,09	Male	79	16,33	0	0	0	0	0	0	0
P008	S008	19	4	17	6	7	1	1,5	11	0,09	0,09	Female	55	36,41	0	0	1	0	0	0	0
P009	S009	21	28	42	7	7	0	7,5	27	0,02	0,3	Male	26	24,49	0	0	1	1	0	0	0
P010	S010	16	6	16	6	8	4	8	17	0,8	0,09	Male	86	23,66	1	1	1	0	1	0	0
P011	S011	14	208	220	7	8	1	11,5		0,6	0,6	Male	54	27,76	1	0	1	1	1	1	0
P012	S012	13	16	25	7	7	3	10	20	0,4	0,2	Male	73	27,36	0	0	1	0	0	0	0
P013	S013	19	1	6	3	3	4			0	0,02	Female	71	32,87	0	0	0	0	0	0	0
P014	S014	14	6	12	3	5	0			0	0,02	Male	21	33,21	0	0	0	0	0	0	0
P015	S015	16	13	15	4	4	4			0,02	0,02	Male	71	30,12	0	0	0	0	0	0	0
P016	S016		6	17	4	5	1	3	8	0		Male	52	28,73	0	0	0	0	0	0	0
P017	S017	39	32	66	4	7	0	0		0,02		Female	42	29,38	0	0	1	0	0	0	0
P018	S018	26	48	72	7	7	3	4	14	0,09	0,09	Female	60	31,25	0	0	1	0	0	0	0
P019	S019	14	20	33	6	7	7	10	34	0,8	0,4	Male	69	34,11	0	0	1	0	1	1	0
P020	S020	26	16	41	6	7	0	5	13	0,09	0,3	Male	40	41,52	0	0	1	0	0	0	0
P021	S021	29	69	92	7	8	2	12	37	0,2		Male	42	29,38	1	0	1	1	0	1	0
P022	S022	6	12	15	4	8	3	0	12	0,09	0,4	Male	53	20,78	1	0	1	1	1	1	0
P023	S023	8	12	15	4	4	0			0,2	0,02	Male	39	26,58	0	0	0	0	0	1	0
P024	S024		2	7	3	3	1			0,02	0,02	Male	30	30,8	0	0	0	0	0	1	0
P025	S025	10	1	3	3	3	1			0	0	Male	61	23,04	0	0	0	0	0	0	0
P026	S026	9	24	28	4	4	1			0,02	0,02	Male	56	21,2	0	0	0	0	0	1	0
P027	S027	10	1	7	3	4	1			0,09	0,09	Male	54	24,48999977	0	0	0	0	0	1	0
P028	S028	26	21	32	6	6	0	2	33	0,02	0,09	Male	48	35,22000122	0	0	1	0	0	1	0
P029	S029	13	4	7	3	3	2			0,02	0,2	Male	59	25,95000076	0	0	0	0	0	0	0
P030	S030	53	40	68	6	8	3	7	19	0,3		Male	64	30,86000061	1	0	1	0	1	0	0
P031	S031	23	24	45	7	8	3	14	31	0,6	0,2	Male	48	32,65000153	1	0	1	1	1	0	0
P032	S032	2	4	6	4	4	0			0,02	0,02	Male	25	27,76000023	0	0	0	0	0	0	0
P033	S033	20	172	192	7	7	1	8	32	0,09		Male	59	24,69000053	0	0	1	1	0	1	0
P034	S034	11	23	32	3	3				0,02	0,02	Male	55	23,18000031	0	0	0	0	0	1	0
P035	S035		3	6	3	3	0			0	0	Female	21	23,87999916	0	0	0	0	0	0	0
P036	S036	13	4	6	4	4	1			0,02	0,02	Male	51	25,73999977	0	0	0	0	0	1	0
P037	S037	7	42	44	4	8	3	5	16	0,2	0,2	Female	69	30,10000038	1	1	1	0	1	1	0
P038	S038	16	8	10	4	4	1			0,09	0,02	Male	55	26,17000008	0	0	0	0	0	1	0
P039	S039	2	2	3	3	3	0			0,02	0	Male	26	23,14999962	0	0	0	0	0	0	0
P040	S040	16	19	28	6	8	6	13	37	0,2	0,3	Male	66	30,86000061	1	0	1	0	1	1	0
P041	S041	5	10	11	3	3	0			0	0	Female	43	30,47999954	0	0	0	0	0	0	0
P042	S042	14	14	16	5	5	3	0	14	0,3	0,09	Male	78	24,79999924	0	0	0	0	0	1	1
P043	S043	8	7	11	7	7	0	7	19	0,3	0,3	Male	46	29,97999954	0	0	1	0	0	1	0
P044	S044	16	7	10	4	4	2			0,3	0,09	Male	66		0	0	0	0	0	1	0
P045	S045	7	13	14	4	4	1			0,09	0,09	Female	53	31,15999985	0	0	0	0	0	1	1
P046	S046	19	33	47	7	8	1	10	23	0,09	0,5	Male	54	29,38999939	1	0	1	1	1	1	1
P047	S047	22	1	12	3	5	3	0		0,02	0,09	Female	77		0	0	0	0	0	1	0
P048	S048	24	12	33	5	8	5			0,09	0,09	Female	82	29,03000069	1	0	1	0	0	1	0
P049	S049	15	41	48	7	7	3	8	27	0,09	0,09	Male	67	24,48979592	0	0	1	1	0	1	0
P050	S050	15	47	58	7	8	3	13	42	0,5		Male	78	24,48979592	1	0	1	1	1	1	0
P051	S051	11	7	13	4	5	2	0		0	0,02	Male	60	30,45765595	0	0	0	0	0	1	0
P052	S052	11	14	20	7	7	2	6	15	0,2	0,09	Male	52	32,60030313	0	0	1	0	0	1	1
P053	S053		15	25	7	8	3	10	33	0,3		Male	70	24,69000053	1	0	1	1	1	1	0

P054	S054	13	7	10	4	4	2			0,09	0,02	Male	55	33,24000168	0	0	0	0	0	1	0
P055	S055	11	16	22	6	7	3	5	16	0,2	0,6	Male	78	24,48999977	0	0	1	0	0	1	1
P056	S056		6	10	3	3	0			0,09	0,09	Male	47	24,1516725	0	0	0	0	0	0	0
P057	S057	14	51	55	7	7	3	11	27	0,5	0,3	Male	62		0	0	1	0	1	1	0
P058	S058		1	3	3	3	0					Male	43	20,98999977	0	0	0	0	0	0	0
P059	S059	16	35	45	5	5	4	4	11	0,09	0,09	Male	71	33,20477502	0	0	0	0	0	0	0
P060	S060	19	4	14	6	8	5	7	21	0,9	0,09	Male	81	32,47000122	1	1	1	1	0	1	0
P061	S061		5	7	3	3	1			0,02	0,09	Male	56	22,40878677	0	0	0	0	0	0	0
P062	S062	10	12	12	5	5	1	0	10	0,02	0,02	Female	42	35,38000107	0	0	0	0	0	1	0
P063	S063	6	6	8	4	4	2			0,02	0,02	Male	49	26,80999947	0	0	0	0	0	1	0
P064	S064	3	13	14	5	5	3	1	14	0,09	0,09	Male	78	23,59000015	0	0	0	0	0	1	1
P065	S065		10	15	3	3	3			0,02	0,02	Female	68	24,15999985	0	0	0	0	0	0	0
P066	S066	8	7	13	4	5	3	0	10	0,4	0,09	Male	55	34,02000046	0	0	0	0	0	1	1
P067	S067	14	5	9	4	5	4			0,09	0,3	Male	75	29,62999916	0	0	0	0	0	1	0
P068	S068	13	7	10	4	4	3			0,09	0,09	Male	75	33,22000122	0	0	0	0	0	1	0
P069	S069	10	5	7	4	4	1			0,09	0,09	Male	54	24,33227705	0	0	0	0	0	1	0
P070	S070	13	7	9	3	3	0			0,02	0,02	Female	20	32,46219592	0	0	0	0	0	0	0
P071	S071	10	4	5	3	3	4			0,09	0,09	Male	63	25,51000023	0	0	0	0	0	1	0
P072	S072	9	4	10	3	3	2			0	0	Male	50	30,86000061	0	0	0	0	0	0	0
P073	S073	17	14	22	6	6	0	6	23	0,02	0,2	Male	44		0	0	1	0	0	1	0
P074	S074	5	6	10	4	4	2			0,02	0,02	Male	57	24,89999962	0	0	0	0	0	1	1
P075	S075	8	37	40	5	5	1	0	12	0,09	0,09	Male	58	28,37000084	0	0	0	0	0	1	0
P076	S076	11	2	5	4	4	1			0	0,02	Male	30	32,97999954	0	0	0	0	0	1	0
P077	S077		3	5	3	3	3	2	14	0	0	Male	57	24,69000053	0	0	0	0	0	0	0
P078	S078	6	6	11	4	5	2			0,02	0,02	Male	59	22,53000069	0	0	0	0	0	1	1
P079	S079	14	0	6	3	4	1			0	0	Female	57		0	0	0	0	0	1	0
P080	S080	4	14	17	5	5				0,09	0,02	Male	25	23,65999985	0	0	0	0	0	1	0
P081	S081	4	8	10	5	5	1	3		0,09	0,09	Male	48		0	0	0	0	0	1	1
P082	S082	14	8	17	5	5	2	3	17	0,02		Male	61		0	0	0	0	0	0	0
P083	S083	8	6	10	3	3	1			0,02	0,02	Male	52		0	0	0	0	0	0	0
P084	S084	13	1	7	3	4	0	0	11	0,09	0,02	Male	47	24,39105884	0	0	0	0	0	1	1
P085	S085	14	3	11	4	5	0	0	8	0,02		Male	48	30,36734694	0	0	0	0	0	1	0
P086	S086	18	10	12	4	4	0	2	6	0,02	0	Male	29	38,29999924	0	0	0	0	0	1	0
P087	S087	16	11	21	5	5	4	4	15	0,09	0,09	Male	75	31,4359113	0	0	0	0	0	1	0
P088	S088	13	9	10	5	5	0	0	9	0,02	0,02	Male	30	24,69000053	0	0	0	0	0	1	1
P089	S089	17	1	5	3	4	1			0	0,02	Female	58	30,07999992	0	0	0	0	0	1	0
P090	S090	5	24	28	4	4	5			0,02	0	Female	88	27,88518739	0	0	0	0	0	0	0
P091	S091	9	5	7	5	5	4			0,3	0,2	Male	66	31,13999939	0	0	0	0	0	1	1
P092	S092	4	20	21	3	4	4			0,2	0,2	Male	61	21,30999947	0	0	0	0	0	1	0
P093	S093	7	52	58	7	8						Male	53	29,38999939	1	0	1	1	0	1	0
P094	S094		15	21	5	8	5	3		0,09		Female	80	28,88999939	1	1	0	0	0	1	1
P095	S095		5	10	3	3	9			0,09	0,09	Male	58		0	0	0	0	0	0	0
P096	S096		1	4	3	4	5			0	0	Female	81		0	0	0	0	0	0	0
P097	S097	7	11	12	4	4	3			0,09	0,09	Male	61	29,35000038	0	0	0	0	0	1	0
P098	S098		29	34	5	8	5	4	10	0,7	0,5	Male	62		1	0	1	0	1	1	0
P099	S099	18	5	12	4	5	1	0		0,02	0,02	Male	39	35,06000137	0	0	0	0	0	1	0
P100	S100	6	8	10	5	5	3	1	12	0,09	0,02	Male	61		0	0	0	0	0	1	0
P101	S101	16	27	32	7	7	0	9	20	0,02	0,3	Male	44	26,29999924	0	0	1	0	0	1	0
P102	S102	2	9	14	5	8	4	5		0,9	0,2	Female	79		1	1	0	0	0	1	0
P103	S103	9	12	17	5	5	2	3		0,02	0,09	Male	69		0	0	0	0	0	1	0
P104	S104	9	49	52	7	7	2	10	38	0,2	0,09	Male	57	29,38999939	0	0	1	0	1	1	0
P105	S105	17	4	7	3	3	5			0,02	0,02	Male	70	25,82999992	0	0	0	0	0	0	0
P106	S106	8	6	16	4	5	7	2	11	0,09		Female	63	37,18000031	0	0	0	0	0	1	0
P107	S107	6	16	21	3	5	3	0		0,02	0,02	Female	63	28,03000069	0	0	0	0	0	0	0
P108	S108	13	4	17	4	5	2			0,02		Female	63	31,63999939	0	0	0	0	0	1	0
P109	S109	9	10	12	7	8	3	11	27	0,2	0,09	Female	66	31,25	1	1	1	0	1	1	0

P110	S110	13	41	44	5	5	3	0	11	0,3	0,02	Male	61	24,18704649	0	0	0	0	0	1	0
P111	S111		3	5	4	4	1			0,09	0,02	Male	58	23,77000046	0	0	0	0	0	1	0
P112	S112	11	22	29	4	4	3			0,2	0,2	Male	51	23,66143862	0	0	0	0	0	0	0
P113	S113	18	19	30	6	7	1	3	27	0,2		Male	55	27,70000076	0	0	1	0	0	1	0
P114	S114	26	76	96	7	7	3	14	28	0,6		Male	75	26,12000084	0	0	1	0	1	1	0
P115	S115	9	2	7	4	4	2			0,02	0,09	Male	61	32,40999985	0	0	0	0	0	1	0
P116	S116	6	8	11	5	5	2	0	17	0,02	0,09	Male	58	34,47999954	0	0	0	0	0	1	1
P117	S117	17	18	34	7	8	1	12	34	0,2		Male	57	30,07999992	1	0	1	1	1	1	0
P118	S118	9	32	36	7	8	3	11	28	0,7	0,6	Male	64	30,86000061	1	1	1	1	1	1	0
P119	S119	6	64	65	4	8	2	12	24	0,02	0,09	Male	62	23,14999962	1	1	1	1	1	1	0
P120	S120	8	3	4	4	4	0			0,02	0,02	Male	49	23,79999924	0	0	0	0	0	1	0
P121	S121	14	38	45	5	5	3	3		0,09	0,02	Female	69	29,666548	0	0	0	0	0	1	0
P122	S122	22	25	45	6	7				0,6		Male	73	21,85000038	0	0	1	0	0	1	0
P123	S123	13	56	62	7	8	3	9		0,5		Male	65	33,20000076	1	0	1	0	1	1	0
P124	S124	10	13	19	5	5	2	4	15	0,3		Male	69		0	0	0	0	0	1	0
P125	S125		6	10	3	3	6			0,09	0,02	Female	45		0	0	0	0	0	0	0
P126	S126	17	87	94	7	7	2	9		0,6	0,6	Male	54	53,97999954	0	0	1	0	1	1	0
P127	S127		25	32	7	8	5	13		0,6	0,6	Male	67	29,41176471	1	1	1	1	0	1	0
P128	S128	8	7	9	4	4	3			0,3	0,09	Male	54	31,34693878	0	0	0	0	0	1	0
P129	S129	14	27	32	7	8	2	12		0,2	0,09	Male	69	29,37999916	1	1	1	0	1	1	0
P130	S130	3	14	17	4	8	4	1		0,09	0,2	Male	88		1	1	0	0	0	1	0
P131	S131	8	3	8	6	8		12	28	0,5	0,3	Female	61	54	1	0	1	0	0	1	0
P132	S132	4	35	40	6	7	0	7	12	0	0	Female	22	56,02000046	0	0	1	0	0	1	0
P133	S133	6	4	7	3	3	2			0	0	Female	18	26,62000084	0	0	0	0	0	0	0
P134	S134	10	4	7	4	4						Male	72	25,07999992	0	0	0	0	0	1	0
P135	S135	11	4	7	4	4	5			0,4	0,2	Male	80		0	0	0	0	0	1	0
P136	S136	14	13	24	5	5	6	7	23	0,3	0,2	Female	75		0	0	0	0	1	1	0
P137	S137	10	13	17	5	5	4	3		0,09	0,09	Male	70		0	0	0	0	0	1	0
P138	S138	9	8	13	5	5	2	2		0,09	0,8	Male	60		0	0	0	0	0	1	0
P139	S139	9	6	11	5	5	5	4		0,5	0,2	Male	81		0	0	0	0	0	1	0
P140	S140	10	76	79	7	7	7	9	26	0,8	0,5	Male	82	36,84999847	0	0	1	0	0	1	0
P141	S141	13	5	10	3	3	3			0	0,02	Male	41	31,48148148	0	0	0	0	0	0	0
P142	S142	6	8	9	4	4	2			0,02	0,02	Male	66	33,56999969	0	0	0	0	0	1	0
P143	S143	21	10	31	5	8	4			0,6	0,5	Female	94	24,9107674	1	1	0	0	0	1	0
P144	S144	16	20	30	7	7	4	8	20	0,2		Male	78	26,29999924	0	0	1	0	1	1	0
P145	S145	11	11	17	4	4				0,02	0,02	Female	79	34,59999847	0	0	0	0	0	1	0
P146	S146		20	38	3	4	4			0,09		Female	89		0	0	0	0	0	1	0
P147	S147	11	3	11	3	4	3			0	0,02	Female	62		0	0	0	0	0	1	0
P148	S148	11	3	10	4	4	1			0,02	0,09	Male	55	27,46999931	0	0	0	0	0	1	0
P149	S149	12	0	7	3	4	2			0,2	0,02	Male	62	28,29000092	0	0	0	0	0	1	0
P150	S150	18	5	11	4	4	3			0,02	0,09	Female	76	31,25	0	0	0	0	0	0	0
P151	S151	8	47	52	7	7	2	11	25	0,4	0,4	Male	56	33,95000076	0	0	1	0	1	1	0
P152	S152	13	5	6	4	4	1			0,09	0,09	Male	50	39,84375	0	0	0	0	0	1	0
P153	S153	20	12	32	6	8	5	12	26	0,8		Male	73	25,95000076	1	0	1	1	1	1	0
P154	S154	17	10	19	7	8	2	14	33	0,2	0,6	Male	59	39,43999863	1	0	1	1	1	1	0
P155	S155	11	35	49	4	6	1	0		0,09	0,02	Male	55	31,44000053	0	0	1	0	0	1	0
P156	S156	6	1	4	4	4	1			0,02	0,09	Male	54	34,13999939	0	0	0	0	0	1	0
P157	S157	4	0	4	3	3	4			0,02	0,09	Male	62	25,68000031	0	0	0	0	0	0	0
P158	S158	13	1	5	3	4	0			0,02	0,02	Male	40	32,31000137	0	0	0	0	0	1	0
P159	S159	24	0	19	7	8	1	16	39	0,3		Male	58	30,46999931	1	0	1	1	1	1	0
P160	S160	19	77	87	7	7	3	12		0,5		Male	69	27,98999977	0	0	1	1	0	1	0
P161	S161	4	8	10	4	4	2			0,09	0,09	Female	52	38,06000137	0	0	0	0	0	1	0
P162	S162	22	85	99	7	7	2			0,5		Male	62	22,48999977	0	0	1	1	0	1	0
P163	S163	20	23	43	7	8	4	8	32	0,09		Female	63		1	0	1	1	1	1	0
P164	S164	12	7	8	4	4	2	0		0,2	0,02	Male	66	33,02000046	0	0	0	0	0	1	0

Supplementary Table 12. Overview of the performance of all models for outcome prognosis determined in this study.
Overview of the performance of all models for outcome prognosis determined in this study. MRM - Multiple Reaction Monitoring; SOFA - Sequential Organ Failure Assessment; APACHEII - Acute Physiology And Chronic Health Evaluation; CCI - Charlson Comorbidity Index; ABCS - age, biomarkers, clinical history, sex mortality risk score;

	**MRM panel**	**SOFA**	**APACHE II**	**CCI**	**ABCS (first sample)**	**ABCS (admission)**
AUROC	0,855	0,790	0,762	0,681	0,797	0,767
Accuracy	0,817	0,769	0,696	0,631	0,745	0,793
Balanced Accuracy	0,798	0,739	0,699	0,675	0,761	0,748
Specificity	0,831	0,833	0,689	0,600	0,734	0,814
Sensitivity	0,765	0,645	0,708	0,750	0,788	0,682
Precision	0,542	0,667	0,548	0,324	0,433	0,417
Precision (calibrated)	0,819	0,795	0,695	0,652	0,748	0,786
pos. Likelihood ratio	4,519	3,871	2,277	1,875	2,966	3,669
neg. Likelihood ratio	0,283	0,426	0,423	0,417	0,289	0,391
F1	0,634	0,656	0,618	0,453	0,559	0,517
F1 (calibrated)	0,791	0,712	0,702	0,698	0,767	0,730

Supplementary_Table_13. Patient survival data for each timepoint (SVM)

	#Patients in hospital	#Patients will die (p:death)	#Patients will live (p:death)	#Patients with unknown time (death, p:death)	#Patients with unknown time (survival, p:death)	#Patients will die (p:survival)	#Patients will live (p:survival)	#Patients with unknown time (death, p:survival)	#Patients with unknown time (survival, p:survival)
**0**	164	26	22	0	0	8	108	0	0
**1**	160	26	22	0	0	7	105	0	0
**2**	149	25	22	0	0	7	95	0	0
**3**	145	25	22	0	0	7	91	0	0
**4**	137	24	21	0	0	7	85	0	0
**5**	125	23	21	0	0	7	74	0	0
**6**	116	23	19	0	0	7	67	0	0
**7**	104	21	15	0	0	7	61	0	0
**8**	95	21	14	0	0	7	53	0	0
**9**	87	21	13	0	0	7	46	0	0
**10**	85	20	13	0	0	7	45	0	0
**11**	79	17	12	0	0	7	43	0	0
**12**	76	17	12	0	0	7	40	0	0
**13**	70	15	12	0	0	6	37	0	0
**14**	64	15	11	0	0	6	32	0	0
**15**	59	15	11	0	0	5	28	0	0
**16**	57	13	11	0	0	5	28	0	0
**17**	53	13	10	0	0	5	25	0	0
**19**	52	13	10	0	0	4	25	0	0
**20**	50	12	10	0	0	4	24	0	0
**21**	46	12	9	0	0	4	21	0	0
**22**	45	12	9	0	0	4	20	0	0
**23**	44	12	8	0	0	4	20	0	0
**24**	42	11	8	0	0	4	19	0	0
**25**	39	10	8	0	0	4	17	0	0
**26**	37	9	8	0	0	4	16	0	0
**28**	35	8	8	0	0	4	15	0	0
**29**	33	8	8	0	0	4	13	0	0
**30**	32	8	8	0	0	3	13	0	0
**32**	31	7	8	0	0	3	13	0	0
**33**	29	7	8	0	0	2	12	0	0
**34**	28	6	8	0	0	2	12	0	0
**36**	25	6	8	0	0	2	9	0	0
**38**	24	6	8	0	0	2	8	0	0
**39**	23	6	8	0	0	2	7	0	0
**41**	22	5	8	0	0	2	7	0	0

**42**	19	5	7	0	0	2	5	0	0
**43**	18	5	7	0	0	1	5	0	0
**48**	16	4	6	0	0	1	5	0	0
**49**	15	4	6	0	0	1	4	0	0
**50**	14	4	5	0	0	1	4	0	0
**52**	13	4	4	0	0	1	4	0	0
**53**	12	3	4	0	0	1	4	0	0
**57**	11	2	4	0	0	1	4	0	0
**65**	9	2	3	0	0	0	4	0	0
**70**	8	1	3	0	0	0	4	0	0
**77**	6	1	1	0	0	0	4	0	0
**78**	5	1	1	0	0	0	3	0	0
**86**	3	1	1	0	0	0	1	0	0
**88**	2	1	0	0	0	0	1	0	0
**173**	1	1	0	0	0	0	0	0	0
**209**	0	0	0	0	0	0	0	0	0

Supplementary_Table_14. Patient survival data for each timepoint (ExtraTrees)

	#Patients in hospital	#Patients will die (p:death)	#Patients will live (p:death)	#Patients with unknown time (death, p:death)	#Patients with unknown time (survival, p:death)	#Patients will die (p:survival)	#Patients will live (p:survival)	#Patients with unknown time (death, p:survival)	#Patients with unknown time (survival, p:survival)
**0**	164	24	24	0	0	10	106	0	0
**1**	160	23	24	0	0	10	103	0	0
**2**	149	22	24	0	0	10	93	0	0
**3**	145	22	24	0	0	10	89	0	0
**4**	137	22	23	0	0	9	83	0	0
**5**	125	21	23	0	0	9	72	0	0
**6**	116	21	22	0	0	9	64	0	0
**7**	104	20	20	0	0	8	56	0	0
**8**	95	20	19	0	0	8	48	0	0
**9**	87	20	17	0	0	8	42	0	0
**10**	85	19	17	0	0	8	41	0	0
**11**	79	17	16	0	0	7	39	0	0
**12**	76	17	15	0	0	7	37	0	0
**13**	70	15	15	0	0	6	34	0	0
**14**	64	15	14	0	0	6	29	0	0
**15**	59	15	14	0	0	5	25	0	0
**16**	57	14	14	0	0	4	25	0	0
**17**	53	14	12	0	0	4	23	0	0
**19**	52	13	12	0	0	4	23	0	0
**20**	50	12	12	0	0	4	22	0	0
**21**	46	12	11	0	0	4	19	0	0
**22**	45	12	11	0	0	4	18	0	0
**23**	44	12	10	0	0	4	18	0	0
**24**	42	11	10	0	0	4	17	0	0
**25**	39	10	10	0	0	4	15	0	0
**26**	37	9	9	0	0	4	15	0	0
**28**	35	8	9	0	0	4	14	0	0
**29**	33	8	9	0	0	4	12	0	0
**30**	32	8	9	0	0	3	12	0	0
**32**	31	7	9	0	0	3	12	0	0
**33**	29	6	9	0	0	3	11	0	0
**34**	28	5	9	0	0	3	11	0	0
**36**	25	5	8	0	0	3	9	0	0
**38**	24	5	8	0	0	3	8	0	0
**39**	23	5	8	0	0	3	7	0	0
**41**	22	4	8	0	0	3	7	0	0

**42**	19	4	8	0	0	3	4	0	0
**43**	18	4	8	0	0	2	4	0	0
**48**	16	3	7	0	0	2	4	0	0
**49**	15	3	7	0	0	2	3	0	0
**50**	14	3	6	0	0	2	3	0	0
**52**	13	3	5	0	0	2	3	0	0
**53**	12	2	5	0	0	2	3	0	0
**57**	11	2	5	0	0	1	3	0	0
**65**	9	2	4	0	0	0	3	0	0
**70**	8	1	4	0	0	0	3	0	0
**77**	6	1	2	0	0	0	3	0	0
**78**	5	1	1	0	0	0	3	0	0
**86**	3	1	1	0	0	0	1	0	0
**88**	2	1	1	0	0	0	0	0	0
**173**	1	1	0	0	0	0	0	0	0
**209**	0	0	0	0	0	0	0	0	0

Supplementary_Table_15. Patient survival data for each timepoint (LogReg)

	#Patients in hospital	#Patients will die (p:death)	#Patients will live (p:death)	#Patients with unknown time (death, p:death)	#Patients with unknown time (survival, p:death)	#Patients will die (p:survival)	#Patients will live (p:survival)	#Patients with unknown time (death, p:survival)	#Patients with unknown time (survival, p:survival)
**0**	164	25	19	0	0	9	111	0	0
**1**	160	24	18	0	0	9	109	0	0
**2**	149	24	18	0	0	8	99	0	0
**3**	145	24	18	0	0	8	95	0	0
**4**	137	23	18	0	0	8	88	0	0
**5**	125	22	18	0	0	8	77	0	0
**6**	116	22	17	0	0	8	69	0	0
**7**	104	20	15	0	0	8	61	0	0
**8**	95	20	13	0	0	8	54	0	0
**9**	87	20	13	0	0	8	46	0	0
**10**	85	19	13	0	0	8	45	0	0
**11**	79	17	12	0	0	7	43	0	0
**12**	76	17	12	0	0	7	40	0	0
**13**	70	16	12	0	0	5	37	0	0
**14**	64	16	11	0	0	5	32	0	0
**15**	59	16	11	0	0	4	28	0	0
**16**	57	14	11	0	0	4	28	0	0
**17**	53	14	10	0	0	4	25	0	0
**19**	52	13	10	0	0	4	25	0	0
**20**	50	12	10	0	0	4	24	0	0
**21**	46	12	8	0	0	4	22	0	0
**22**	45	12	8	0	0	4	21	0	0
**23**	44	12	7	0	0	4	21	0	0
**24**	42	11	7	0	0	4	20	0	0
**25**	39	10	7	0	0	4	18	0	0
**26**	37	9	7	0	0	4	17	0	0
**28**	35	8	7	0	0	4	16	0	0
**29**	33	8	7	0	0	4	14	0	0
**30**	32	7	7	0	0	4	14	0	0
**32**	31	6	7	0	0	4	14	0	0
**33**	29	6	7	0	0	3	13	0	0
**34**	28	6	7	0	0	2	13	0	0
**36**	25	6	7	0	0	2	10	0	0
**38**	24	6	7	0	0	2	9	0	0
**39**	23	6	7	0	0	2	8	0	0
**41**	22	5	7	0	0	2	8	0	0

**42**	19	5	7	0	0	2	5	0	0
**43**	18	5	7	0	0	1	5	0	0
**48**	16	4	6	0	0	1	5	0	0
**49**	15	4	6	0	0	1	4	0	0
**50**	14	4	5	0	0	1	4	0	0
**52**	13	4	4	0	0	1	4	0	0
**53**	12	3	4	0	0	1	4	0	0
**57**	11	2	4	0	0	1	4	0	0
**65**	9	2	3	0	0	0	4	0	0
**70**	8	1	3	0	0	0	4	0	0
**77**	6	1	2	0	0	0	3	0	0
**78**	5	1	1	0	0	0	3	0	0
**86**	3	1	1	0	0	0	1	0	0
**88**	2	1	0	0	0	0	1	0	0
**173**	1	1	0	0	0	0	0	0	0
**209**	0	0	0	0	0	0	0	0	0

Supplementary References

1 Benjamini Y, Hochberg Y. Controlling the False Discovery Rate: A Practical and Powerful Approach to Multiple Testing. *J. R. Stat. Soc. Series B Stat. Methodol. [Royal Statistical Society, Wiley]* 1995;57:289–300.

2 Demichev V, Tober-Lau P, Lemke O, Nazarenko T, Thibeault C, Whitwell H, et al. A time-resolved proteomic and prognostic map of COVID-19. *Cell Syst* 2021.

3 Charlson ME, Pompei P, Ales KL, MacKenzie CR. A new method of classifying prognostic comorbidity in longitudinal studies: development and validation. *J. Chronic Dis.* 1987;40:373–83.

4 Ferreira FL, Bota DP, Bross A, Mélot C, Vincent JL. Serial evaluation of the SOFA score to predict outcome in critically ill patients. *JAMA* 2001;286:1754–8.

5 Knaus WA, Draper EA, Wagner DP, Zimmerman JE. APACHE II: a severity of disease classification system. *Crit. Care Med.* 1985;13:818–29.

6 Jiang M, Li C, Zheng L, Lv W, He Z, Cui X, et al. A biomarker-based age, biomarkers, clinical history, sex (ABCS)-mortality risk score for patients with coronavirus disease 2019. *Ann Transl Med* 2021;9:230.

7 Pedregosa F, Varoquaux G, Gramfort A, Michel V, Thirion B, Grisel O, et al. Scikit-learn: Machine Learning in Python. *J. Mach. Learn. Res.* 2011;12:2825–30.

8 Siblini W, Fréry J, He-Guelton L, Oblé F, Wang Y-Q. Master Your Metrics with Calibration. Advances in Intelligent Data Analysis XVIII, *Springer International Publishing*; 2020, p. 457–69.

9 Virtanen P, Gommers R, Oliphant TE, Haberland M, Reddy T, Cournapeau D, et al. SciPy 1.0: fundamental algorithms for scientific computing in Python. *Nat. Methods* 2020;17:261–72.

10 Davidson-Pilon C. lifelines: survival analysis in Python. *J. Open Source Softw. The Open Journal* 2019;4:1317.

11 Platt JC. Probabilistic Outputs for Support Vector Machines and Comparisons to Regularized Likelihood Methods. ADVANCES IN LARGE MARGIN CLASSIFIERS, 1999.

12 Pfeiffer RM, Gail MH. Estimating the decision curve and its precision from three study designs. *Biom. J.* 2020;62:764–76.

13 Vickers AJ, Elkin EB. Decision curve analysis: a novel method for evaluating prediction models. *Med. Decis. Making* 2006;26:565–74.

14 Vickers AJ, Cronin AM, Elkin EB, Gonen M. Extensions to decision curve analysis, a novel method for evaluating diagnostic tests, prediction models and molecular markers. *BMC Med. Inform. Decis. Mak.* 2008;8:53.

15 Kurth F, Roennefarth M, Thibeault C, Corman VM, Müller-Redetzky H, Mittermaier M, et al. Studying the pathophysiology of coronavirus disease 2019: a protocol for the Berlin prospective COVID-19 patient cohort (Pa-COVID-19). *Infection* 2020;48:619–26.

16 Mühlemann B, Thibeault C, Hillus D, Helbig ET, Lippert LJ, Tober-Lau P, et al. Impact of dexamethasone on SARS-CoV-2 concentration kinetics and antibody response in hospitalized COVID-19 patients: results from a prospective observational study. *Clin. Microbiol. Infect.* 2021;27:1520.e7–1520.e10.

17 Thibeault C, Mühlemann B, Helbig ET, Mittermaier M, Lingscheid T, Tober-Lau P, et al. Clinical and virological characteristics of hospitalised COVID-19 patients in a German tertiary care centre during the first wave of the SARS-CoV-2 pandemic: a prospective observational study. *Infection* 2021;49:703–14.

18 Messner CB, Demichev V, Bloomfield N, Yu JSL, White M, Kreidl M, et al. Ultra-fast proteomics with Scanning SWATH. *Nat. Biotechnol.* 2021.
